# Supplementary material for: Modular assembly of an artificially concise biocatalytic cascade for the manufacture of phenethylisoquinoline alkaloids
Source: Nat Commun. 2024 Jan 2;15:30. doi: 10.1038/s41467-023-44420-7 (PMC10761944; doi:10.1038/s41467-023-44420-7)
Supplement: Supplementary file 1 — Supplementary information [file 41467_2023_44420_MOESM1_ESM.pdf]

Supplementary Information

Modular assembly of an artificially concise biocatalytic cascade for the manufacture of phenethylisoquinoline alkaloids

Yue Gao[1,3], Fei Li[1,3], Zhengshan Luo[1,3], Zhiwei Deng[1], Yan Zhang[2], Zhenbo Yuan[1], Changmei Liu[1], and Yijian Rao[1,*]

[1]Key Laboratory of Carbohydrate Chemistry and Biotechnology, Ministry of Education, School of Biotechnology, Jiangnan University, Wuxi 214122, P. R. China.

[2]School of Life Sciences and Health Engineering, Jiangnan University, Wuxi 214122, P. R. China. [3]These authors contributed equally: Yue Gao, Fei Li and Zhengshan Luo

[*]Correspondence and requests for materials should be addressed to Y.R. (email: raoyijian@jiangnan.edu.cn

Table of contents

The nucleotide and protein sequences in this study 3
Characterization of substrates and products.. 11
Supplementary Tables 19
Supplementary Figures... 29

The nucleotide sequences used in this study

The nucleotide sequence of *Tp*CAR.

Codon-optimized nucleotide sequence of *Tp*CAR expressed in *E. coli* BL21 (*DE3*) [3318 bp]:

ATGAGTATTGAGACCGTACAAAATGGCGTGCCCGCCGAGGGGTCTGTCCCCCCAGCCG
ACCAACAAACTGAACGTTTGCCCCAAGTTATTGCTCGCATTTTCGCTCAATTTGCGGAC
CGCCCCGCATTTGCCACGCGCGAGGCCGGTCCTGGTACCCCTTATGCGACCGTGTCCTA
TCGTGAGATTTGGCGTCGCGTCACGGCACTTGTAGCCTCTTGGCAATCTGAGGTAGCC
CCAGGTGACTTCGTGGCGATCTTGGGATTCACATCTTCTGATTTTGTTACTGTCGATTTA
GCTACTACACTTTTAGGAGCTCCAAACGTTCCACTGCAGGCTGGCGCACCAGCCGCGC
GCATCGCAACCATCCTTGATGAAACCCGTCCAAAAATTTTGGCCGTTAGCGCCGATCAG
GTCGATTTAGCCCAGGAAGCTTTAGCTGAATCAGCTGCCACGCCGCGTGTTGTCGTTTT
CGATGGTGAGCGTGACGGCTATGAAGGGATCGAAGCCGATATTTTGTCCGGTAGCGCTT
TACCGGCTCCGGAGTTTTTCGCGCCAGAGCCAGGGACTGATCCGCTTGTGACGTTAAT
CTACACTTCAGGTTCAACCGGCACGCCGAAGGGCGCCATGTACACAGAGCAGCTGGTA
CGTGACGCGTGGTTGAAAGTCGACTCGATTGTTGATATCGACATGCCCGCAGAGTCATT
GCTTCATTTTCTGCCCATGTCGCACATGTATGGTCGCAATTGGCTTATCGCTGGCCTTGC
GTCTGGAGGGACGGGATACTTCGCCGGGGCATCGGATATGAGTACCTTATTCGATGACC
TGGCGGCTGCACGCCCCACCGCCATTGGCTTAGTGCCACGTGTTTGTGAATTAATCCAC
CAGCGCTACCTTGCGGTAGAGGCAGATACGGACGCGGAGACGGCTCGCGTAGAGTTG
CGTGACCGCGTCCTGGGTGGACGTTTGCAAGCAGCTATGTGTGGAAGTGCTGCGCTGT
CATCAGAGTTACAAACATTCATGGAATGGCTTTTAGGCATTGACATTCAAATTGGATAC
GGAAGTACCGAAGCGGGGGGTGTCATCCGCGACGGGGTAGTAGTCCGCCCACCGGTG
ACAGAGTATAAACTGATCGATGTCCCAGAATTAGGATATTTTGTCACTGACAGCCCTCA
TCCGCGCGGGGAGCTTTTGGTGAAATCGACACAGTTGATCCCCGGGTACTATAACAGC
GACAAGCGCATCCGTGATGACGAAGGTTTTTATCGTACCGGCGACGTGATGGCTGAAT
TGGGACCAGACCGCTTGGAGTACGTCGATCGTCGTAGCAATGTGATCAAGTTGGCACA
GGGTGAATTTGTACCGATTGCGCAGTTAGAAGCTATTTATGCGGCAGGACCCGATGTGC
ATCAGATCTTTCTTTACGGGACCAGCGAACGCAGCTATTTGATCGGGGTCGTGGTACCC
GCGCCAGGCCCGGATGGAGAAACGGACGCGCAAACCCGTACTCGCGTGTTGGACGGA

49 CTGGCGGCTATTGCCCGCGAAAACGACTTGGCCGCTTATGAAGTTCCCCGTGACGTCT

50 TGATTGAACGTGATCCCTTTTCGCAGGAGAACGGACTGCGTAGTGGAATCGGTAAGCT

51 TGTACGCCCTGCACTTATCGCGCGCTACGGTGACCGTCTGCATGATTTATACGCACAAG

52 CGGACACACGTCAACGCGAAGGCTTACGTGCCTTGGATGCGTCCGGACCAATCATCGA

53 CACTGTCTTAGGTGCGGCTGCCTTAACCTTAGGCGCTGACATCGCCGATTTTGATGCTG

54 ATACACGCTTCGGAGACTTGGGTGGGGATTCATTGTCTGCGCTTAGCTTAGCAACTACA

55 TTGGAAGGGCTGTACGACGTTCCAGTACCAGTACAAACGATCGTCGGGCCGACAGCA

56 ACTTTAGGAGGTGTAGCACGTCACATCGAGAAGGCTCGTTCAGGAGGCGTGGCTGCCC

57 CCACCGCCGACAGTGTGCACGGGGTTGGAGCCAGCGTAGCACGTGCGACGGACTTGA

58 CATTGGAAAAGTTTATCGATCCAGAACTGCTTGCTTTAGCTCCAACATTGCCGGCCGCG

59 ACCGGTGAGCCAAATACTGTACTTTTAACAGGTTCGACGGGTTATCTTGGGCGCTTTCT

60 GTTATTAGATTGGTTGCGTCGTGTGGCGCCACATGGGGGGACTGTTATTGCTTTGGTGC

61 GTGGGGCGGATGCCGACGACGCACGCCGTCGTGTGACTGCGGCGATCGGAGATTCTG

62 ACCCCGACCTTACTCAAGAGTTCACGAGTCTGGCTGAGCATCACCTGCATGTGATTGCT

63 GGCGACTTTGGGTCGCCGGCCTTAGGATTGGATGACGCGACCTGGTCGGATCTTGCAG

64 GACGTGTAGATCATGTAGTACATTGTGGGGCTCTTGTTAATCATGTCCTGCCATACGACC

65 AGTTATTTGGGCCAAACGTTGTCGCAACTGGTGAGGTTGTCCGTTTAGCGTTGACGAC

66 ACGCCGCAAGTCAGTCGACTACGTGTCCACTGTCGCTGTCGTTCCTCAGGATGATGGG

67 CGTGTCCTGGTTGAAGATGATGATGTACGTGAGTTAGGTGCGGAGCGCCGCATCGGCG

68 CGGACGCTTATGCTAACGGCTACGCCGTGTCGAAATGGGCGGGGGAGGTTCTGTTGCA

69 TGAGGCAGCAGACTTAGCTGATCTGCCAGTACGCGTGTTCCGCTCCGATATGATTTTAG

70 CGCACTCTCGTTTTCACGGTCAATTTAATGAAGTTGACCAATTTACCCGCCTGCTTCTTT

71 CTATTGCGGAAACTGGGTTAGCTCCCGCCTCTTTCTACACACCTGACCCGAGCGGTCAT

72 CGTCCGCACTACGATGGTTTACCTGTTGACTTTACCGCTGAGGCAATTACAACTCTGTC

73 AGCGGCAGGGCGTTCTGGATACCGCACCTTCCACGTTTTGAATGCGAATGATGATGGC

74 GTGTCACTTGACAGCTTCGTCGATTGGATTGCGGCAAGTGGCCGTAGCATTGAACGTAT

75 CGACGACTATGATACTTGGTTTGCCCGCTTTGAGCAGGCATTACAACAATTACCCGACG

76 AAGCGCGCCAGCGTAGCGTGTTACCTCTTTTGCACGCAGTGCGTGAGCCTGCGCCCGC

77 GGCGGGAACGTCCGCTTTATCGGTGGATCGCTTCCGTGGTGCCGTACGTGAAACTGGT

78 GTGGGTCCAGGAGACATCCCAGTTCTTGACCGTGCACTTATCGAGAAGTATTTGCGTG

ATTTCGAGACCGCGGGTTGGCTTGCCCCGGGTGCACGTGACTAA

The nucleotide sequence of *Bs*Sfp.

Codon-optimized nucleotide sequence of *Bs*Sfp expressed in *E. coli* BL21 (*DE3*) [675 bp]:

ATGAAGATTTACGGAATTTATATGGACCGCCCGCTTTCACAGGAAGAAAATGAACGGTTCATGTCTTTCATATCACCTGAAAAACGGGAGAAATGCCGGAGATTTTATCATAAAGAAGATGCTCACCGCACCCTGCTGGGAGATGTGCTCGTTCGCTCAGTCATAAGCAGGCAGTATCAGTTGGACAAATCCGATATCCGCTTTAGCACGCAGGAATACGGGAAGCCGTGCATCCCTGATCTTCCCGACGCTCATTTCAACATTTCTCACTCCGGACGCTGGGTCATTTGCGCGTTTGATTCACAGCCGATCGGCATAGATATCGAAAAAACGAAACCGATCAGCCTTGAGATCGCCAAGCGCTTCTTTTCAAAAACAGAGTACAGCGACCTTTTAGCAAAAGACAAGGACGAGCAGACAGACTATTTTTATCATCTATGGTCAATGAAAGAAAGCTTTATCAAACAGGAAGGCAAAGGCTTATCGCTTCCGCTTGATTCCTTTTCAGTGCGCCTGCACCAGGACGGACAAGTATCCATTGAGCTTCCGGACAGCCATTCCCCATGCTATATCAAAACGTATGAGGTCGATCCCGGCTACAAAATGGCTGTATGCGCCGTACACCCTGATTTCCCCGAGGATATCACAATGGTCTCGTACGAAGAGCTTTTATGA

The nucleotide sequence of *Tf*NCS.

Codon-optimized nucleotide sequence of *Tf*NCS expressed in *E. coli* BL21 (*DE3*) [549 bp]:

ATGCTGCATCACCAAGGGATTATCAACCAAGTCTCAACCGTCACTAAGGTAATCCACCACGAACTGGAAGTCGCTGCTAGCGCGGACGACATCTGGACCGTGTACTCCTGGCCGGGTCTGGCGAAACACTTACCGGACCTGCTTCCAGGGGCCTTCGAGAAACTTGAGATCATCGGAGACGGGGGGGTTGGGACAATCCTGGACATGACTTTCGTACCGGGGGAGTTTCCACACGAATATAAGGAAAAGTTCATTCTGGTTGACAACGAACATCGCTTAAAGAAAGTGCAAATGATTGAAGGAGGCTATCTTGACTTAGGTGTAACTTACTATATGGATACGATCCACGTTGTTCCAACTGGAAAAGACAGTTGTGTCATTAAGTCAAGTACTGAGTATCATGTCAAGCCGGAGTTCGTAAAGATTGTTGAGCCGCTTATTACAACTGGACCACTGGCCGCTATGGCCGATGCCATTTCGAAACTTGTTCTTGAACATAAGTCCAAGTCGAACTCCGACGAGATCGAAGCTGCGATCATCACTGTCTAA

The nucleotide sequence of *Rn*COMT.

Codon-optimized nucleotide sequence of *Rn*COMT expressed in *E. coli* BL21 (*DE3*) [666 bp]:

ATGGGAGATACCAAAGAACAGCGCATCTTGCGCTACGTACAGCAGAATGCAAAACCAG
GCGACCCTCAGTCAGTGTTGGAGGCCATCGACACGTACTGCACCCAGAAAGAATGGG
CTATGAACGTTGGTGATGCAAAGGGACAGATCATGGATGCTGTGATCCGCGAATATTCT
CCGTCTTTGGTATTAGAATTAGGAGCATACTGTGGTTACTCCGCGGTACGTATGGCGCGT
CTTTTGCAACCAGGTGCACGCTTATTGACGATGGAAATGAACCCCGATTATGCCGCGAT
TACACAACAAATGTTAAATTTCGCAGGGTTACAAGATAAAGTCACAATTCTTAACGGTG
CATCCCAGGATTTAATTCCCCAATTAAAAAAAAAGTATGATGTAGACACATTGGATATG
GTATTTTTGGACCATTGGAAGGACCGCTATTTACCTGATACATTACTGCTTGAGAAGTGT
GGACTTTTACGCAAGGGGACTGTATTATTGGCTGACAATGTAATCGTTCCTGGAACACC
AGACTTCTTGGCTTACGTCCGCGGTTCTAGTAGCTTTGAATGCACTCACTACTCGTCTT
ATCTTGAGTACATGAAAGTTGTCGACGGTCTTGAGAAAGCGATCTACCAAGGTCCTTC
TAGCCCTGATAAATCTTAA

The nucleotide sequence of CNMT.

Codon-optimized nucleotide sequence of CNMT expressed in *E. coli* BL21 (*DE3*) [1059 bp]:

ATGCAAACAAAAAAAGCCGCAATCGTTGAATTGCTGAAGCAGCTTGAATTGGGGCTG
GTGCCGTATGACGACATCAAGCAGTTAATCCGCCGTGAGCTGGCGCGCCGCCTGCAGT
GGGGGTATAAGCCAACGTATGAAGAGCAAATCGCGGAAATCCAAAATCTGACCCACTC
GTTGCGTCAGATGAAGATTGCTACTGAAGTCGAGACGCTTGATAGTCAGTTGTACGAA
ATCCCAATTGAGTTTTTGAAGATTATGAACGGAAGCAACTTGAAGGGAAGTTGTTGCT
ACTTCAAAGAAGATTCAACCACGTTAGATGAGGCTGAAATCGCTATGTTGGATTTGTAC
TGTGAACGTGCTCAAATTCAGGACGGGCAAAGTGTGCTGGATCTTGGATGCGGCCAGG
GAGCCCTGACGCTTCATGTTGCACAGAAATATAAAAATTGTCGCGTAACGGCTGTCAC
AAATTCCGTTAGTCAGAAGGAGTACATCGAGGAAGAATCGCGTCGCCGCAACCTGCTG
AATGTGGAGGTGAAATTAGCAGACATTACAACGCACGAGATGGCCGAGACTTACGACC
GTATTTTAGTAATCGAGCTGTTTGAACACATGAAAAACTATGAGCTGTTGCTTCGTAAA
ATTTCCGAGTGGATCTCAAAAGATGGACTTTTATTTTTAGAGCATATCTGCCATAAAACT
TTTGCGTATCATTATGAGCCCTTAGATGATGACGACTGGTTCACGGAGTATGTATTTCCT

GCAGGAACTATGATTATTCCATCTGCTTCTTTCTTTTTGTATTTCCAGGATGACGTGAGTGTCGTCAACCATTGGACTTTGTCAGGTAAACACTTTTCCCGTACAAACGAGGAATGGCTGAAACGCTTGGATGCCAACCTGGACGTAATTAAACCCATGTTCGAGACATTAATGGGGAATGAGGAAGAAGCAGTGAAGTTAATCAACTATTGGCGCGGGTTCTGCTTAAGCGGGATGGAGATGTTCGGATATAATAATGGTGAAGAGTGGATGGCATCACATGTTCTTTTCAAGAAATGA

The nucleotide sequence of *Gs*OMT1.

Codon-optimized nucleotide sequence of *Gs*OMT1 expressed in *E. coli* BL21 (*DE3*) [1086 bp]:

ATGGCAAGTGCGCCCATCCACGCTGGAGCTACCCCCGAAGAGGAGGAGGAGAAGGCTCTTTTTTTGTCTGCAATGCGCCTGACGTCTGCGGCTGGCTTACCCATGGTCGTGAAAGCTGCCATTGAATTGGACGTGTTAGAAATCATTGCTCGTGCGGGGCCGGGCGCACAACTTTCCGCTTCAGAAATTGCATCCCGTATTCCGGGAGCGGCATCATCCGAGGCACCCGCTGTGTTGGACCGTATGCTGCGTTTCTTGGCGTGCCACTCAGTACTGACGTCCACATCTGTGGATGGAAAAGAGCGTCGCTATGGCCTGGCCCCGGTTTGCCGTTTCTTAACCCGTGATAAAGATGGCGCGTCTATTGGTGATCAGGTTCTGGTAGACCTTGACCCCGTCTTACTTGCGTGCTGGGAGCAGATGAAGTATTTGGTCTTAGAGGGTATTTCTCCTTTCACCAAAGCGCACGGTACTACAGCATTTGAATATCACGGCGTAAACCCGCGCATCAATAAAGTTTTCAATAATGCGATGGCATCCAATTCTACAATTATTCTTCGTAAAATTCTGGAGATCTACAAAGGTTTTGAGGGTGTTACTCAGTTGGTGGATGTAGGGGGAGGTTTGGGTATCACTCTGAAAATGATTATTTCGAAATATCCGCACATCAAAGGGATTAATTATGATTTGCCGCACGTTATTAGTGACGCACCTACGTTACCTGGTGTTGAGAACATTGGAGGGGATATGTTCGTTGAAATTCCATCAGCTAGCACTATCTTTATGAAATGGATTCTGCATGACTGGAGCGACGAACACTGTCTGAAGATCCTGAAGAATTGTTGGAAAGCCCTGCCCAATGGCGGTAAAGCGATTATCATGGAGGCTATTTTGCCAGAAACACCAAAAACCGATGCCGCAAGCCAGGAGGCTTTCTATTCTGACCTTTGCATGTTGATTAGTAACGTCGGTGGCAAAGAACGCACCCGCAACGAGTTTAAGTCCTTAGCCATGGATGCAGGCTTTACCAAGTTTGAAACCGTGAGCTGCGCCTTGAATATTTGGGTCATGGAGCTGACAAAATAA

The nucleotide sequence of *Mm*SAHH.

Codon-optimized nucleotide sequence of *Mm*SAHH expressed in *E. coli* BL21 (*DE3*) [1299 bp]:

ATGTCTGACAAACTTCCCTATAAGGTAGCCGATATTGGCCTGGCCGCTTGGGGCCGCAA
AGCGCTGGACATCGCCGAGAATGAGATGCCCGGCCTGATGCGTATGCGTGAAATGTAC
AGCGCCAGTAAACCGCTGAAGGGGGCGCGCATTGCGGGTTGTTTACACATGACCGTAG
AGACCGCTGTACTTATTGAAACCCTTGTTGCATTGGGAGCGGAAGTACGTTGGTCCTCA
TGTAATATCTTCAGCACTCAAGACCATGCAGCTGCCGCAATTGCCAAGGCGGGTATCCC
GGTATTCGCTTGGAAAGGGGAGACAGATGAAGAGTATCTGTGGTGCATTGAACAGACA
TTACATTTCAAGGATGGTCCTCTTAACATGATTCTTGATGATGGGGGAGACCTTACGAA
CTTAATCCACACGAAATACCCACAGCTTCTGTCAGGAATCCGTGGAATCTCAGAAGAG
ACAACCACAGGAGTTCACAACTTGTACAAAATGATGAGCAATGGGATTTTGAAAGTGC
CAGCGATCAACGTGAACGATTCCGTAACTAAGAGCAAATTCGATAACCTGTACGGCTG
CCGTGAATCGTTGATCGACGGAATTAAGCGCGCGACCGATGTGATGATTGCGGGGAAG
GTTGCGGTAGTTGCGGGGTATGGGGACGTAGGTAAGGGGTGCGCGCAAGCACTTCGC
GGTTTTGGAGCTCGTGTAATTATCACAGAGATTGACCCTATTAATGCTCTGCAAGCAGC
GATGGAGGGGTACGAAGTCACGACCATGGATGAGGCGTGCAAGGAGGGTAATATTTTC
GTCACAACCACGGGCTGTGTAGACATCATTTTAGGGCGTCACTTCGAACAAATGAAGG
ATGATGCCATTGTGTGTAACATCGGTCATTTCGATGTGGAGATCGACGTTAAGTGGCTT
AACGAAAATGCAGTAGAAAAAGTCAACATCAAACCACAGGTGGACCGTTATTGGCTTA
AGAATGGGCGTCGTATCATCCTGCTTGCCGAGGGTCGTTTAGTCAACCTTGGGTGTGCG
ATGGGCCACCCCTCTTTCGTAATGTCCAATAGCTTCACAAATCAAGTGATGGCTCAGAT
CGAACTGTGGACGCATCCGGACAAATATCCCGTAGGAGTTCATTTTTTGCCGAAAAAG
TTGGATGAGGCCGTGGCGGAAGCACATCTTGGAAAACTGAATGTAAAATTGACTAAAT
TGACTGAAAAGCAAGCGCAATATTTAGGTATGCCGATCAACGGCCCTTTCAAACCCGA
CCATTATCGTTATTAA

The nucleotide sequence of *Gs*NMTt.

Codon-optimized nucleotide sequence of *Gs*NMTt expressed in *E. coli* BL21 (*DE3*) [894 bp]:

ATGAGTCATGAGGCAGCAGCCGTTGAGGAGGTCAACAAGGGCATCGCGGACCTGTAT
GACGAGATGACCGTTGTCATGGAAACGCTTTGGGGTGATCACATGCACCATGGATTCTA
CGACGTAGGCGTACCTACCCGCTCCTTGCCCGATCACCAGACAGCCCAGATCCGCATG
ATTGAGGAAGCGTTACGTTTTGCCGGAGTTTCAGATGATCCTAGTAAGAAGCCAAAGC

GTATTTTAGACGTCGGTTGTGGCATCGGTGGCTCGTCGCTGTACTTAGCAAAAAAGTAC
GGGGCTAAGTGTCATGGCATCAACTTGTCCCCTTTCCAAATCCAACGTGCTCAGTCTTT
GGCAATGAGTGCGGGTTTAACCGATAAGGTATCCTTTGAAATCGCAGACGCCCAAAAC
CAGCCCTTCCCAGATGGGCACTTCGATTTAGTCTGGGTCTTGGAGACCGCGGAACACA
TGCCGGAAAAATCGAAGTTCATTGGCGAGTTAGCTCGTGTTACTGCGCCCGGCGGCAT
TGTGATCATCACTTCCTGGTGCCAGCGCGATTTGTTGCCTAGTGAAGTTTCGTTGCGCC
CCGATGAGGTTTCTCTGTTAAACAAACTTTCAGAAAGCCACCATTTGCCCAAATGGTG
CTCGCCATCAGAGTACGTTAAGTTGGCTGAATCGTCCAGCTTCAAAGATATTAAAACCG
CAGACTGGACAGAACATGTTGCCCCACATTGGAACGTAGCAATGCGCCCTGTACTGAC
TTGGAAAGGGATTACCTTCATTCTGCGCAGTGGGTGGAAGATGGCGAAGGGTCTTCTT
GCAATGCCCATTGTGTCCGAAGGACGCGAGAAGAAGATTATTAAATACGCGATCTTGA
CTTTCCGTAAACCGGAGTGA

The nucleotide sequence of PavNMT.

Codon-optimized nucleotide sequence of PavNMT expressed in *E. coli* BL21 (*DE3*) [1047 bp]:

ATGGCAGTAGCCAACTTGATTAAACGTATTGAACATGGGGAAGTTTCAGATGAAGAGA
TTCGCGGAATGATGAAAATTCAAGTGCAGAAGCGTTTAAAATGGGGATACAAGCCGAC
TCATGAACAACAACTTGCACAGCTGGTAACCTTTGCTCAGTCATTAAAGGGGATGGAG
ATGGCGGAGGAGGTGGACACGCTGGATGCCGAGTTGTACGAAATCCCTCTTCCATTCT
TGCACATCATGTGTGGCAAGACCCTTAAGTTCTCACCCGGATATTTCAAAGATGAATCA
ACCACGTTGGATGAGTCAGAAGTCTACATGATGGACTTATATTGCGAACGCGCGCAGAT
CAAAGACGGACAGTCCATCCTTGATCTGGGTTGCGGCCACGGCTCATTAACCTTGCAT
GTGGCTCAGAAGTATCGTGGGTGCAAGGTTACCGGAATTACCAACAGCGTCTCCCAGA
AAGAGTTCATTATGGATCAGTGTAAAAAATTGGATCTGAGTAATGTGGAAATTATTCTG
GAAGATGTAACCAAGTTTGAGACAGAAATTACCTACGATCGTATCTTCGCAGTCGCATT
AATTGAGCACATGAAGAACTATGAGTTATTTTTAAAGAAGGTCTCCACATGGATTGCCC
AGGATGGCCTTCTGTTTGTAGAGCATCACTGTCATAAAGTCTTCGCTTATCAGTACGAA
CCACTTGACGAAGATGACTGGTATACTGAGTACATTTTTCCTAGCGGCACCCTGGTAAT
GAGCAGTTCCAGCATCTTGCTTTATTTTCAAGAAGACGTGTCGGTCGTCAACCACTGG
ACGCTTTCTGGGAAACATCCATCGCTGGGATTTAAGCAGTGGCTTAAACGTCTGGACG

ACAACATCGATGAAGTAAAAGAAATTTTCGAGAGTTTCTATGGTTCAAAAGAAAAGGC
CATGAAGTTTATCACGTACTGGCGCGTGTTTTGCATCGCACACTCTCAGATGTACTCAA
CCAATAACGGAGAGGAATGGATGTTGTCCCAGGTCTTGTTTAAGAAAAAATGA

The nucleotide sequence of TNMT.

Codon-optimized nucleotide sequence of TNMT expressed in *E. coli* BL21 (*DE3*) [1053 bp]:

ATGAAAGAATCTATCGGGGAAATCATGGGCAAACTTATGCAGGGAGAGATCGGTGACG
AAGAGCTTTCGAAACGTATTAAGGAAATCTTCGGCAAACGCCTTCAGTGGGGCTACAA
ACCTACGCATCAACAACAGCTTGCCTTTAACTTGGACTTCATCAAATCGCTTAAAGAGA
TGGATATGTCGGGTGAAATTGACACTATGAACGAGGAAACCTATGAGCTGCCAAGCGC
ATTCCTTGAAGCCGCGTTCGGTAAAACTATCAAGCAGAGTGGTTGCTACTTTAAAGAC
GAAACGACTACGATCGATGAAGCGGAGGAGGCCTCACACGAGTTATATTGCGAACGTG
CGCAGATTAAGGATGGACAAACAGTGCTGGACATTGGATGCGGACAAGGTGGTTTAGT
TTTACACATCGCACAAAAATACAAAAACTGCCATGTGACTGGCCTTACTAACTCCAAA
GCTCAAAAAAATTATATCCTGATGCAGGCTGAAAAATTGCAACTTTCTAACGTCGATGT
CATTCTTGCTGACGTGACAAAGCACGAGAGCGATAAAACGTATGACCGCATCCTTGTC
ATCGAGACTATTGAACACATGAAGAACATCCAGCTTTTTATGAAAAAACTTAGCACATG
GATGACGGAAGATAGCCTTTTATTCGTCGATCATATCTGCCACAAGACATTCTCTCACCA
CTTTGAAGCCATTGATGAAGATGATTGGTATTCCGGTTTTATTTTTCCTAAAGGTTGCGT
GACTATTCTGAGCGCGAGTGCACTGTTATATTTTCAGGATGATGTAACCATTCTGGATCA
CTGGGTGGTCAACGGCATGCACATGGCACGCTCAGTGGATGCATGGCGTAAAAAACTT
GACAAGAATATGGAGTTGGCTCGCGAGATTCTGCTGCCTGGTTTAGGCTCGAAGGAGG
CGGTGAATGGAGTCATTACACATATCCGTACATTCTGCATGGGCGGTTACGAACAATTC
TCCTATAACAATGGGGAAGAATGGATGGTGGCCCAAATGTTATTTAAGAAGAAATGA

Characterization of substrates and products

(*S*)-1-(3,4,5-trimethoxyphenethyl)-1,2,3,4-tetrahydroisoquinoline-6,7-diol ((*S*)-1b)

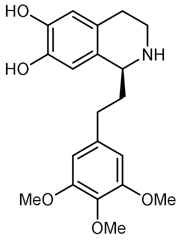

$C_{20}H_{25}NO_5$: 359.4220 g/mol

^{1}H NMR (400 MHz, DMSO-*d*$_6$) δ 9.42 (s, 1H), 9.16 (br, 1H), 8.95 (s, 1H), 6.64 (s, 1H), 6.57 (s, 3H), 4.31 (s, 1H), 3.77 (s, 6H), 3.63 (s, 3H), 3.47 – 3.43 (m, 1H), 3.29 – 3.24 (m, 1H), 2.92 – 2.63 (m, 4H), 2.22 – 2.08 (m, 2H) ppm.

^{13}C NMR (101 MHz, DMSO-*d*$_6$) δ 153.3, 145.5, 144.8, 137.0, 136.2, 123.3, 122.7, 115.7, 113.7, 106.0, 60.4, 56.2, 54.2, 39.3, 36.0, 31.8, 24.7 ppm.

Exact Mass [M+H]$^+$: 360.1805 (calculated), 360.1821 (found).

(*S*)-6-methoxy-2-methyl-1-(3,4,5-trimethoxyphenethyl)-1,2,3,4-tetrahydroisoquinolin-7-ol ((*S*)-1d)

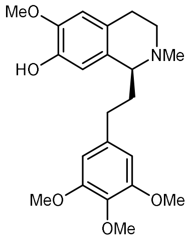

$C_{22}H_{29}NO_5$: 387.4760 g/mol

^{1}H NMR (600 MHz, Methanol-*d*$_4$) δ 6.57 (s, 1H), 6.48 (s, 1H), 6.38 (s, 2H), 3.72 (s, 3H), 3.70 (s, 6H), 3.61 (s, 3H), 3.47 (t, *J* = 5.6 Hz, 1H), 3.15 (td, *J* = 11.1, 10.6, 6.6 Hz, 1H), 2.76 – 2.66 (m, 3H), 2.56 (ddd, *J* = 13.6, 10.4, 6.3 Hz, 1H), 2.46 – 2.41 (m, 4H), 2.04 – 1.98 (m, 1H), 1.97 – 1.91 (m, 1H) ppm.

^{13}C NMR (151 MHz, Methanol-d4) δ 153.0, 146.7, 144.6, 138.4, 135.8, 128.0, 124.1, 113.4, 111.3, 105.3, 62.8, 59.7, 55.2, 55.0, 41.1, 39.0, 35.8, 31.9, 24.4 ppm.

Exact Mass [M+H]$^+$: 388.2118 (calculated), 388.2127 (found).

(*S*)-6-methoxy-2-methyl-1-phenethyl-1,2,3,4-tetrahydroisoquinolin-7-ol ((*S*)-2d)

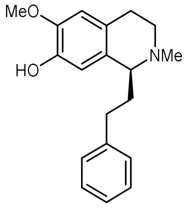

$C_{19}H_{23}NO_2$: 297.1729 g/mol

^1H NMR (600 MHz, Methanol-*d*$_4$) δ 7.25 (t, *J* = 7.6 Hz, 2H), 7.19 – 7.13 (m, 3H), 6.68 (s, 1H), 6.59 (s, 1H), 3.82 (s, 3H), 3.61 (t, *J* = 5.4 Hz, 1H), 3.28 – 3.25 (m, 1H), 2.88– 2.78 (m, 3H), 2.71 (ddd, *J* = 13.6, 10.9, 6.0 Hz, 1H), 2.59 (ddd, *J* = 13.7, 10.9, 5.2 Hz, 1H), 2.55 (s, 3H), 2.12 (ddt, *J* = 16.1, 10.7, 5.4 Hz, 1H), 2.02 (ddt, *J* = 14.1, 11.4, 5.9 Hz, 1H) ppm. For ^1H-NMR spectrum see Fig. S21, which is consistent to the literature reported[1].

Exact Mass [M+H]$^+$: 298.1802 (calculated), 298.1796 (found).

(*S*)-1-(2-chlorophenethyl)-6-methoxy-2-methyl-1,2,3,4-tetrahydroisoquinolin-7-ol ((*S*)-3d)

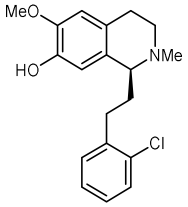

$C_{19}H_{22}ClNO_2$: 331.1339 g/mol

^1H NMR (600 MHz, Methanol-*d*$_4$) δ 7.36 (dd, *J* = 7.8, 1.1 Hz, 1H), 7.32 (dd, *J* = 7.5, 1.5 Hz, 1H), 7.25 (td, *J* = 7.4, 1.2 Hz, 1H), 7.21 (td, *J* = 7.6, 1.7 Hz, 1H), 6.82 (s, 1H), 6.69 (s, 1H), 4.31 (t, *J* = 6.0 Hz, 1H), 3.86 (s, 3H), 3.67 (dt, *J* = 13.3, 7.9 Hz, 1H), 3.38 (dt, *J* = 12.5, 5.5 Hz, 1H), 3.10 – 3.07 (m, 2H), 2.93 (s, 3H), 2.91 – 2.88 (m, 2H), 2.29 (ddd, *J* = 16.7, 8.2, 5.3 Hz, 1H), 2.17 – 2.11 (m, 1H) ppm.

^{13}C NMR (151 MHz, Methanol-*d*$_4$) δ 148.2, 145.6, 138.1, 133.4, 130.3, 129.3, 127.9, 127.1, 123.1, 121.1, 113.6, 111.4, 63.5, 55.1, 46.7, 39.6, 33.3, 29.5, 22.4 ppm.

Exact Mass [M+H]$^+$: 332.1412 (calculated), 332.1407 (found).

(*S*)-1-(3-chlorophenethyl)-6-methoxy-2-methyl-1,2,3,4-tetrahydroisoquinolin-7-ol ((*S*)-4d)

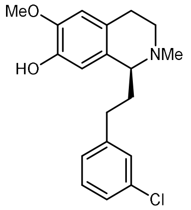

$C_{19}H_{22}ClNO_2$: 331.1339 g/mol

^{1}H NMR (400 MHz, Methanol-d_4) δ 7.31 – 7.27 (m, 2H), 7.23 (d, *J* = 8.1 Hz, 1H), 7.18 (d, *J* = 7.3 Hz, 1H), 6.85 (s, 1H), 6.68 (s, 1H), 4.33 (t, *J* = 5.7 Hz, 1H), 3.88 (s, 3H), 3.85 – 3.80 (m, 1H), 3.43 – 3.40 (m, 1H), 3.17 – 3.10 (m, 2H), 2.97 (s, 3H), 2.78 (t, *J* = 8.2 Hz, 2H), 2.32 (dq, *J* = 13.7, 7.9 Hz, 1H), 2.21 (dq, *J* = 14.2, 7.2 Hz, 1H) ppm.

^{13}C NMR (151 MHz, Methanol- d_4) δ 148.3, 145.8, 142.5, 134.1, 129.8, 128.0, 126.5, 126.2, 121.7, 120.6, 113.8, 111.5, 63.8, 55.1, 46.0, 39.6, 36.3, 30.9, 21.3 ppm.

Exact Mass [M+H]$^+$: 332.1412 (calculated), 332.1429 (found).

(*S*)-1-(4-chlorophenethyl)-6-methoxy-2-methyl-1,2,3,4-tetrahydroisoquinolin-7-ol ((*S*)-5d)

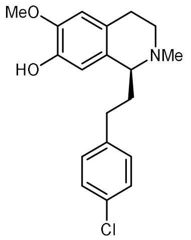

$C_{19}H_{22}ClNO_2$: 331.1339 g/mol

^{1}H NMR (400 MHz, Methanol-d_4) δ 7.31 (d, *J* = 8.4 Hz, 2H), 7.24 (d, *J* = 8.2 Hz, 2H), 6.85 (s, 1H), 6.68 (d, *J* = 9.2 Hz, 1H), 4.32 (t, *J* = 6.0 Hz, 1H), 3.88 (s, 3H), 3.85 – 3.78 (m, 1H), 3.42 (dt, *J* = 12.3, 5.2 Hz, 1H), 3.17 – 3.09 (m, 2H), 2.97 (s, 3H), 2.80 – 2.76 (m, 2H), 2.31 (dq, *J* = 14.3, 7.7 Hz, 1H), 2.20 (dq, *J* = 15.4, 7.5 Hz, 1H) ppm.

^{13}C NMR (151 MHz, Methanol-d_4) δ 152.2, 149.7, 142.9, 135.8, 133.6, 132.3, 125.7, 124.4, 117.7, 115.4, 67.7, 59.0, 49.9, 43.5, 40.4, 34.6, 25.3 ppm.

Exact Mass [M+H]$^+$: 332.1412 (calculated), 332.1421 (found).

(*S*)-1-(4-fluorophenethyl)-6-methoxy-2-methyl-1,2,3,4-tetrahydroisoquinolin-7-ol ((*S*)-6d)

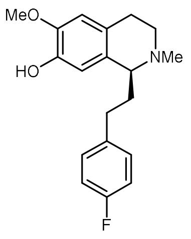

$C_{19}H_{22}FNO_2$: 315.1635 g/mol

^{1}H NMR (400 MHz, Methanol-*d*$_4$) δ 7.24 (s, 2H), 7.02 (t, *J* = 8.2 Hz, 2H), 6.83 (s, 1H), 6.66 (s, 1H), 4.30 (s, 1H), 3.87 (s, 3H), 3.84 – 3.79 (m, 1H), 3.42 – 3.36 (m, 1H), 3.10 – 3.05 (m, 2H), 2.95 (s, 3H), 2.77 (t, *J* = 8.0 Hz, 2H), 2.35 – 2.26 (m, 1H), 2.21 – 2.13 (m, 1H) ppm.

^{13}C NMR (151 MHz, Methanol-*d*$_4$) δ 162.4, 160.8, 148.3, 145.7, 136.1, 129.7, 121.8, 120.5, 115.0, 114.8, 113.8, 111.4, 63.8, 55.0, 45.9, 39.5, 36.7, 30.5, 21.3 ppm.

Exact Mass [M+H] $^+$: 316.1707 (calculated), 316.1693 (found).

(*S*)-1-(4-bromophenethyl)-6-methoxy-2-methyl-1,2,3,4-tetrahydroisoquinolin-7-ol ((*S*)-7d)

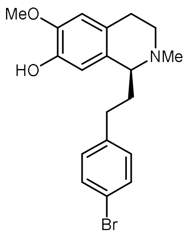

$C_{19}H_{22}BrNO_2$: 375.0834 g/mol

^{1}H NMR (400 MHz, Methanol-*d*$_4$) δ 7.47 (d, *J* = 8.3 Hz, 2H), 7.19 (d, *J* = 7.8 Hz, 2H), 6.85 (s, 1H), 6.68 (s, 1H), 4.33 (t, *J* = 5.6 Hz, 1H), 3.89 (s, 3H), 3.85 – 3.78 (m, 1H), 3.44 – 3.38 (m, 1H), 3.14 – 3.10 (m, 2H), 2.97 (s, 3H), 2.77 (t, *J* = 8.3 Hz, 2H), 2.35 – 2.26 (m, 1H), 2.25 – 2.19 (m, 1H) ppm.

^{13}C NMR (151 MHz, Methanol-*d*$_4$) δ 148.3, 145.8, 139.4, 131.4, 130.0, 121.7, 120.5, 119.8, 113.8 111.4, 63.8, 55.0, 46.0, 39.5, 36.4, 30.7, 21.3 ppm.

Exact Mass [M+H] $^+$: 376.0907 (calculated), 376.0926 (found).

(*S*)-6-methoxy-2-methyl-1-(4-methylphenethyl)-1,2,3,4-tetrahydroisoquinolin-7-ol ((*S*)-8d)

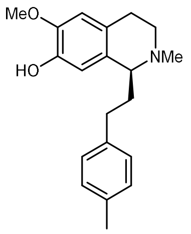

$C_{20}H_{25}NO_2$: 311.1885 g/mol

^{1}H NMR (400 MHz, Methanol-*d*$_4$) δ 7.12 (s, 4H), 6.83 (s, 1H), 6.65 (s, 1H), 4.28 (q, *J* = 8.4, 6.1 Hz, 1H), 3.88 (s, 3H), 3.79 (ddd, *J* = 13.0, 8.8, 6.8 Hz, 1H), 3.43 – 3.38 (m, 1H), 3.17 – 3.08 (m, 2H), 2.94 (s, 3H), 2.74 (t, *J* = 8.2 Hz, 2H), 2.35 – 2.26 (m, 4H), 2.18 (dt, *J* = 14.1, 7.3 Hz, 1H) ppm.

^{13}C NMR (151 MHz, Methanol-*d*$_4$) δ 152.2, 149.6, 140.9, 139.7, 132.9, 131.8, 125.9, 124.4, 117.7, 115.4, 67.7, 59.0, 49.8, 43.4, 40.7, 34.8, 25.2, 23.6 ppm.

Exact Mass [M+H]$^+$: 312.1958 (calculated), 312.1950 (found).

(*S*)-6-methoxy-1-(4-methoxyphenethyl)-2-methyl-1,2,3,4-tetrahydroisoquinolin-7-ol ((*S*)-9d)

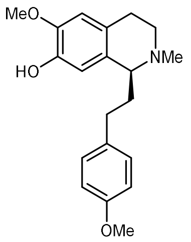

$C_{20}H_{25}NO_3$: 327.1834 g/mol

^{1}H NMR (400 MHz, Methanol-d4) δ 7.12 (d, J = 8.6 Hz, 2H), 6.83 (d, J = 8.6 Hz, 2H), 6.72 (s, 1H), 6.61 (s, 1H), 3.84 (s, 3H), 3.78 (t, J = 5.8 Hz, 1H), 3.75 (s, 3H), 3.39 (dt, J = 13.1, 7.2 Hz, 1H), 3.04 – 2.98 (m, 1H), 2.95 – 2.82 (m, 2H), 2.73 – 2.56 (m, 5H), 2.16 (ddt, J = 15.8, 10.7, 5.3 Hz, 1H), 2.03 (ddd, J = 14.1, 10.5, 7.0 Hz, 1H) ppm.

^{13}C NMR (151 MHz, Methanol-*d*$_4$) δ 158.1, 147.2, 144.9, 133.3, 129.0, 126.3, 122.9, 113.6, 113.5, 111.4, 62.9, 55.1, 54.3, 47.1, 40.5, 35.9, 30.7, 23.6 ppm.

Exact Mass [M+H]$^+$: 328.1907 (calculated), 328.1913 (found).

(*S*)-6-methoxy-1-(3-methoxyphenethyl)-2-methyl-1,2,3,4-tetrahydroisoquinolin-7-ol ((*S*)-10d)

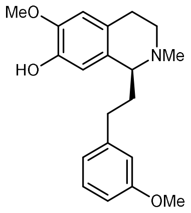

$C_{20}H_{25}NO_3$: 327.1834 g/mol

^{1}H NMR (600 MHz, Methanol-*d*4) δ 7.19 (t, *J* = 7.6 Hz, 1H), 6.80– 6.74 (m, 4H), 6.63 (s, 1H), 4.24 (s, 1H), 3.85 (s, 3H), 3.80 – 3.75 (m, 4H), 3.39 – 3.35 (m, 1H), 3.12 – 3.03 (m, 2H), 2.91 (s, 3H), 2.73 (t, *J* = 8.2 Hz, 2H), 2.31 (dq, *J* = 13.4, 7.9 Hz, 1H), 2.16 (dt, *J* = 13.8, 7.2 Hz, 1H) ppm.

^{13}C NMR (151 MHz, Methanol-d_4) δ 160.1, 148.2, 145.7, 141.7, 129.4, 121.9, 120.6, 120.3, 113.8, 113.7, 111.5, 111.5, 63.8, 55.1, 54.2, 45.8, 39.5, 36.6, 31.3, 21.3 ppm.

Exact Mass [M+H] $^+$: 328.1907 (calculated), 328.1913 (found).

(*S*)-1-(3,4-dimethoxyphenethyl)-6-methoxy-2-methyl-1,2,3,4-tetrahydroisoquinolin-7-ol ((*S*)-11d)

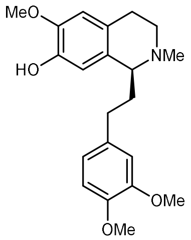

$C_{21}H_{27}NO_4$: 357.1940 g/mol

^{1}H NMR (400 MHz, Methanol-d_4) δ 6.90 (d, J = 8.1 Hz, 1H), 6.84 (s, 2H), 6.80 (d, J = 8.1 Hz, 1H), 6.66 (s, 1H), 4.29 (t, J = 6.0 Hz, 1H), 3.88 (s, 3H), 3.84 (s, 3H), 3.81 – 3.78 (m, 4H), 3.44 – 3.39 (m, 1H), 3.19 – 3.09 (m, 2H), 2.96 (s, 3H), 2.74 (t, J = 8.1 Hz, 2H), 2.37 – 2.28 (m, 1H), 2.26 – 2.16 (m, 1H) ppm.

^{13}C NMR (151 MHz, Methanol-d_4) δ 149.3, 148.2, 147.8, 145.7, 132.9, 121.9, 120.4, 120.3, 113.8, 112.0, 112.0, 111.4, 63.8, 55.2, 55.1, 55.0, 45.9, 39.5, 36.8, 30.9, 21.3 ppm.

Exact Mass [M+H] $^+$: 358.2013 (calculated), 358.2020 (found).

3-Hydroxy-4,5-dimethoxybenzenepropanoic acid (12)

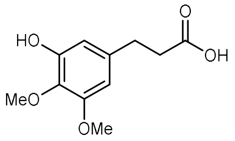

$C_{11}H_{14}O_5$: 226.2280 g/mol

^{1}H NMR (400 MHz, DMSO-*d_6*) δ 12.09 (s, 1H), 8.98 (s, 1H), 6.33 (dd, *J* = 16.5, 1.5 Hz, 2H), 3.73 (s, 3H), 3.62 (s, 3H), 2.67 (t, *J* = 7.6 Hz, 2H), 2.47 (d, *J* = 7.6 Hz, 2H) ppm. For ^{1}H-NMR spectrum see Fig. S31, which is consistent to the literature reported[2].

Exact Mass [M-H] $^{-}$: 225.0768 (calculated), 225.0769 (found).

(*S*)-1-(3-hydroxy-4,5-dimethoxyphenethyl)-1,2,3,4-tetrahydroisoquinoline-6,7-diol ((*S*)-12b)

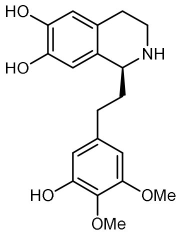

$C_{19}H_{23}NO_5$: 345.1576 g/mol

^{1}H NMR (600 MHz, Methanol-*d_4*) δ 6.67 (s, 1H), 6.65 (s, 1H), 6.43 (d, *J* = 1.1 Hz, 2H), 4.40 (dd, *J* = 7.4, 5.2 Hz, 1H), 3.85 (s, 3H), 3.77 (s, 3H), 3.57 (dt, *J* = 12.5, 6.2 Hz, 1H), 3.40 – 3.36 (m, 1H), 3.02 (dt, *J* = 17.0, 6.2 Hz, 1H), 2.95 (dt, *J* = 17.0, 6.3 Hz, 1H), 2.76 – 2.66 (m, 2H), 2.30 (ddt, *J* = 15.5, 10.8, 6.1 Hz, 1H), 2.23 – 2.16 (m, 1H) ppm.

^{13}C NMR (151 MHz, Methanol-*d_4*) δ 153.4, 150.3, 145.5, 144.6, 136.3, 134.9, 122.5, 122.3, 114.9, 112.6, 108.7, 103.6, 59.6, 55.0, 54.7, 39.4, 35.7, 31.1, 24.3 ppm.

Exact Mass [M+H] $^{+}$: 346.1649 (calculated), 346.1642 (found).

(*S*)-autumnaline ((*S*)-12d)

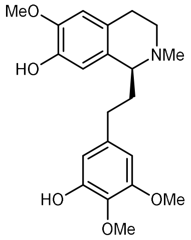

$C_{21}H_{27}NO_5$: 373.1889 g/mol

^{1}H NMR (400 MHz, Methanol-d_4) δ 6.84 (s, 1H), 6.65 (s, 1H), 6.39 (s, 2H), 4.28 (t, J = 6.3 Hz, 1H), 3.88 (s, 3H), 3.83 – 3.79 (m, 4H), 3.76 (s, 3H), 3.44 – 3.38 (m, 1H), 3.12 (dd, *J* = 17.9, 8.6 Hz, 2H), 2.95 (s, 3H), 2.68 (t, J = 8.1 Hz, 2H), 2.35 – 2.26 (m, 1H), 2.20 (dt, J = 14.5, 7.6 Hz, 1H) ppm.

^{13}C NMR (151 MHz, Methanol-d_4) δ 153.4, 150.3, 148.2, 145.7, 136.1, 134.9, 121.9, 120.5, 113.8, 111.4, 108.7, 103.6, 63.8, 59.6, 55.0, 55.0, 45.9, 39.5, 36.6, 31.3, 21.3 ppm.

Exact Mass [M+H] $^+$: 374.1962 (calculated), 374.1972 (found).

Supplementary Tables

Supplementary Table 1. Kinetic parameters of wild-type *Gs*OMT1 and *Gs*OMT1^{V125Q} towards (*S*)-1-(3-hydroxy-4,5-dimethoxyphenethyl)-1,2,3,4-tetrahydroisoquinoline-6,7-diol ((*S*)-**12b**).

**Enzyme**	K_m **(mM)**	k_{cat} **(s^{-1})**	k_{cat} / K_m **(mM^{-1}·s^{-1})**
WT	0.63 ± 0.05	4.56 ± 0.24	7.29 ± 0.23
V125Q	0.33 ± 0.02	8.65 ± 0.26	26.50 ± 0.51

Supplementary Table 2. The information of the regents used in this study.

Regents	Purity (%)	Catalog numbers	Source
Primers			Exsyn-bio (Wuxi, China)
DNA marker		3584A	TaKaRa
Protein marker		3597A	TaKaRa
PrimerSTAR MAX DNA Polymerase		R045Q	TaKaRa
2 × Phanta Flash Master Mix		P510-01	Vazyme
2 × MultiF Seamless Assembly Mix		RK21020	ABclonal (Wuhan, China)
Tryptone		LP0042B	OXOID
Yeast extract		LP0021T	OXOID
NaCl	99.8	10019308	China National Pharmaceutical Group Corp (Shanghai, China)
Acetonitrile (HPLC)	99.9	80988AM	Adamas-Beta (Shanghai, China)
Methanol (HPLC)	99.9	75851AR	Adamas-Beta (Shanghai, China)
3-(3,4,5-trimethoxyphenyl)propanoic acid (**1**)	98	A011166	Energy Chemical (Shanghai, China)
3-phenylpropanoic acid (**2**)	98	A010214	Energy Chemical (Shanghai, China)
3-(2-chlorophenyl)propanoic acid (**3**)	98	A0112270050	Energy Chemical (Shanghai, China)
3-(3-chlorophenyl)propanoic acid (**4**)	98	A011745	Energy Chemical (Shanghai, China)
3-(4-chlorophenyl)propanoic acid (**5**)	98	A011140	Energy Chemical (Shanghai, China)
3-(4-fluorophenyl)propanoic acid (**6**)	98	A010757	Energy Chemical (Shanghai, China)
3-(4-bromophenyl)propanoic acid (**7**)	98	A010420	Energy Chemical (Shanghai, China)
3-(*p*-tolyl)propanoic acid (**8**)	98	A011107	Energy Chemical (Shanghai, China)
3-(4-methoxyphenyl)propanoic acid (**9**)	98	A010827	Energy Chemical (Shanghai, China)
3-(3-methoxyphenyl)propanoic acid (**10**)	98	A010503	Energy Chemical (Shanghai, China)
3-(3,4-dimethoxyphenyl)propanoic acid (**11**)	98	A010228	Energy Chemical (Shanghai, China)
3-hydroxy-4,5-dimethoxybenzaldehyde	>97	45930B	Adamas-Beta (Shanghai, China)
Malonic acid	98	A010411	Energy Chemical (Shanghai, China)

2-(3,4-Dihydroxyphenyl)ethylamine hydrochloride	98	E080183	Energy Chemical (Shanghai, China)

441 **Supplementary Table 3.** All plasmids used in this study.

Plasmid	Description	Source
pET-21b(+)	T7 promoter, pBR332 ori, Amp^R	Novagen
pRSFDuet-1	double T7 promoters, RSF ori, Kan^R, >100 copy number	Novagen
pETDuet-1	double T7 promoters, pBR322 ori, Amp^R, ~40 copy number	Novagen
pCDFDuet-1	double T7 promoters, CDF ori, Str^R, 20-40 copy number	Novagen
pEcCas	Native cas9 promoter, rhaB promoter, araBAD promoter, pSC101 ori, Kan^R	Addgene
pTargetF	pij23119 promoter, pBR332 ori, $Spec^R$	Addgene
pTargetF-*dkgB*	pTargetF carring sgRNA of gene *dkgB*	This study
pTargetF-*yeaE*	pTargetF carring sgRNA of gene *yeaE*	This study
pTargetF-*yahK*	pTargetF carring sgRNA of gene *yahK*	This study
pTargetF-*yjgB*	pTargetF carring sgRNA of gene *yjgB*	This study
pTargetF-*yqhC*	pTargetF carring sgRNA of gene *yqhC*	This study
pRSFDuet-1-*Tp*CAR/*Bs*Sfp	pRSFDuet-1 carring *Tp*CAR (6His in N-terminum) and *Bs*Sfp (No tag in N/C-terminum)	This study
pET-21b(+)-*Tf*NCS	pET-21b(+) carring *Tf*NCS (6His in C-terminum)	This study
pET-21b(+)-*Rn*COMT	pET-21b(+) carring *Rn*COMT (6His in C-terminum)	This study
pET-21b(+)-*Gs*OMT1	pET-21b(+) carring *Gs*OMT1 (6His in C-terminum)	This study
pET-21b(+)-CNMT	pET-21b(+) carring CNMT (6His in C-terminum)	This study
pET-21b(+)-GDH	pET-21b(+) carring GDH (6His in C-terminum)	This study
pET-21b(+)-*Gs*NMTt	pET-21b(+) carring *Gs*NMTt (6His in C-terminum)	This study
pET-21b(+)-PavNMT	pET-21b(+) carring PavNMT (6His in C-terminum)	This study
pET-21b(+)-TNMT	pET-21b(+) carring TNMT (6His in C-terminum)	This study
pRSFDuet-1-*Tp*CAR/*Bs*Sfp-*Tf*NCS	pRSFDuet-1 carring *Tp*CAR, *Bs*Sfp and *Tf*NCS	This study
pETDuet-1-*Tp*CAR/*Bs*Sfp-*Tf*NCS	pETDuet-1 carring *Tp*CAR, *Bs*Sfp and *Tf*NCS	This study
pCDFuet-1-*Tp*CAR/*Bs*Sfp-*Tf*NCS	pCDFDuet-1 carring *Tp*CAR, *Bs*Sfp and *Tf*NCS	This study
pRSFDuet-1-*Tp*CAR/*Bs*Sfp	pRSFDuet-1 carring *Tp*CAR and *Bs*Sfp	This study
pETDuet-1-*Tp*CAR/*Bs*Sfp	pETDuet-1 carring *Tp*CAR and *Bs*Sfp	This study
pCDFDuet-1-*Tp*CAR/*Bs*Sfp	pCDFDuet-1 carring *Tp*CAR and *Bs*Sfp	This study
pRSFDuet-1-*Tf*NCS	pRSFDuet-1 carring *Tf*NCS	This study
pETDuet-1-*Tf*NCS	pETDuet-1 carring *Tf*NCS	This study

pCDFDuet-1-*Tf*NCS	pCDFDuet-1 carring *Tf*NCS	This study
pRSFDuet-1-*Ec*MAT/*Rn*COMT	pRSFDuet-1 carring *Ec*MAT and *Rn*COMT	This study
pETDuet-1-*Ec*MAT/*Rn*COMT	pETDuet-1 carring *Ec*MAT and *Rn*COMT	This study
pCDFDuet-1-*Ec*MAT/*Rn*COMT	pCDFDuet-1 carring *Ec*MAT and *Rn*COMT	This study
pRSFDuet-1-CNMT*/*Mm*SAHH	pRSFDuet-1 carring CNMT$^{N92A/F332A}$ and *Mm*SAHH	This study
pETDuet-1-CNMT*/*Mm*SAHH	pETDuet-1 carring CNMT$^{N92A/F332A}$ and *Mm*SAHH	This study
pCDFDuet-1-CNMT*/*Mm*SAHH	pCDFDuet-1 carring CNMT$^{N92A/F332A}$ and *Mm*SAHH	This study
pRSFDuet-1-*Ec*MAT/*Rn*COMT-*Mm*SAHH	pRSFDuet-1 carring *Ec*MAT, *Rn*COMT and *Mm*SAHH	This study
pETDuet-1-*Ec*MAT/*Rn*COMT-*Mm*SAHH	pETDuet-1 carring *Ec*MAT, *Rn*COMT and *Mm*SAHH	This study
pCDFDuet-1-*Ec*MAT/*Rn*COMT-*Mm*SAHH	pCDFDuet-1 carring *Ec*MAT, *Rn*COMT and *Mm*SAHH	This study
pRSFDuet-1-CNMT*/*Mm*SAHH-*Ec*MAT	pRSFDuet-1 carring CNMT$^{N92A/F332A}$, *Mm*SAHH and *Ec*MAT	This study
pETDuet-1-CNMT*/*Mm*SAHH-*Ec*MAT	pETDuet-1 carring CNMT$^{N92A/F332A}$, *Mm*SAHH and *Ec*MAT	This study
pCDFDuet-1-CNMT*/*Mm*SAHH-*Ec*MAT	pCDFDuet-1 carring CNMT$^{N92A/F332A}$, *Mm*SAHH and *Ec*MAT	This study

Supplementary Table 4. All strains used in this study.

Strain	Description	Source
E. coli BL21 (*DE3*)	*E. coli* str. B F^- *ompT gal dcm lon hsdS*$_B$ (r_B^- m_B^-) *λ*(*DE3* [*lacI lacUV5-T7p07 ind1 sam7 nin5*]) [*malB*$^+$]$_{K\text{-}12}$(λ^S)	Novagen
IAA	*E. coli* BL21 (*DE3*), Δ*dkgB*, Δ*yeaE*, Δ*(yqhC-dkgA)*, Δ*yahK*, Δ*yjgB*	This study
WT	pRSFDuet-1-*Tp*CAR/*Bs*Sfp-*Tf*NCS was expressed in *E. coli* BL21 (*DE3*)	This study
M1A	pRSFDuet-1-*Tp*CAR/*Bs*Sfp and pETDuet-1-*Tf*NCS were co-expressed in IAA	This study
M1B	pRSFDuet-1-*Tp*CAR/*Bs*Sfp and pCDFDuet-1-*Tf*NCS were co-expressed in IAA	This study
M1C	pETDuet-1-*Tp*CAR/*Bs*Sfp and pRSFDuet-1-*Tf*NCS were co-expressed in IAA	This study
M1D	pETDuet-1-*Tp*CAR/*Bs*Sfp and pCDFDuet-1-*Tf*NCS were co-expressed in IAA	This study
M1E	pCDFDuet-1-*Tp*CAR/*Bs*Sfp and pRSFDuet-1-*Tf*NCS were co-expressed in IAA	This study
M1F	pCDFDuet-1-*Tp*CAR/*Bs*Sfp and pETDuet-1-*Tf*NCS were co-expressed in IAA	This study
M1G	pRSFDuet-1-*Tp*CAR/*Bs*Sfp-*Tf*NCS was expressed in IAA	This study
M1H	pETDuet-1-*Tp*CAR/*Bs*Sfp-*Tf*NCS was expressed in IAA	This study
M1I	pCDFDuet-1-*Tp*CAR/*Bs*Sfp-*Tf*NCS was expressed in IAA	This study
S1	pRSFDuet-1-*Ec*MAT/*Rn*COMT and pET-CNMT*/*Mm*SAHH were co-expressed in IAA	This study
S2	pRSFDuet-1-*Ec*MAT/*Rn*COMT and pCDF-CNMT*/*Mm*SAHH were co-expressed in IAA	This study
S3	pETDuet-1-*Ec*MAT/*Rn*COMT and pRSF-CNMT*/*Mm*SAHH were co-expressed in IAA	This study
S4	pETDuet-1-*Ec*MAT/*Rn*COMT and pCDF-CNMT*/*Mm*SAHH were co-expressed in IAA	This study
S5	pCDFDuet-1-*Ec*MAT/*Rn*COMT and pRSF-CNMT*/*Mm*SAHH were co-expressed in IAA	This study
S6	pCDFDuet-1-*Ec*MAT/*Rn*COMT and pET-CNMT*/*Mm*SAHH were co-expressed in IAA	This study
M2A	pRSFDuet-1-*Ec*MAT/*Rn*COMT-*Mm*SAHH was expressed in IAA	This study
M2B (*Rn*COMT)	pETDuet-1-*Ec*MAT/*Rn*COMT-*Mm*SAHH was expressed in IAA	This study
M2B (*Gs*OMT1^{V125Q})	pETDuet-1-*Ec*MAT/*Gs*OMT1^{V125Q}-*Mm*SAHH was expressed in IAA	This study
M2B (sumo-*Gs*OMT1^{V125Q})	pETDuet-1-*Ec*MAT/sumo-*Gs*OMT1^{V125Q}-*Mm*SAHH was expressed in IAA	This study
M2C	pCDFDuet-1-*Ec*MAT/*Rn*COMT-*Mm*SAHH was expressed in IAA	This study
M3A	pRSFDuet-1-CNMT*/*Mm*SAHH-*Ec*MAT was expressed in RIAA	This study
M3B	pETDuet-1-CNMT*/*Mm*SAHH-*Ec*MAT was expressed in IAA	This study
M3C	pCDFDuet-1-CNMT*/*Mm*SAHH-*Ec*MAT was expressed in IAA	This study

Supplementary Table 5. All primers used in this study.

Name	Sequence (5'-3')
Duet-F (MCS2)	TAATTAACCTAGGCTGCTGCCAC
CAR-R	ATGTATATCTCCTTCTTAAAGTTTAGTCACGTGCACCCGGG
Sfp-F	TAAGAAGGAGATATACATATGAAGATTTACGGAATTTATATGGACCGC
Sfp-R	AGCAGCCTAGGTTAATTATAAAAGCTCTTCGTACGAGACCATTGTG
NCS-F	ATAAGGAGATATACCATGCTGCATCACCAAGGGATTATC
NCS-R	TTCTGTTCGACTTAAGCATTAGACAGTGATGATCGCAGCTTCGATC
Duet-R (MCS2)	CATATGTATATCTCCTTCTTATACTTAACTAATATACTAAGATGGG
NCS-1-F	GAAGGAGATATACATATGCTGCATCACCAAGGGATTATCAACC
NCS-2-R	AGCAGCCTAGGTTAATTAGACAGTGATGATCGCAGCTTCG
Duet-F (MCS1)	TGCTTAAGTCGAACAGAAAGTAATCGTATTG
Duet-R (MCS1)	CATGGTATATCTCCTTATTAAAGTTAAACAAAATTATTTCTACAGG
MAT-F	TTAATAAGGAGATATACCATGGCAAAACACCTTTTTACGTCCG
MAT-R	CCCATATGTATATCTCCTTCTTAAAGTTcACTTCAGACCGGCAGCATC
COMT-F	GAAGGAGATATACATATGGGAGATACCAAAGAACAGCGCATC
COMT-R	TTCTGTTCGACTTAAGCATTAAGATTTATCAGGGCTAGAAGGACCTTG
CNMT-F	TTAATAAGGAGATATACCatgCAAACAAAAAAAGCCGCAATCG
CNMT-R	ATGTATATCTCCTTCTTAAAGTTcATTTCTTGAAAAGAACATGTGATGCC ATCC
SAHH-F	TAAGAAGGAGATATACATATGTCTGACAAACTTCCCTATAAGGTAGC
SAHH-R	TTCTGTTCGACTTAAGCATcAATAACGATAATGGTCGGGTTTGAAAGG
21b(+)-F	CTCGAGCACCACCACCAC
21b(+)-R	CATATGTATATCTCCTTCTTAAAGTTAAACAAAATTATTTCTAGAGGG
*Gs*NMTt-F	GAAGGAGATATACATATGAGTCATGAGGCAGCAGCC
*Gs*NMTt-R	GTGGTGGTGGTGCTCGAGCTCCGGTTTACGGAAAGTCAAGATC
CNMT (L88A)-F	TTGAGTTT**GCG**AAGATTATGAACGGAAGCAACTTGAAGG
CNMT (L88A)-R	TAATCTT**CGC**AAACTCAATTGGGATTTCGTACAACTGAC
CNMT (M91A)-F	TGAAGATT**GCG**AACGGAAGCAACTTGAAGGGAAG
CNMT (M91A)-R	TTCCGTT**CGC**AATCTTCAAAAACTCAATTGGGATTTCGTACAAC
CNMT (N92A)-F	AGATTATG**GCT**GGAAGCAACTTGAAGGGAAGTTGTTG
CNMT (N92A)-R	TGCTTCC**AGC**CATAATCTTCAAAAACTCAATTGGGATTTCGTACAAC
CNMT (W297A)-F	ACGAGGAA**GCT**CTGAAACGCTTGGATGCCAACC
CNMT (W297A)-R	GTTTCAG**AGC**TTCCTCGTTTGTACGGGAAAAGTGTTTAC
CNMT (W329A)-F	TCAACTAT**GCC**CGCGGGTTCTGCTTAAGCG
CNMT (W329A)-R	ACCCGCG**GGC**ATAGTTGATTAACTTCACTGCTTCTTCCTC
CNMT (F332A)-F	GGCGCGGG**GCG**TGCTTAAGCGGGATGGAGATG
CNMT (F332A)-R	TTAAGCA**CGC**CCCGCGCCAATAGTTGATTAACTTC
CNMT (C333A)-F	GCGGGTTC**GCA**TTAAGCGGGATGGAGATGTTCGG
CNMT (C333A)-R	CGCTTAA**TGC**GAACCCGCGCCAATAGTTGATTAAC
CNMT (L88G)-F	TTGAGTTT**GGG**AAGATTATGAACGGAAGCAACTTGAAGG
CNMT (L88G)-R	TAATCTT**CCC**AAACTCAATTGGGATTTCGTACAACTGAC
CNMT (L88S)-F	TTGAGTTT**TCG**AAGATTATGAACGGAAGCAACTTGAAGG
CNMT (L88S)-R	TAATCTT**CGA**AAACTCAATTGGGATTTCGTACAACTGAC

CNMT (L88V)-F	TTGAGTTT**GTC**AAGATTATGAACGGAAGCAACTTGAAGG
CNMT (L88V)-R	TAATCTT**GAC**AAACTCAATTGGGATTTCGTACAACTGAC
CNMT (N92V)-F	AGATTATG**GTT**GGAAGCAACTTGAAGGGAAGTTGTTG
CNMT (N92V)-R	TGCTTCC**AAC**CATAATCTTCAAAAACTCAATTGGGATTTCGTACAAC
CNMT (F332S)-F	GGCGCGGG**TCC**TGCTTAAGCGGGATGGAGATGTTC
CNMT (F332S)-R	TTAAGCA**GGA**CCCGCGCCAATAGTTGATTAAC
CNMT (F332V)-F	GGCGCGGG**GTG**TGCTTAAGCGGGATGGAGATG
CNMT (F332V)-R	TTAAGCA**GGA**CCCGCGCCAATAGTTGATTAAC
*Gs*OMT1(I122N)-F	GGTGATGTGGTTCTGGTAGACCTTG
*Gs*OMT1(I122N)-R	TACCAGAACCACATCACC**GTT**AGACGCGCCATCTTTATCACGGG
*Gs*OMT1(I122Q)-R	TACCAGAACCACATCACC**CTG**AGACGCGCCATCTTTATCACGGG
*Gs*OMT1(I122D)-R	TACCAGAACCACATCACC**GTC**AGACGCGCCATCTTTATCACGGG
*Gs*OMT1(V125N)-F	GTTCTGGTAGACCTTGACCCCG
*Gs*OMT1(V125N)-R	GTCAAGGTCTACCAGAAC**GTT**ATCACCAATAGACGCGCCATC
*Gs*OMT1(V125Q)-R	GTCAAGGTCTACCAGAAC**CTG**ATCACCAATAGACGCGCCATC
*Gs*OMT1(V125D)-R	GTCAAGGTCTACCAGAAC**ATC**ATCACCAATAGACGCGCCATC
*Gs*OMT1(V125E)-R	GTCAAGGTCTACCAGAAC**TTC**ATCACCAATAGACGCGCCATC
*Gs*OMT1(V125H)-R	GTCAAGGTCTACCAGAAC**GTG**ATCACCAATAGACGCGCCATC
*Gs*OMT1(N181A)-F	TCTACAATTATTCTTCGTAAAATTCTGGAGATCTACAAAGG
*Gs*OMT1(N181A)-R	ACGAAGAATAATTGTAGA**GGC**GGATGCCATCGCATTATTGAAAACT TTATTG
*Gs*OMT1(N181Q)-R	ACGAAGAATAATTGTAGA**CTG**GGATGCCATCGCATTATTGAAAACTT TATTG
*Gs*OMT1(E310V)-F	GCTTTCTATTCTGACCTTTGCATGTTGATTAG
*Gs*OMT1(E310V)-R	AAGGTCAGAATAGAAAGC**GAC**CTGGCTTGCGGCATCGGTTTTTG
*Gs*OMT1(E310L)-R	AAGGTCAGAATAGAAAGC**CAA**CTGGCTTGCGGCATCGGTTTTTG
dkgB-KO-F	TTGATCTGACTCTAATCCACGTTTTAGAGCTAGAAATAGC
dkgB-KO-R	GTGGATTAGAGTCAGATCAAACTAGTATTATACCTAGGACTGAGC
yeaE-KO-F	TTGAGTACGATCTACTCCCCGTTTTAGAGCTAGAAATAGCAAGTT
yeaE-KO-R	GGGGAGTAGATCGTACTCAAACTAGTATTATACCTAGGACTGAGC
yahK-KO-F	TCATGAAATTGTGGGGCGTGGTTTTAGAGCTAGAAATAGCAAGTT
yahK-KO-R	CACGCCCCACAATTTCATGAACTAGTATTATACCTAGGACTGAGC
yjgB-KO-F	AAAAGAAGCGGGCGGAGAACGTTTTAGAGCTAGAAATAGCAAGTT
yjgB-KO-R	GTTCTCCGCCCGCTTCTTTTACTAGTATTATACCTAGGACTGAGC
yqhC/yqhD/dkgA-KO-F	TGTACTCGCATAGCCGCAAGGTTTTAGAGCTAGAAATAGCAAGTT
yqhC/yqhD/dkgA-KO-R	CTTGCGGCTATGCGAGTACAACTAGTATTATACCTAGGACTGAGC
dkgB-Up-F	GAAGGAGCATACTGAAGTATGTGACTGAC
dkgB-Up-R	GGAGGAGCTGTCAGAGAGGCGTGATTATGGGCTTCAGACTGTTGAG G
dkgB-Down-F	GCCTCTCTGACAGCTCCTCC
dkgB-Down-R	GCGCTGGTACGTTAACGGATTC
yeaE-Up-F	CTGTCTTATGCTCACTTTACCCAGTACG
yeaE-Up-R	TGTTTCCTCCCAGGTATTGCAC
yeaE-Down-F	GTGCAATACCTGGGAGGAAACATGTTGTGTGCCGGATGCATC
yeaE-Down-R	AAAACTTGTGAAATCGATCTTGAATCACATGG
yahK-Up-F	GCAGGATGACGCCAGCTTTG

yahK-Up-R	GGCAACGCCTGATATGTCATCG
yahK-Down-F	CGATGACATATCAGGCGTTGCCCCCTGTGGTTTAACATATTAACTTCGCTCTC
yahK-Down-R	GTGAGTGCGTTATCGTCTGACCAC
yjgB-Up-F	TTGGCTGCTCTGACTTTGATATCTG
yjgB-Up-R	TTTTTGGTCCTTCTCTGGTGTTGTTTG
yjgB-Down-F	CAAACAACACCAGAGAAGGACCAAAAAAAATCATTCGCAGCGCTGATC
yjgB-Down-R	TTGCCGCGTTATTATCTGCTAAATTTATTGATG
yqhC/yqhD/dkgA-Up-F	ATTTTCTGCCTACGATTGCGATTCTG
yqhC/yqhD/dkgA-Up-R	TACTTGCTCCCTTTGCTGGGC
yqhC/yqhD/dkgA-Down-F	GCCCAGCAAAGGGAGCAAGTAGCTTTTTACGCCTCAAACTTTCGTTTTC
yqhC/yqhD/dkgA-Down-R	CGAAAGCGCACAACGGCATC

Supplementary Table 6. The HPLC chromatographic conditions for the preparation of different final products.

Substrate	Column	Mobile phase	Test conditions
(*S*)-**1b**	ZORBAX Eclipse XDB-C18	Linear gradient 25%-50% acetonitrile (0.1% TFA) over 20 min	UV 280 nm, 30 °C, 3 mL min^{-1}
(*S*)-**1c** mixture	ZORBAX Eclipse XDB-C18	Linear gradient 30%-35% acetonitrile (0.1% TFA) over 20 min	UV 280 nm, 25 °C, 1 mL min^{-1}
(*S*)-**1d**	Ultimate XB-phenyl	40%: 60% methanol (0.1% TFA): ddH_2O (0.1% TFA)	UV 280 nm, 30 °C, 3 mL min^{-1}
(*S*)-**2d**	Ultimate XB-phenyl	30%: 70% acetonitrile (0.1% TFA): ddH_2O (0.1% TFA)	UV 280 nm, 30 °C, 3 mL min^{-1}
(*S*)-**3d**	Ultimate XB-phenyl	35%: 65% acetonitrile (0.1% TFA): ddH_2O (0.1% TFA)	UV 280 nm, 30 °C, 3 mL min^{-1}
(*S*)-**4d**	Ultimate XB-phenyl	35%: 65% acetonitrile (0.1% TFA): ddH_2O (0.1% TFA)	UV 280 nm, 30 °C, 3 mL min^{-1}
(*S*)-**5d**	Ultimate XB-phenyl	40%: 60% acetonitrile (0.1% TFA): ddH_2O (0.1% TFA)	UV 280 nm, 30 °C, 3 mL min^{-1}
(*S*)-**6d**	Ultimate XB-phenyl	27%: 73% acetonitrile (0.1% TFA): ddH_2O (0.1% TFA)	UV 280 nm, 30 °C, 3 mL min^{-1}
(*S*)-**7d**	Ultimate XB-phenyl	30%: 70% acetonitrile (0.1% TFA): ddH_2O (0.1% TFA)	UV 280 nm, 30 °C, 3 mL min^{-1}
(*S*)-**8d**	Ultimate XB-phenyl	27%: 73% acetonitrile (0.1% TFA): ddH_2O (0.1% TFA)	UV 280 nm, 30 °C, 3 mL min^{-1}
(*S*)-**9d**	Ultimate XB-phenyl	27%: 73% acetonitrile (0.1% TFA): ddH_2O (0.1% TFA)	UV 280 nm, 30 °C, 3 mL min^{-1}
(*S*)-**10d**	Ultimate XB-phenyl	28%: 72% acetonitrile (0.1% TFA): ddH_2O (0.1% TFA)	UV 280 nm, 30 °C, 3 mL min^{-1}
(*S*)-**11d**	Ultimate XB-phenyl	20%: 80% acetonitrile (0.1% TFA): ddH_2O (0.1% TFA)	UV 280 nm, 30 °C, 3 mL min^{-1}
(*S*)-**12b**	ZORBAX Eclipse XDB-C18	Linear gradient 26%-33% acetonitrile (0.1% TFA) over 20 min	UV 280 nm, 30 °C, 3 mL min^{-1}
(*S*)-**12c**	ZORBAX Eclipse XDB-C18	Linear gradient 35%-57% methanol (0.1% TFA) over 25 min	UV 280 nm, 30 °C, 3 mL min^{-1}
(*S*)-**12d**	Ultimate XB-phenyl	37%: 63% methanol (0.1% TFA):ddH_2O (0.1% TFA)	UV 280 nm, 30 °C, 3 mL min^{-1}

Supplementary Figures

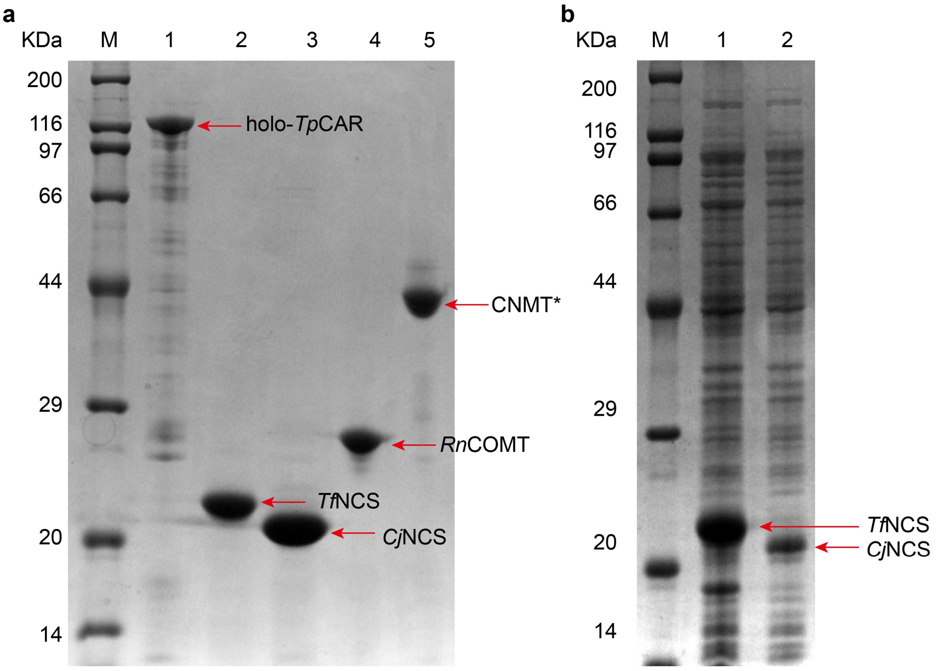

Supplementary Figure 1. SDS-PAGE analysis of purified proteins and protein expression in *E. coli* BL21 (*DE3*). **a** Purified enzymes used for the artificial multi-enzyme cascade *in vitro*. M: marker; lane 1: holo-*Tp*CAR; lane 2: *Tf*NCS; lane 3: *Cj*NCS; lane 4: *Rn*COMT; lane 5: CNMT$^{N92A/F332A}$. **b** Expression of *Tf*NCS and *Cj*NCS in *E. coli* BL21 (*DE3*). M: marker; lane 1: the supernatant of cell lysates expressing *Tf*NCS induced by IPTG; lane 2: the supernatant of cell lysates expressing *Cj*NCS induced by IPTG. Source data are provided as a Source Data file.

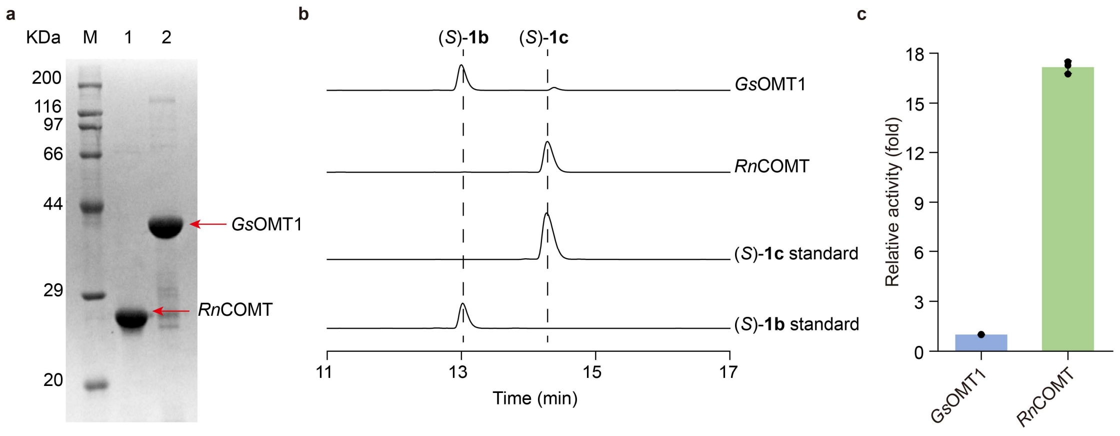

Supplementary Figure 2. Screening of *O*-methyltransferases with high catalytic activity towards (*S*)-**1b**. **a** Purfied *Rn*COMT and *Gs*OMT1 from *E. coli* BL21 (*DE3*). M: marker; lane 1: purified *Rn*COMT; lane 2: purified *Gs*OMT1. **b** HPLC analysis of (*S*)-**1b** and (*S*)-**1c** in the reaction systems.

c Relative activity of *Gs*OMT1 and *Rn*COMT towards (*S*)-**1b**. The relative activity was measured by the ratio of (*S*)-**1b** consumption by *Gs*OMT1 to that of *Rn*COMT. All data is presented as mean value of three independent experiments and the error bars indicate ± sd. Source data are provided as a Source Data file.

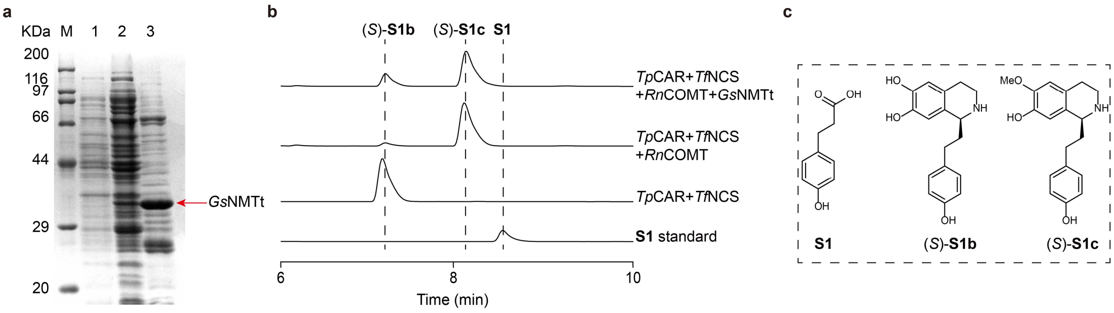

Supplementary Figure 3. Characterization of the *N*-methylation activity of *Gs*NMTt towards (*S*)-**S1c**. **a** SDS-PAGE analysis of *Gs*NMTt expressed in *E. coli* BL21 (*DE3*). M: marker; lane 1: no IPTG induction; lane 2: the supernatant of cell lysates expressing *Gs*NMTt induced by IPTG; lane 3: purified *Gs*NMTt. **b** HPLC analysis of **S1**, (*S*)-**S1b** and (*S*)-**S1c** in a cascade reaction containing the PEIA moiety module, *Rn*COMT and *Gs*NMTt in a one-pot two-step process *in vitro*. **c** The chemical structure of **S1**, (*S*)-**S1b** and (*S*)-**S1c**. Source data are provided as a Source Data file.

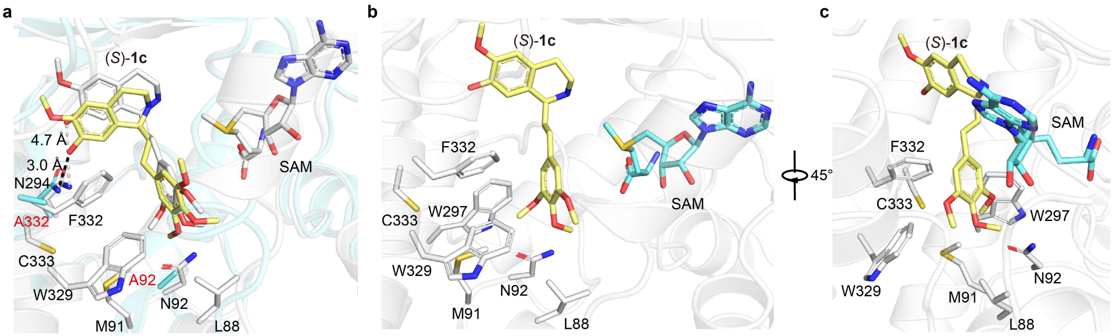

Supplementary Figure 4. Molecular docking analysis of (*S*)-**1c** with CNMT^{WT} and CNMT$^{N92A/F332A}$. **a** Comparison of the (*S*)-**1c** binding site in CNMT^{WT} and CNMT$^{N92A/F332A}$. CNMT^{WT} is colored by white while CNMT$^{N92A/F332A}$ is colored by cyan. (*S*)-**1c** in CNMT$^{N92A/F332A}$ is colored by yellow. Residues in CNMT^{WT} located around trimethoxyphenyl group of (*S*)-**1c** within 4.0 Å are indicated by white sticks. The mutated residues in CNMT$^{N92A/F332A}$ were indicated by cyan sticks. The dash line indicates the presence of a hydrogen bond. **b** Substrate (*S*)-**1c**, SAM and the

selected residues with potential interactions with (*S*)-**1c** colored by yellow, cyan and white, respectively. Oxygen, nitrogen and sulfur atom are indicated by red, blue and yellow colors, respectively. **c** Close-up views of a 45° rotation along the Y-axis of (a).

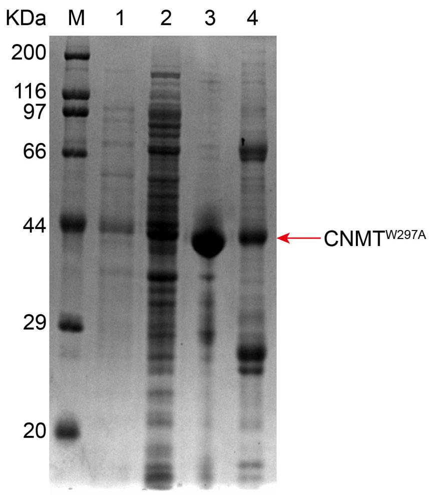

Supplementary Figure 5. SDS-PAGE analysis of CNMT^{W297A} expressed in *E. coli* BL21 (*DE3*). M: marker; lane 1: no IPTG induction; lane 2: the supernatant of cell lysates expressing CNMT^{W297A} induced by IPTG; lane 3: the pellet; lane 4: purified CNMT^{W297A}. Source data are provided as a Source Data file.

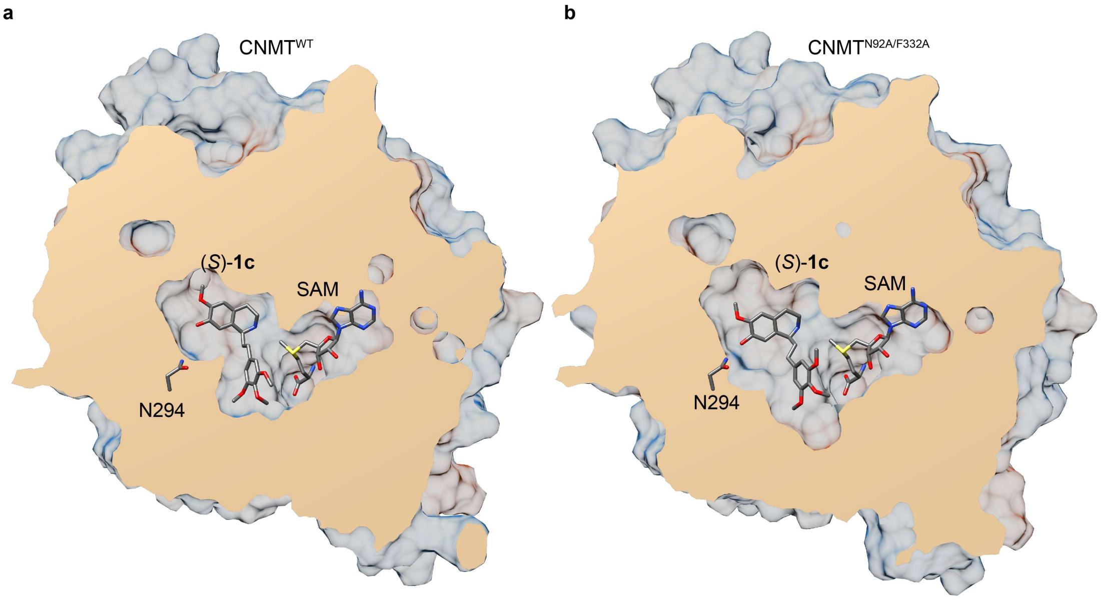

Supplementary Figure 6. Cross-section of the substrate binding pocket. **a** The substrate binding site highlighted in cross-section of CNMT^{WT}. **b** The substrate binding site highlighted in cross-section of CNMT$^{N92A/F332A}$.

a

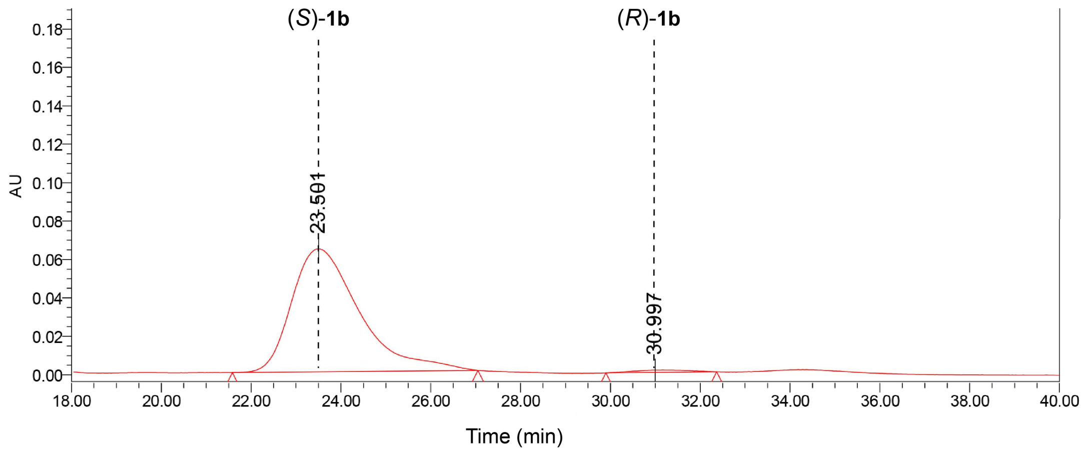

Name	Retention time (min)	Area (µV sec)	Height (µV)	% Area
1	23.501	6672624	63969	98.50
2	30.997	101869	1187	1.50

b

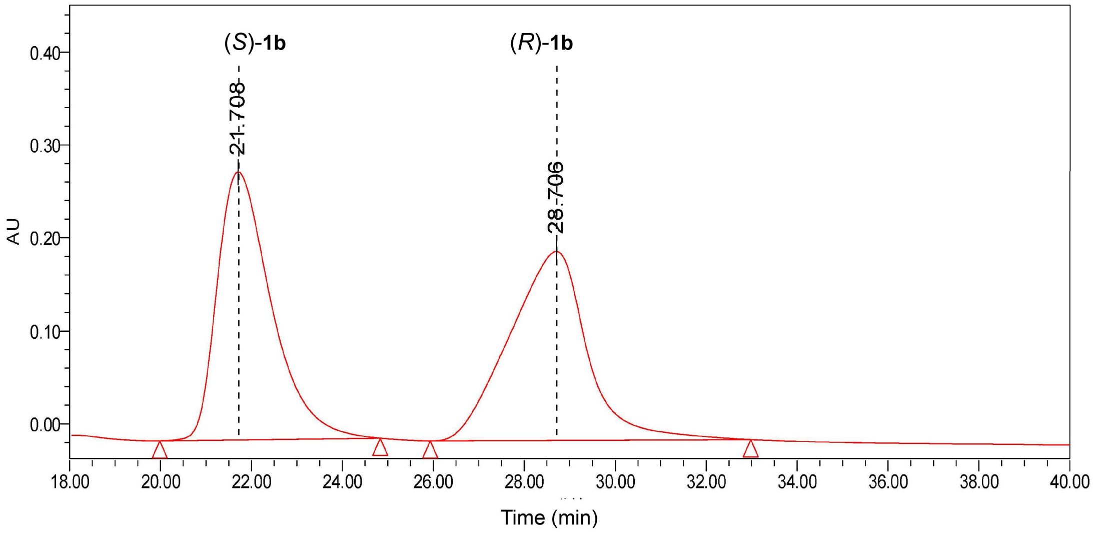

Name	Retention time (min)	Area (µV sec)	Height (µV)	% Area
1	21.708	23827994	287725	49.69
2	28.706	24125144	203126	50.31

Supplementary Figure 7. Chiral HPLC analysis of (*S*)-**1b** and (*rac*)-**1b** synthesized from substrate **1** and dopamine *in vitro*. **a** Chiral HPLC analysis of (*S*)-**1b** synthesized by *Tp*CAR and *Tf*NCS in HEPES buffer. **b** Chiral HPLC analysis of (*rac*)-**1b** synthesized by *Tp*CAR in KPI buffer. The enantioselectivities were analyzed via HPLC on a chiral stationary phase.

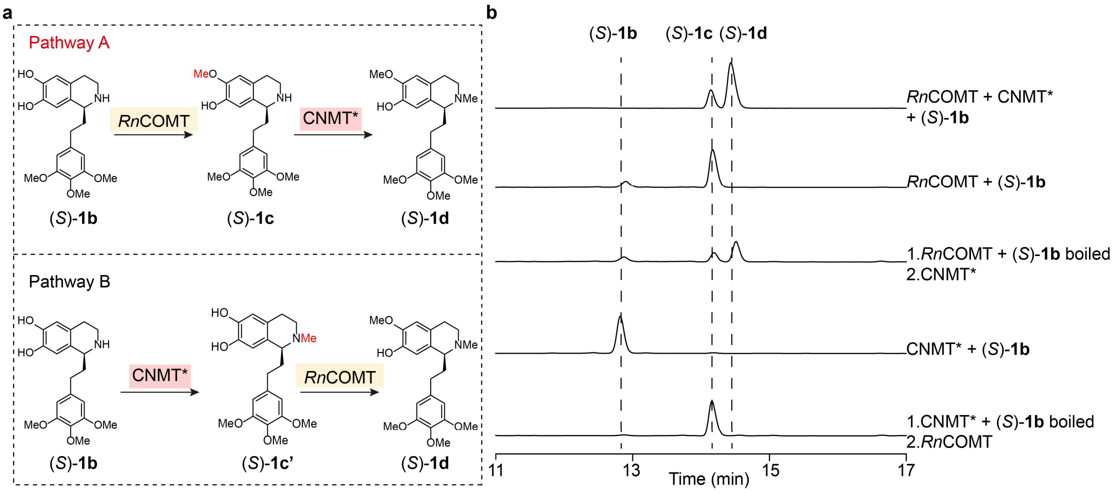

Supplementary Figure 8. Reaction order of *Rn*COMT and CNMT* towards (*S*)-**1b**. **a** Two proposed reaction pathways from (*S*)-**1b** to (*S*)-**1d**. Pathway A: (*S*)-**1b** was first *O*-methylated by *Rn*COMT and then *N*-methylated by CNMT*. Pathway B: (*S*)-**1b** was first *N*-methylated by CNMT* and then *O*-methylated by *Rn*COMT. **b** HPLC analysis of (*S*)-**1b**, (*S*)-**1c** and (*S*)-**1d** in cascade reaction. Method for reaction pathway A: 25 μM CNMT*, 2 mM (*S*)-**1b** and 4 mM SAM were incubated in a 200 μL reaction system for 2 h at 30 °C. Next, the above reaction solution was boiled at 95 °C for 5 min and centrifuged at 20000 *g* for 5 min. Then, 5 μM *Rn*COMT was added into the above supernatant to initiate the reaction. Method for reaction pathway B: 5 μM *Rn*COMT, 2 mM (*S*)-**1b** and 4 mM SAM were incubated in a 200 μL reaction system for 2 h at 30 °C. Next, the above reaction solution was boiled at 95 °C for 5 min and centrifuged at 20000 *g* for 5 min. Then, 25 μM CNMT* was added into the above supernatant to initiate the reaction. Source data are provided as a Source Data file.

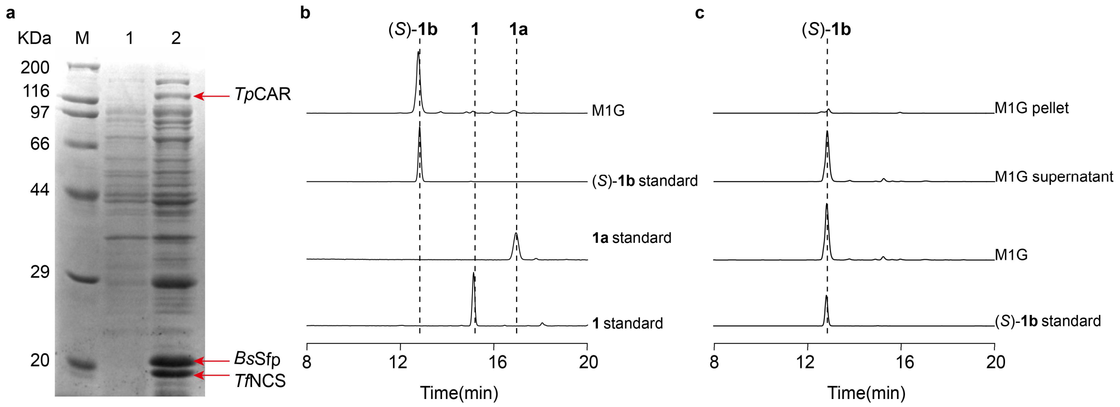

Supplementary Figure 9. Functional verification of the PEIA moiety module in engineered strain IAA (M1G). **a** SDS-PAGE analysis of enzymes of the PEIA moiety module expressed in IAA. M:

marker; lane 1: no IPTG induction; lane 2: the supernatant of cell lysates expressing the PEIA moiety module in strain M1G induced by IPTG. **b** HPLC analysis of (*S*)-**1b** produced from 5 mM substrate **1** and 5 mM dopamine by strain M1G. **c** Distribution analysis of (*S*)-**1b** in the supernatant and the pellet of strain M1G. Source data are provided as a Source Data file.

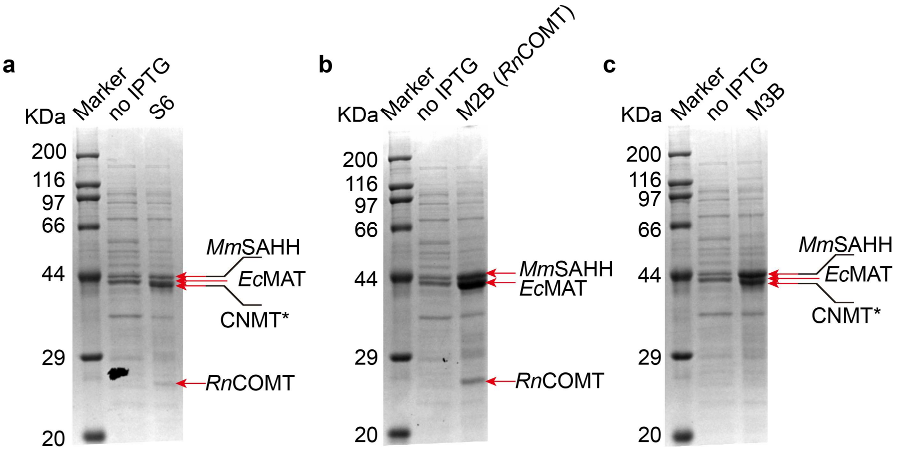

Supplementary Figure 10. SDS-PAGE analysis of protein expression in strain S6, M2B (*Rn*COMT) and M3B. **a** SDS-PAGE analysis of *Mm*SAHH, *Ec*MAT, CNMT* and *Rn*COMT expressed in strain S6. **b** SDS-PAGE analysis of *Mm*SAHH, *Ec*MAT and *Rn*COMT expressed in strain M2B (*Rn*COMT). **c** SDS-PAGE analysis of *Mm*SAHH, *Ec*MAT and CNMT* expressed in strain M3B. Source data are provided as a Source Data file.

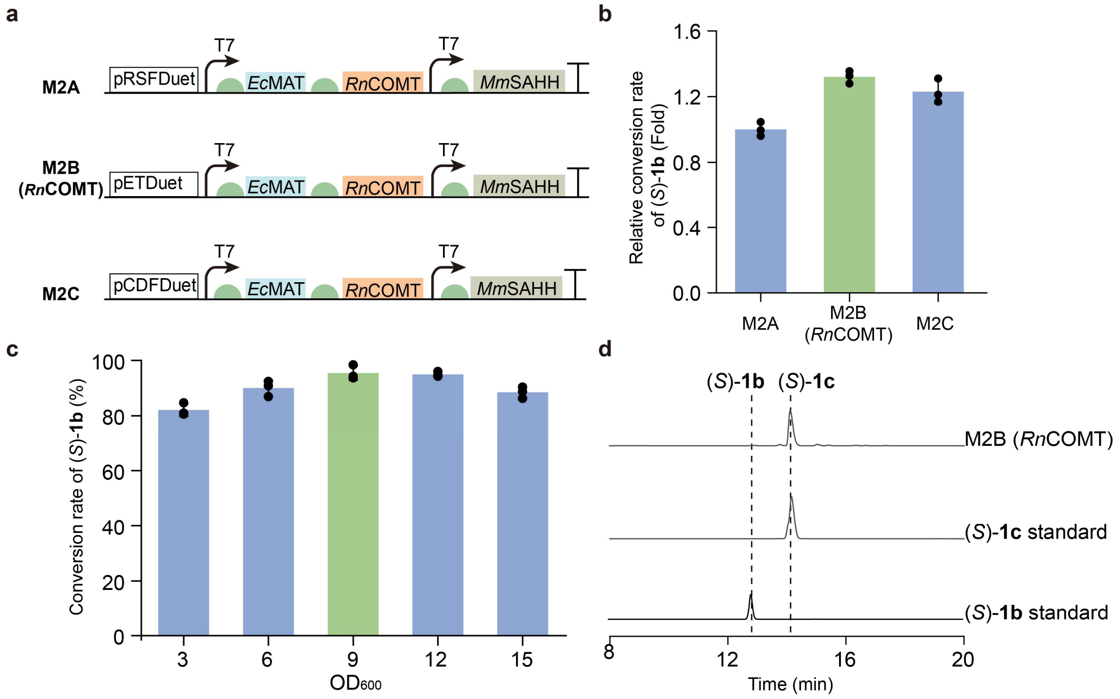

Supplementary Figure 11. Biosynthesis of (*S*)-**1c** in strain IAA containing the OMT module. **a** Construction of three engineered strains using different plasmids to express *Rn*COMT, *Ec*MAT and *Mm*SAHH. **b** Comparison of the biotransformation efficiency of (*S*)-**1b** using M2A, M2B (*Rn*COMT) and M2C. The conversion rate of (*S*)-**1b** in strain M2A is set as 1. **c** Optimization of the concentration of the strain M2B (*Rn*COMT) to improve the conversion rate of (*S*)-**1b**. **d** HPLC analysis of (*S*)-**1c** synthesized by strain M2B (*Rn*COMT). All data is presented as mean value of three independent experiments and the error bars indicate ± sd. Source data are provided as a Source Data file.

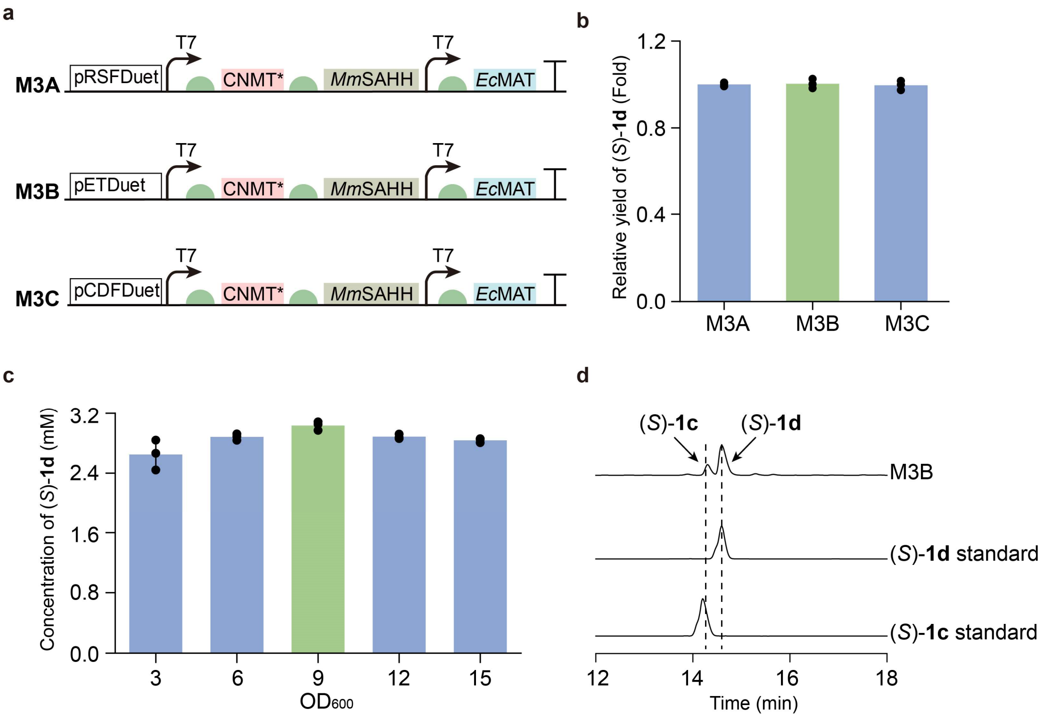

Supplementary Figure 12. Biosynthesis of (*S*)-**1d** in strain IAA containing the NMT module. **a** Construction of three engineered strains using different plasmids to express CNMT*, *Ec*MAT and *Mm*SAHH. **b** Comparison of the efficiency of the biotransformation of (*S*)-**1c** to (*S*)-**1d** using M3A, M3B and M3C. The yield of (*S*)-**1d** in strain M3A is set as 1. **c** Optimization of the concentration of the strain M3B to improve the titer of (*S*)-**1d**. **d** HPLC analysis of (*S*)-**1d** synthesized by strain M3B. All data is presented as mean value of three independent experiments and the error bars indicate ± sd. Source data are provided as a Source Data file.

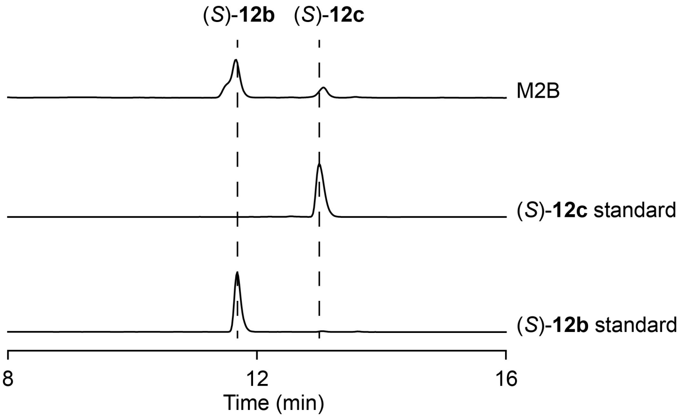

Supplementary Figure 13. HPLC analysis of (*S*)-**12b** and (*S*)-**12c** in reaction system of strain M2B (*Rn*COMT). Source data are provided as a Source Data file.

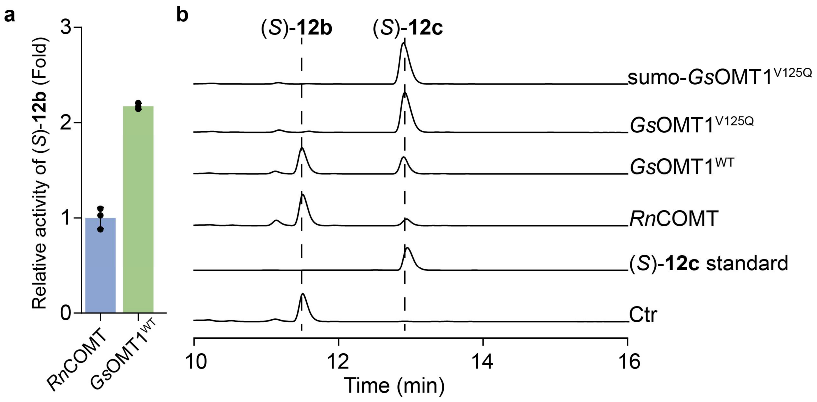

Supplementary Figure 14. Screening of *O*-methyltransferases with high catalytic activity towards (*S*)-**12b**. **a** Relative activity of *Rn*COMT and *Gs*OMT1^{WT} towards (*S*)-**12b**. The relative activity was measured by the ratio of the (*S*)-**12c** titer of *Gs*OMT1^{WT} to that of *Rn*COMT. Green column is the mutant with the highest catalytic activity. **b** HPLC analysis of the catalytic activity of *Rn*COMT, *Gs*OMT1^{WT}, *Gs*OMT1^{V125Q} and sumo-*Gs*OMT1^{V125Q} towards (*S*)-**12b** using 1.60 mM (*S*)-**12b**, 4 mM SAM and 5 µM enzymes. Ctr means the reaction system without enzymes. Source data are provided as a Source Data file.

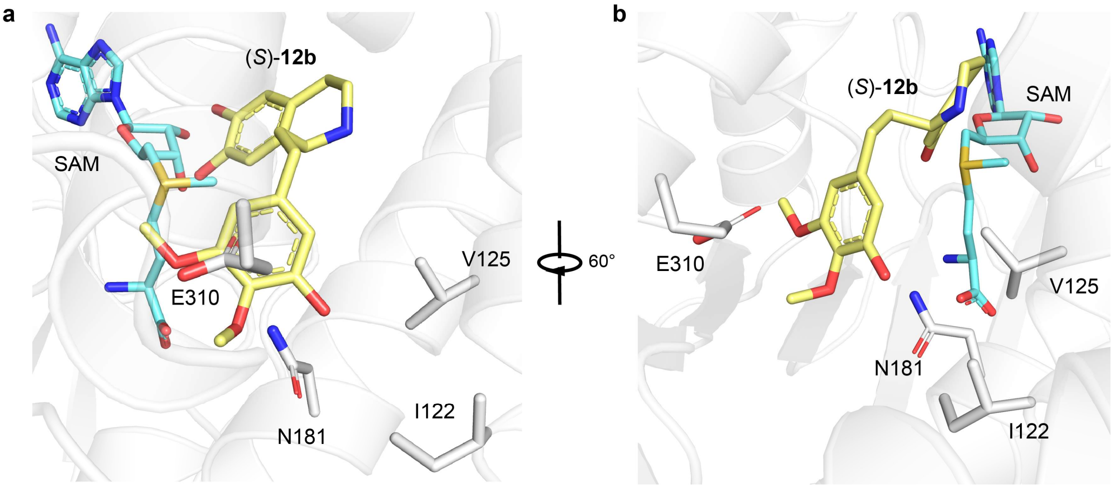

Supplementary Figure 15. Close-up views of the substrate binding pocket of *Gs*OMT1 for (*S*)-**12b**. (a) Substrate (*S*)-**12b**, SAM and the selected residues with potential interactions with (*S*)-**12b** are colored by yellow, cyan and white, respectively. Oxygen, nitrogen and sulfur atom are indicated by red, blue and yellow colors, respectively. (b) Close-up views of a 60° rotation along the Y-axis of (a).

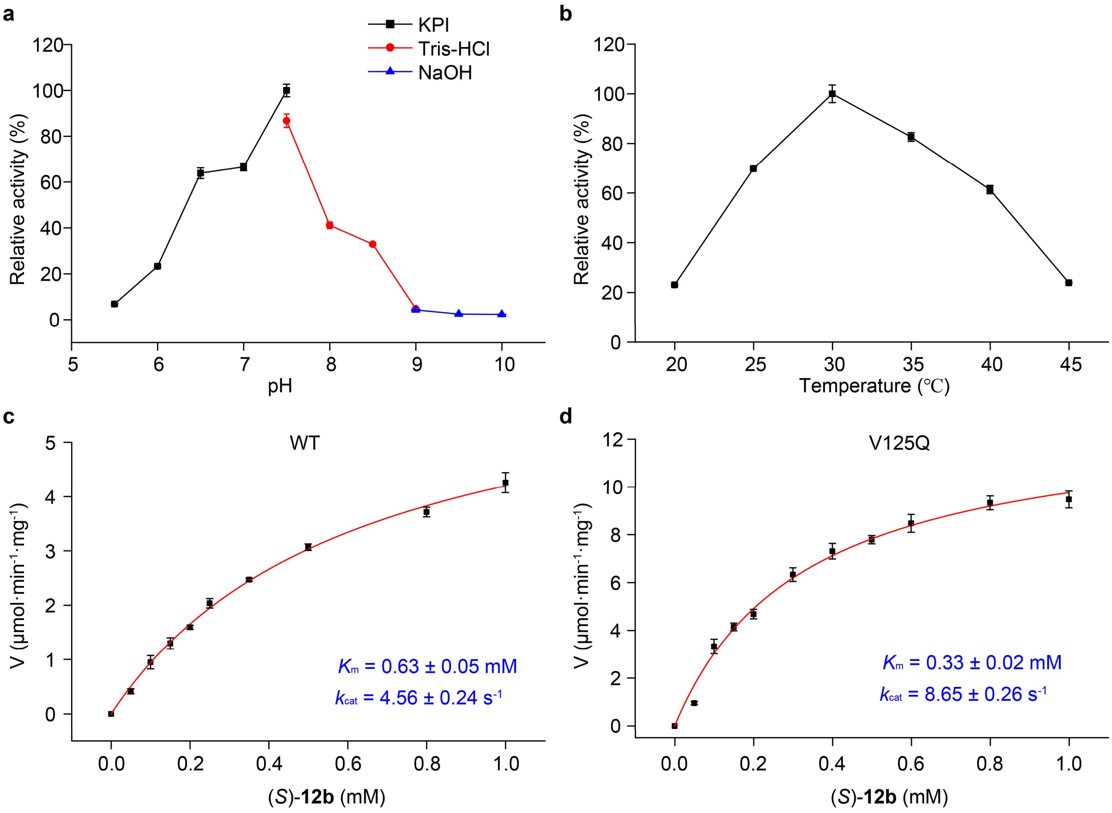

Supplementary Figure 16. The kinetic plots of *Gs*OMT1^{WT} and *Gs*OMT^{V125Q} towards substrate

(S)-**12b**. **a** Optimization of pH using potassium phosphate buffer (pH 5.5−7.5, 200 mM), Tris-HCl buffer (pH 7.5−9.0, 200 mM) and Glycine-NaOH (pH 9.0−10.0, 200 mM). **b** Optimization of temperature. **c** The kinetic plots of wild-type *Gs*OMT1^{WT}. **d** The kinetic plots of *Gs*OMT1^{V125Q}. All data is presented as mean value of three independent experiments and the error bars indicate ± sd. Source data are provided as a Source Data file.

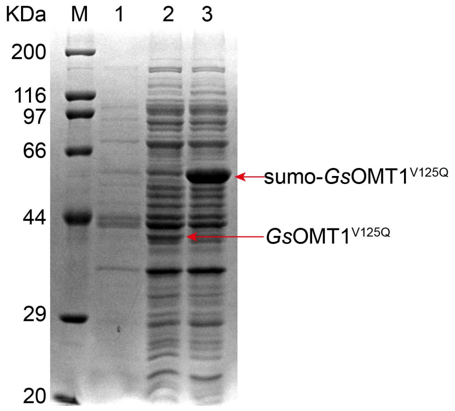

Supplementary Figure 17. SDS-PAGE analysis of *Gs*OMT1^{V125Q} and sumo-*Gs*OMT1^{V125Q} expressed in *E. coli* BL21 (*DE3*). M: Marker; lane 1: no IPTG induction; lane 2: the supernatant of cell lysates expressing *Gs*OMT1^{V125Q} induced by IPTG; lane 3: the supernatant of cell lysates expressing sumo-*Gs*OMT1^{V125Q} induced by IPTG. Source data are provided as a Source Data file.

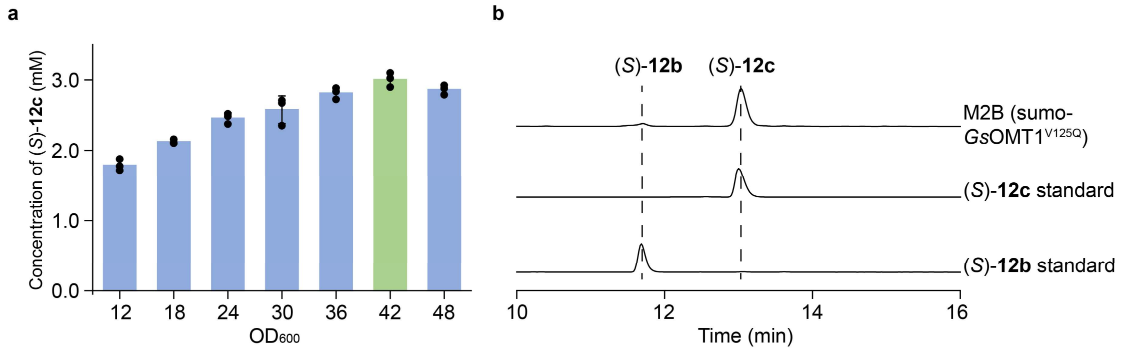

Supplementary Figure 18. Biosynthesis of (S)-**12c** in strain M2B (sumo-*Gs*OMT1^{V125Q}). **a** optimizing the OD_{600} of strain M2B (sumo-*Gs*OMT1^{V125Q}) to improve the titer of (S)-**12c**. **b** HPLC analysis of (S)-**12c** synthesized by strain M2B (sumo-*Gs*OMT1^{V125Q}). All data is presented as mean value of three independent experiments and the error bars indicate ± sd. Source data are provided as a Source Data file.

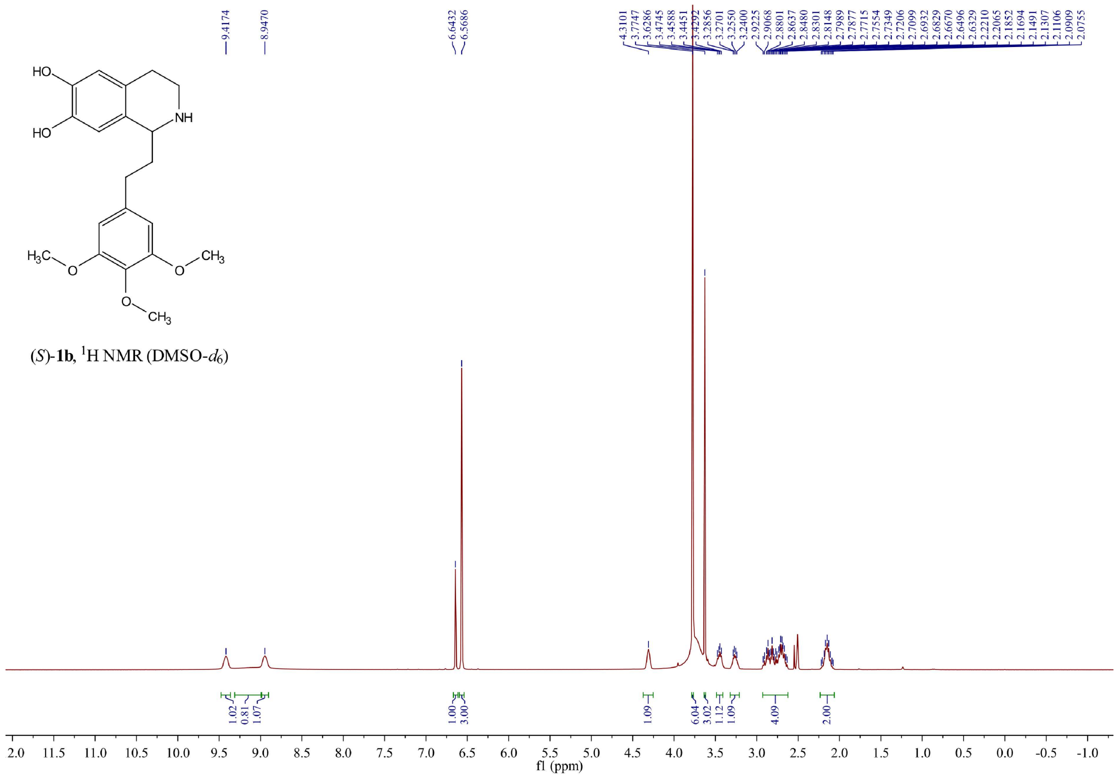

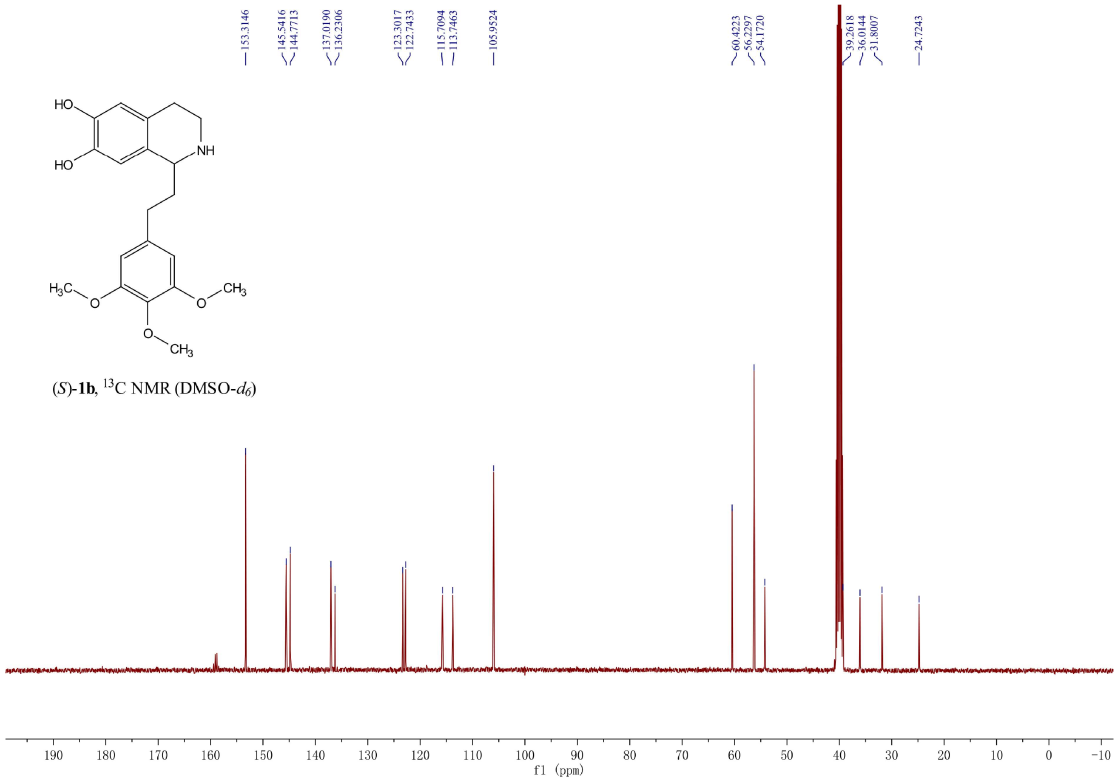

Supplementary Figure 19. ^{1}H NMR and ^{13}C NMR spectra of (*S*)-**1b**.

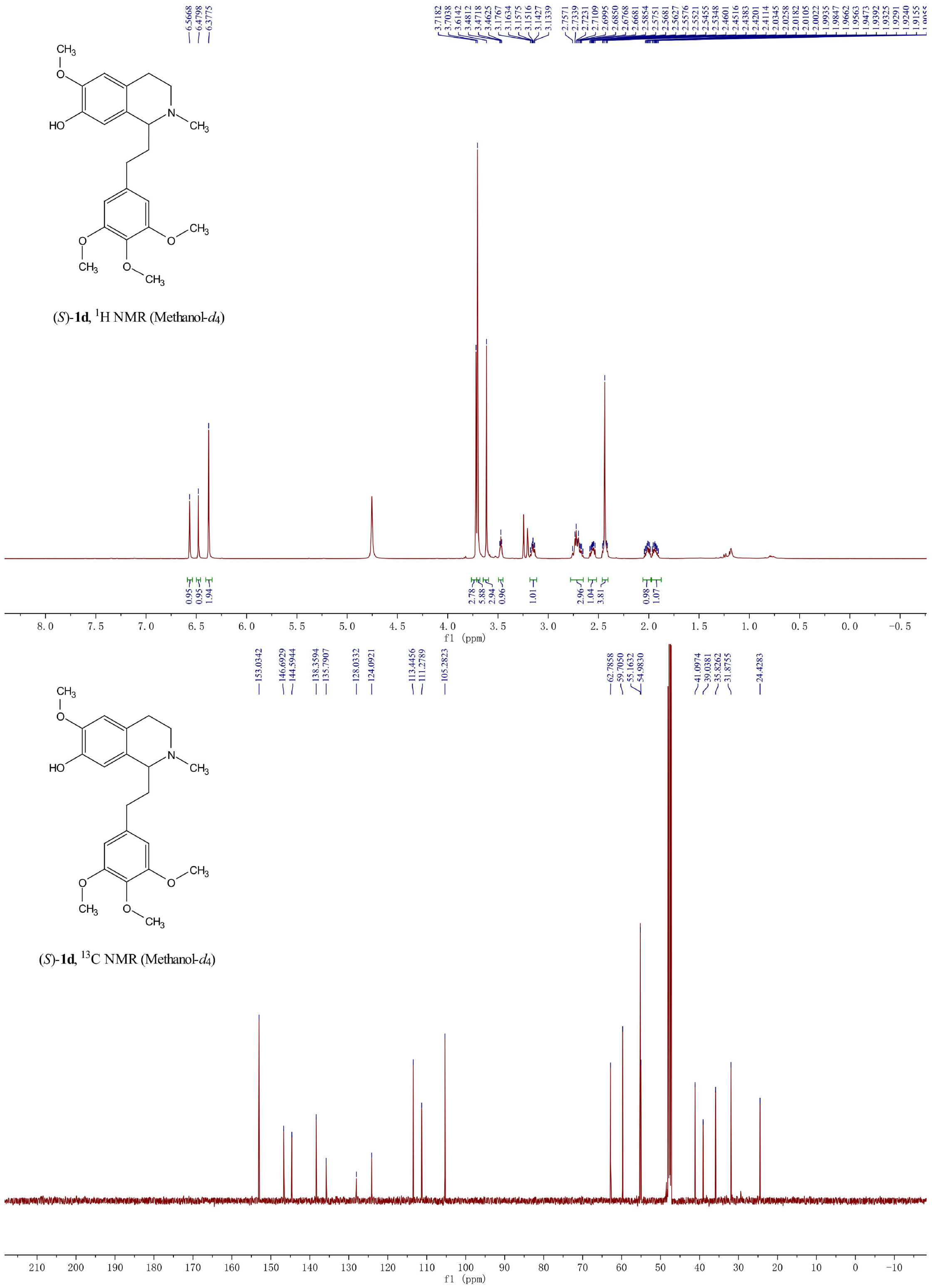

Supplementary Figure 20. ^{1}H NMR and ^{13}C NMR spectra of (*S*)-**1d**.

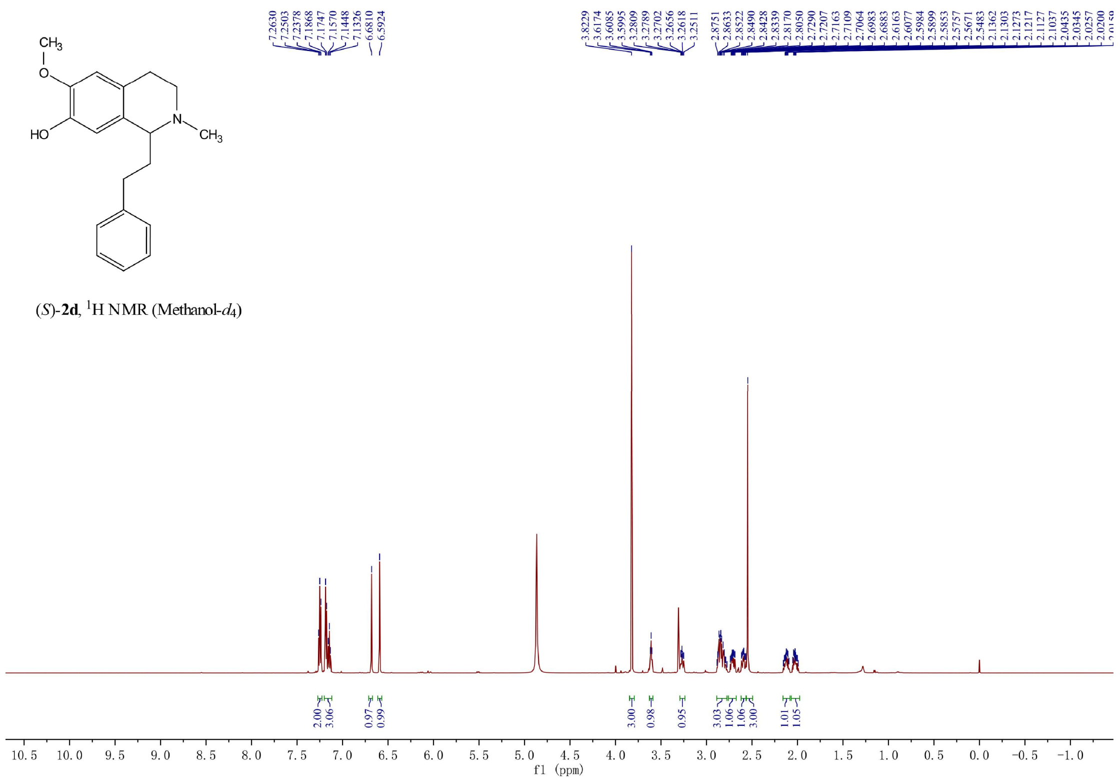

Supplementary Figure 21. ^{1}H NMR spectra of (*S*)-**2d**.

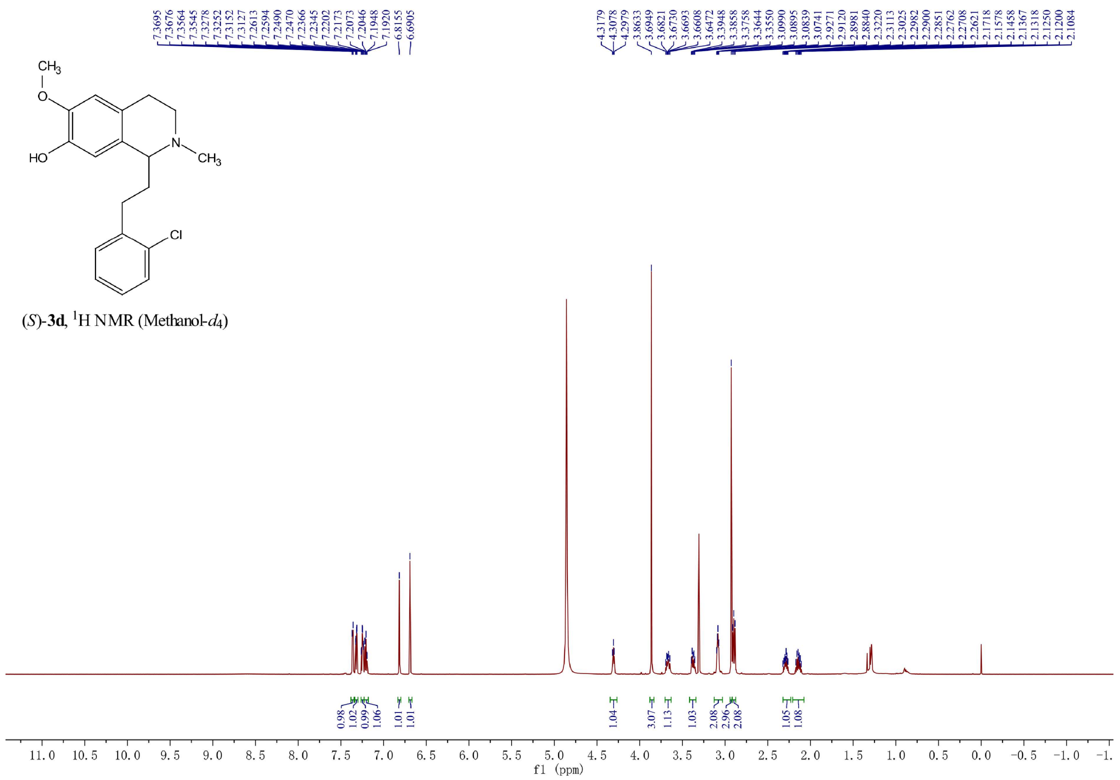

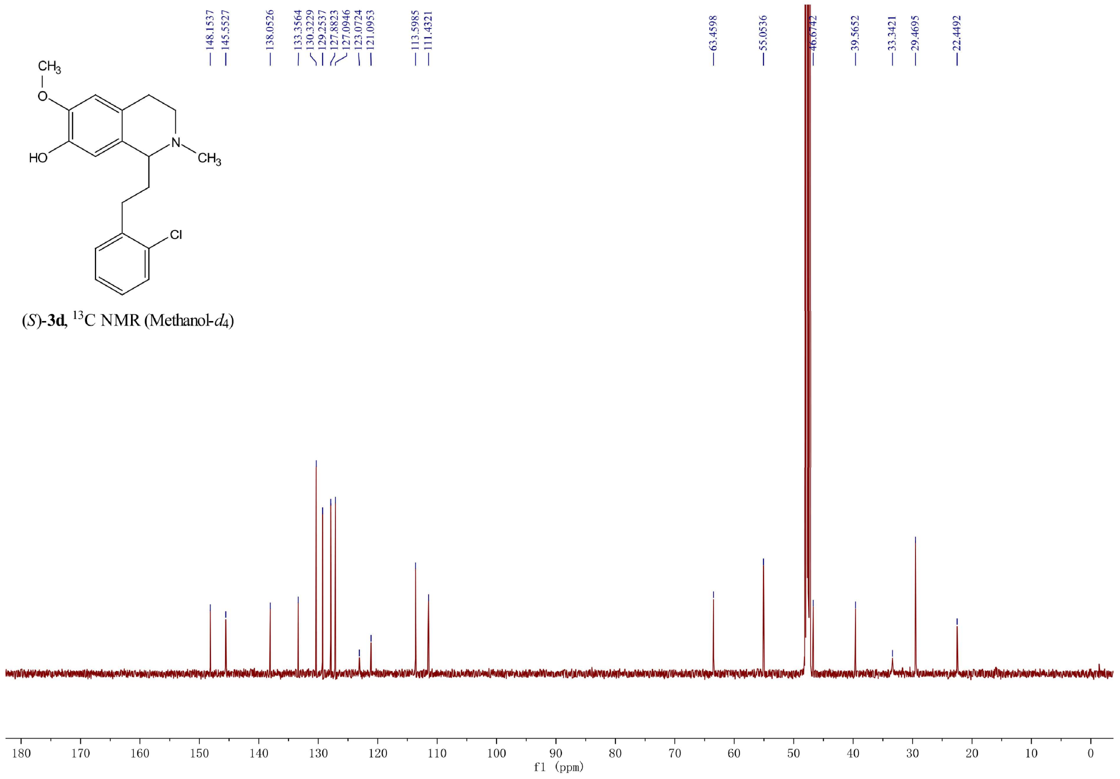

Supplementary Figure 22. ^{1}H NMR and ^{13}C NMR spectra of (*S*)-**3d**.

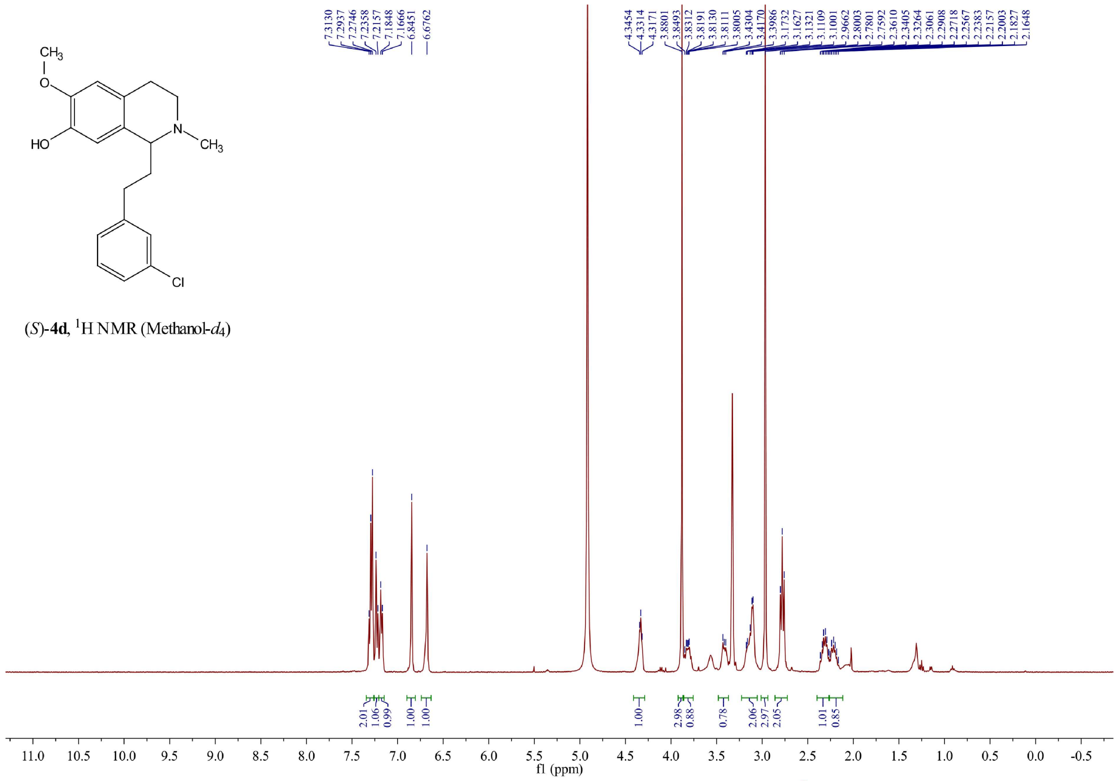

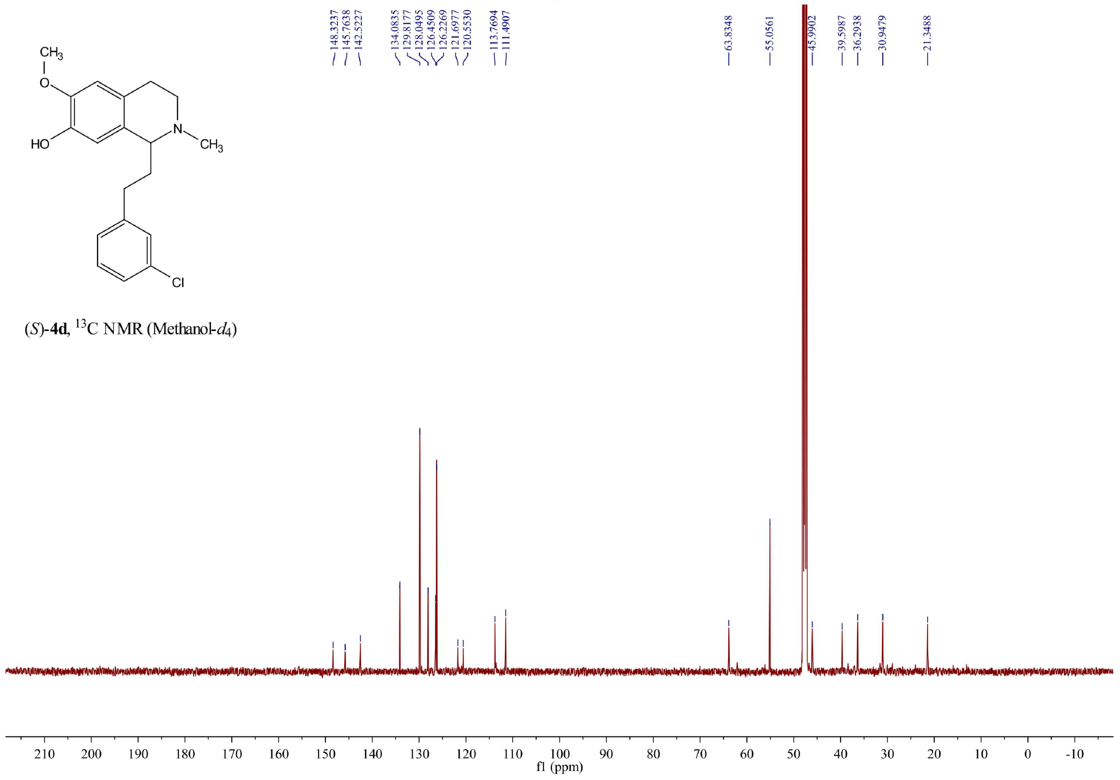

Supplementary Figure 23. ^{1}H NMR and ^{13}C NMR spectra of (*S*)-**4d**.

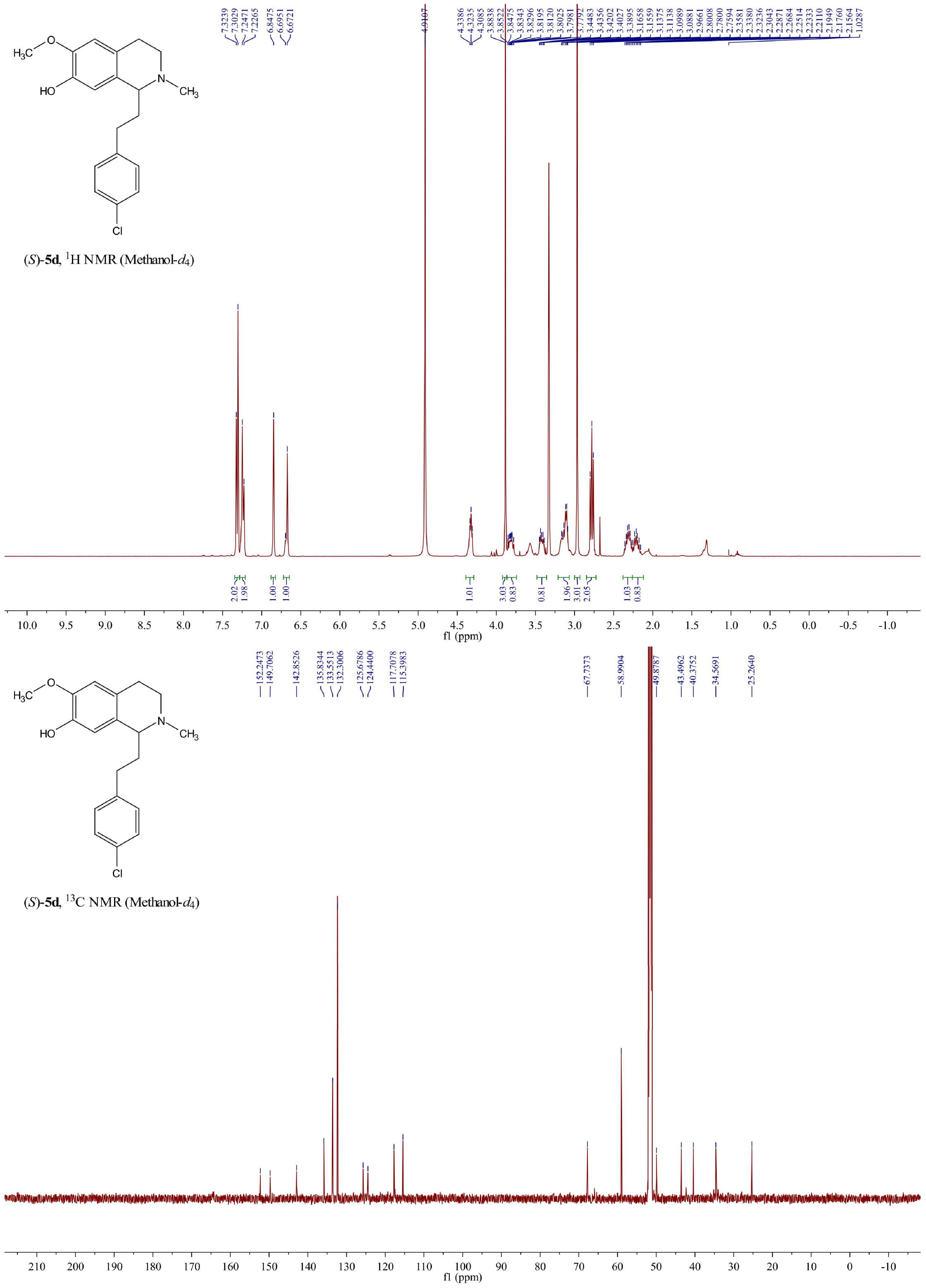

Supplementary Figure 24. ^{1}H NMR and ^{13}C NMR spectra of (*S*)-**5d**.

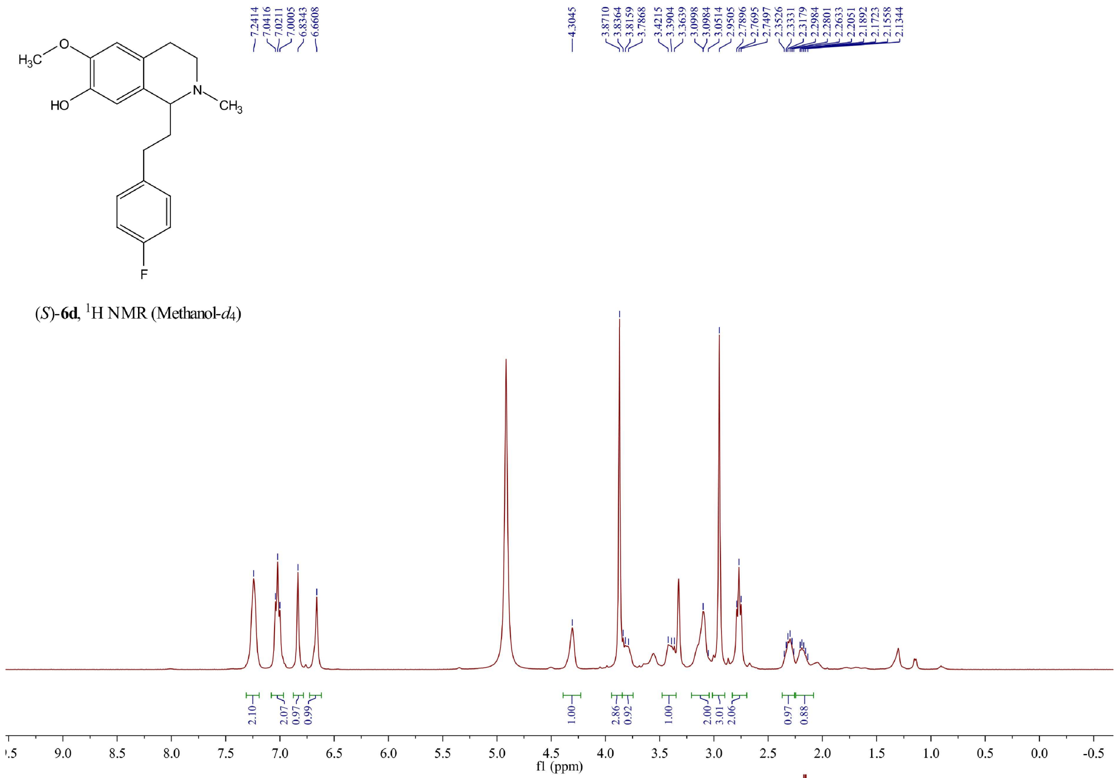

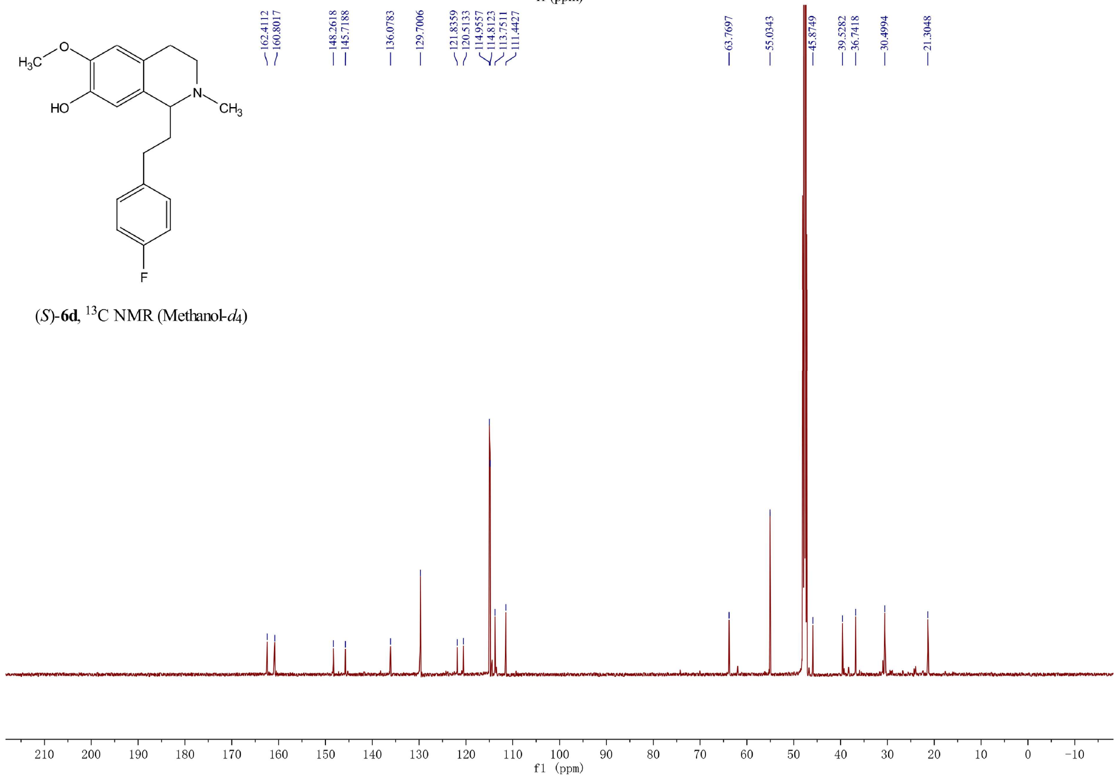

Supplementary Figure 25. ^{1}H NMR and ^{13}C NMR spectra of (*S*)-**6d**.

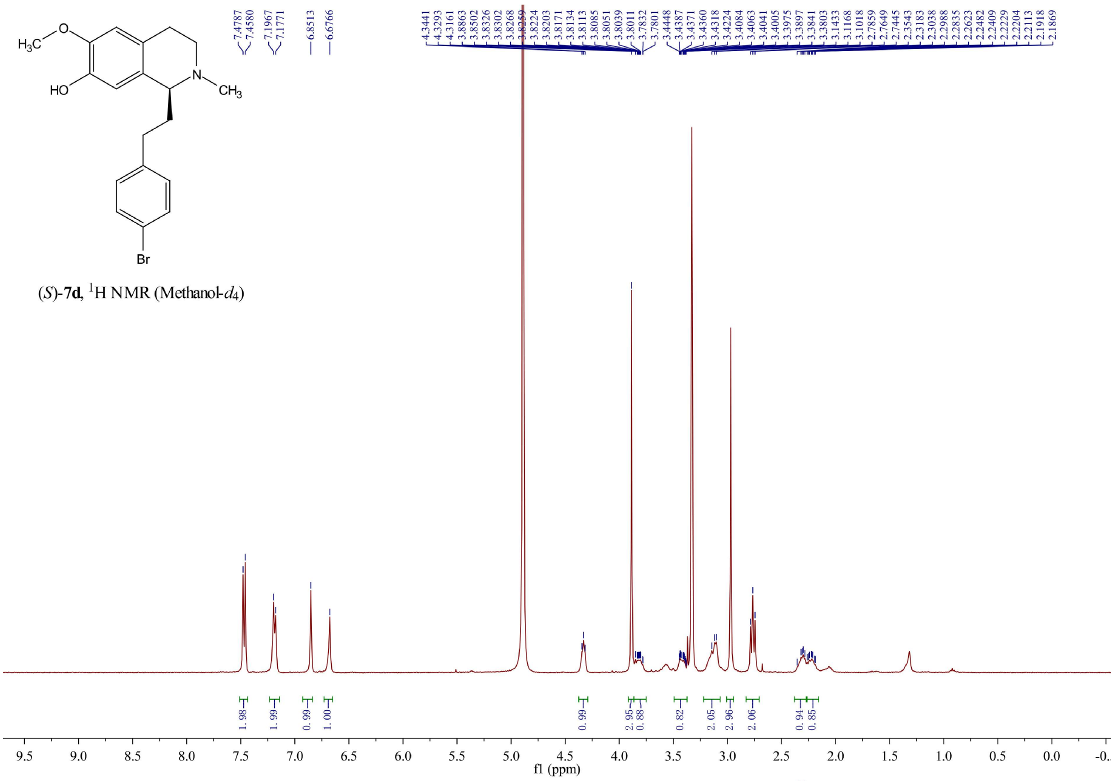

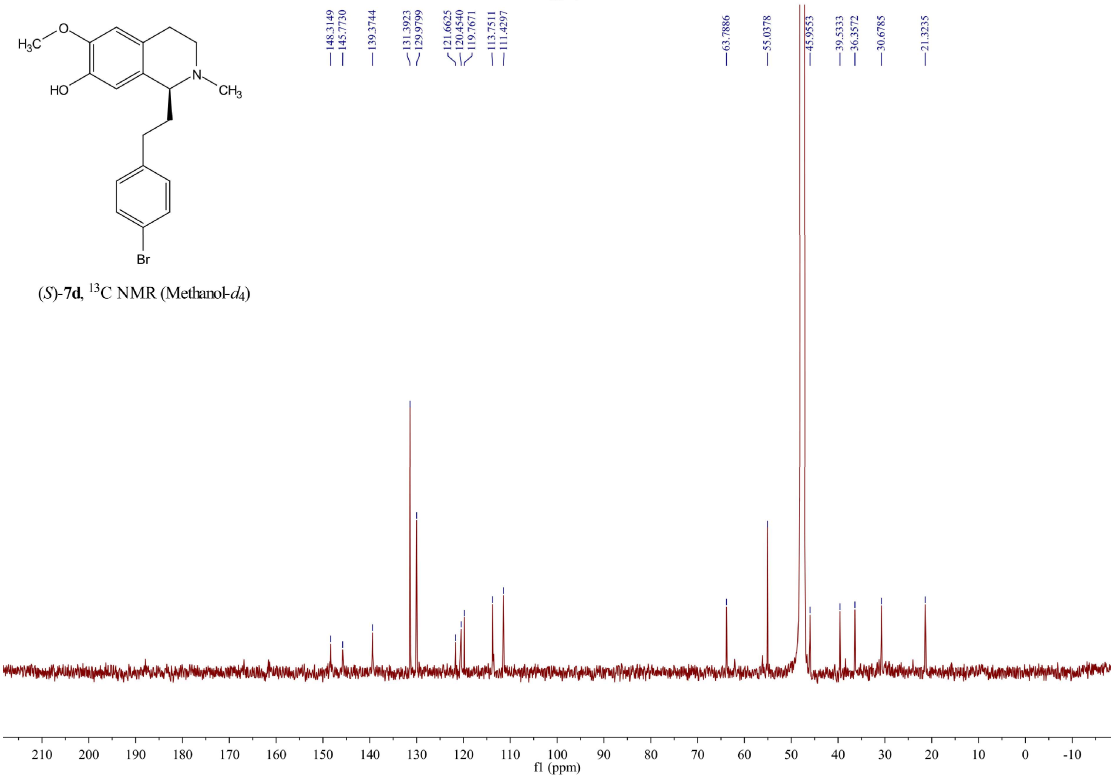

Supplementary Figure 26. ^{1}H NMR and ^{13}C NMR spectra of (*S*)-**7d**.

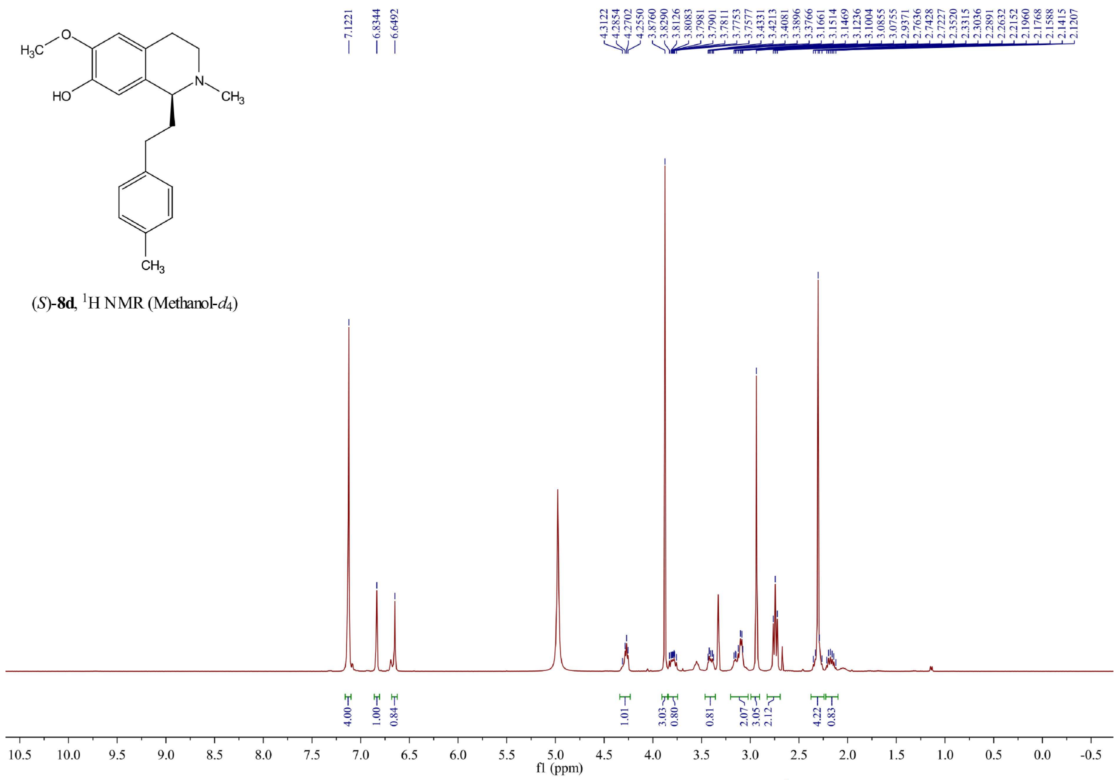

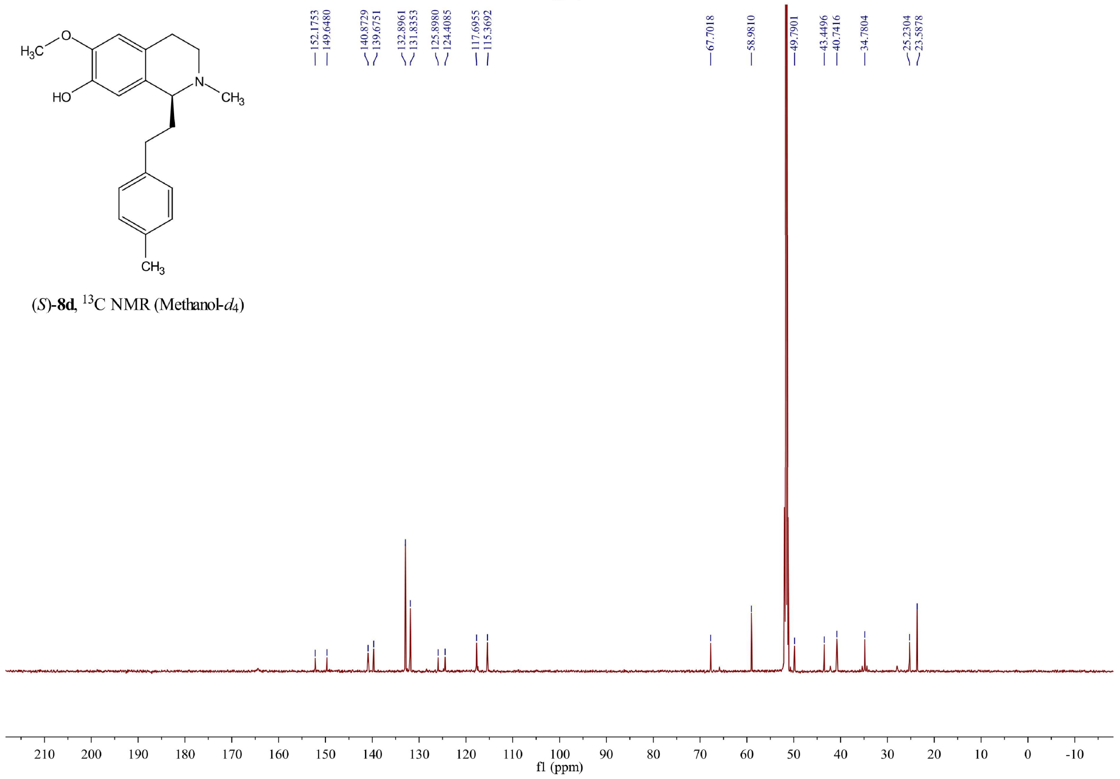

Supplementary Figure 27. ^{1}H NMR and ^{13}C NMR spectra of (*S*)-**8d**.

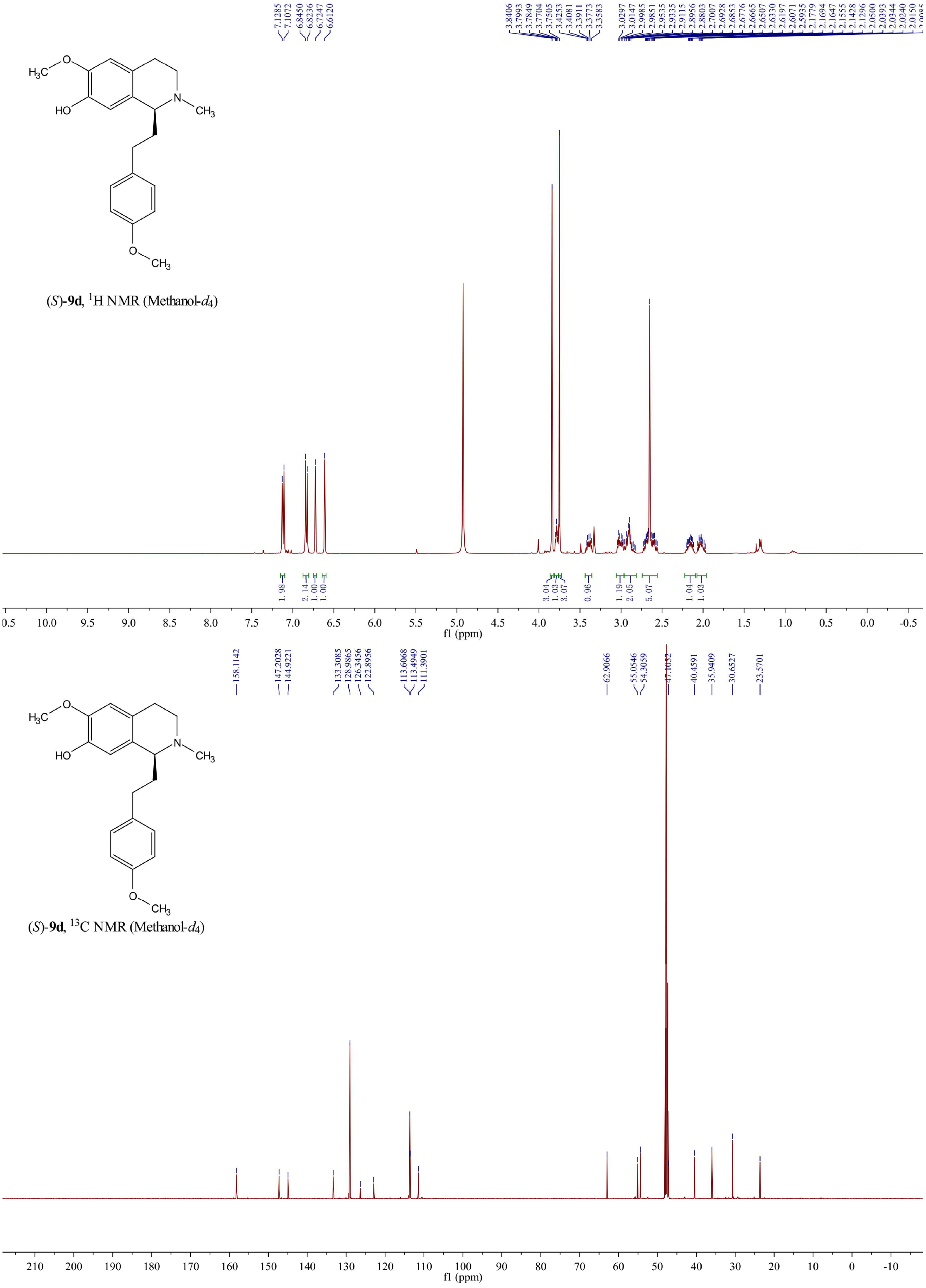

Supplementary Figure 28. ^{1}H NMR and ^{13}C NMR spectra of (*S*)-**9d**.

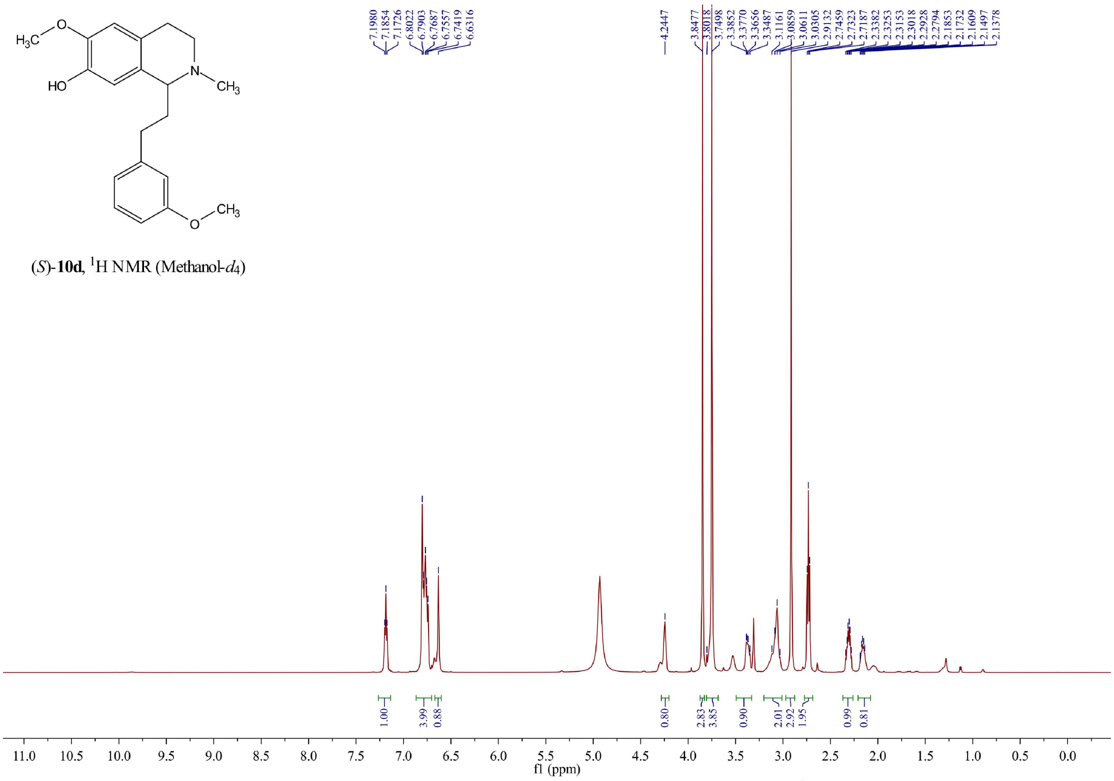

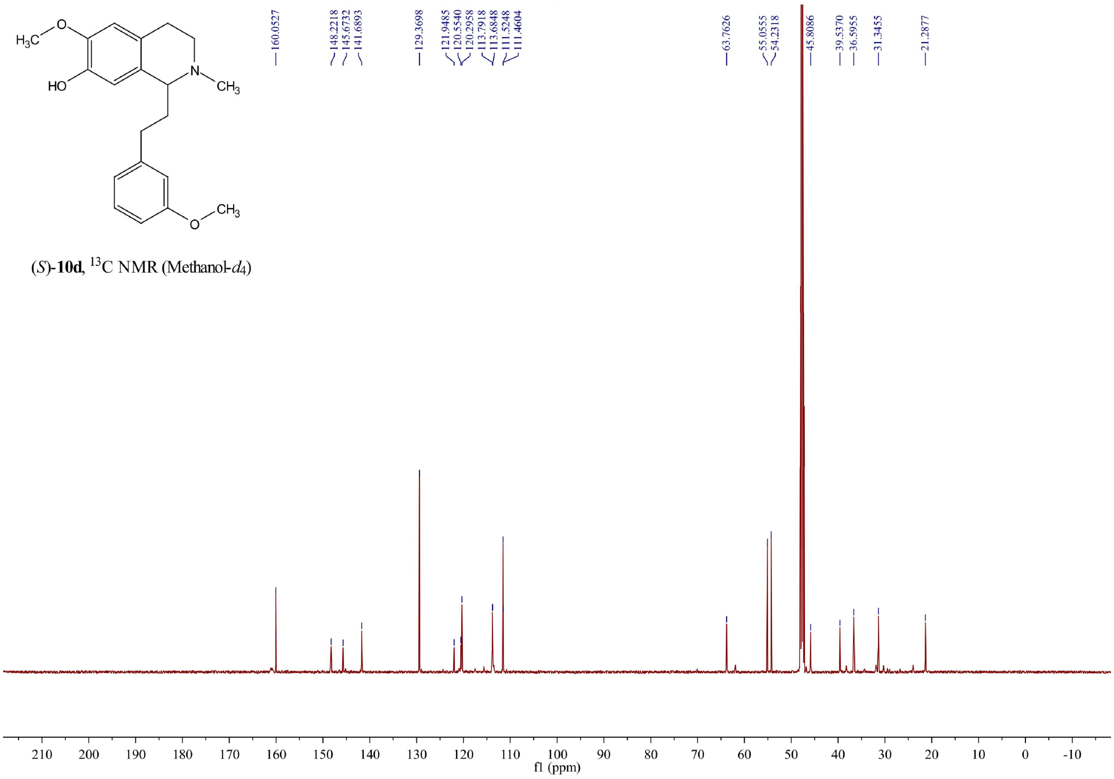

Supplementary Figure 29. ^{1}H NMR and ^{13}C NMR spectra of (*S*)-**10d**.

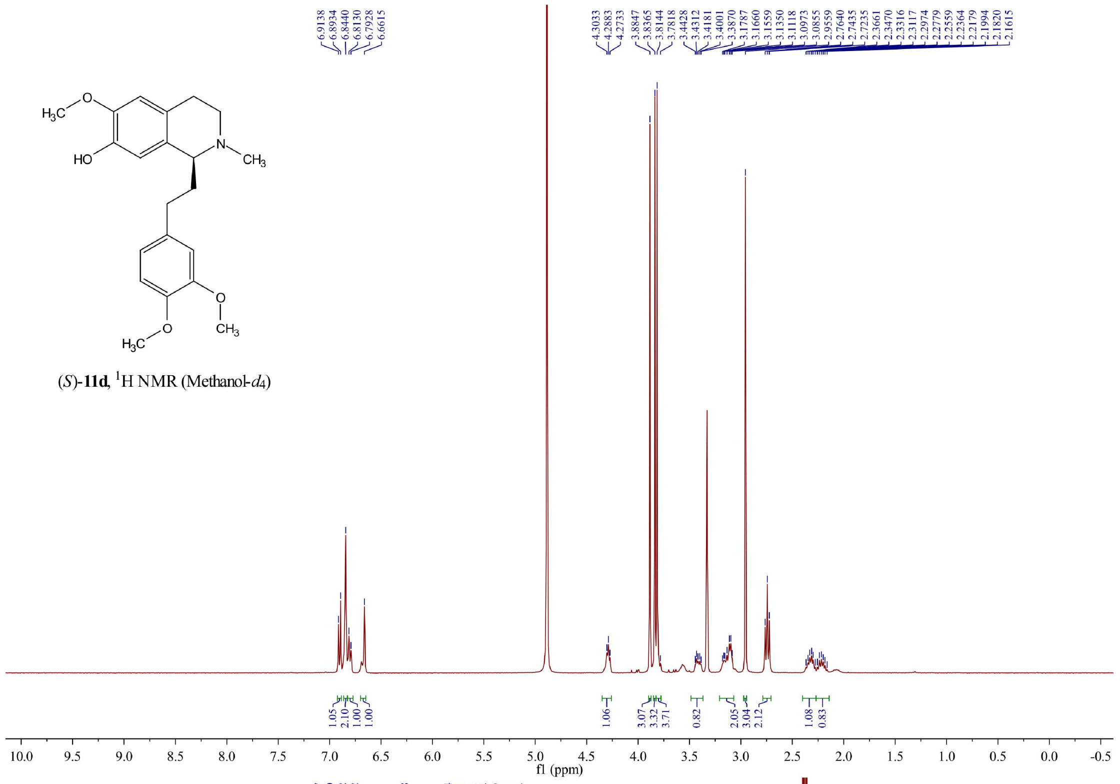

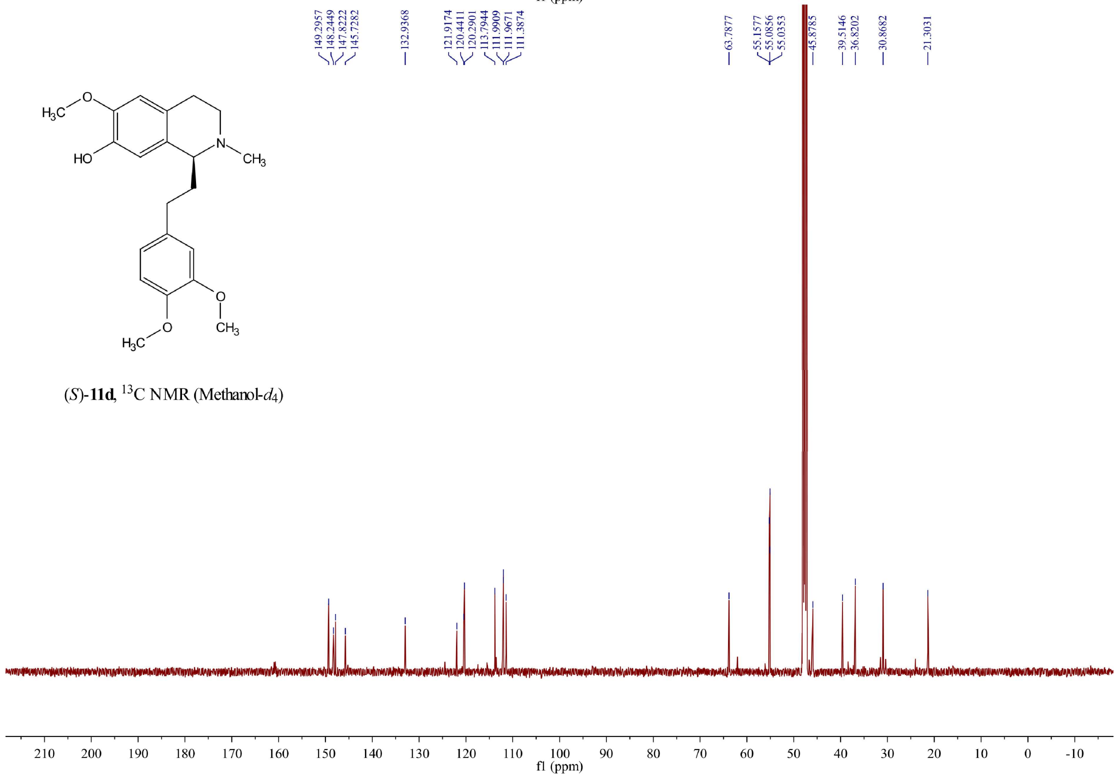

Supplementary Figure 30. ^{1}H NMR and ^{13}C NMR spectra of (*S*)-**11d**.

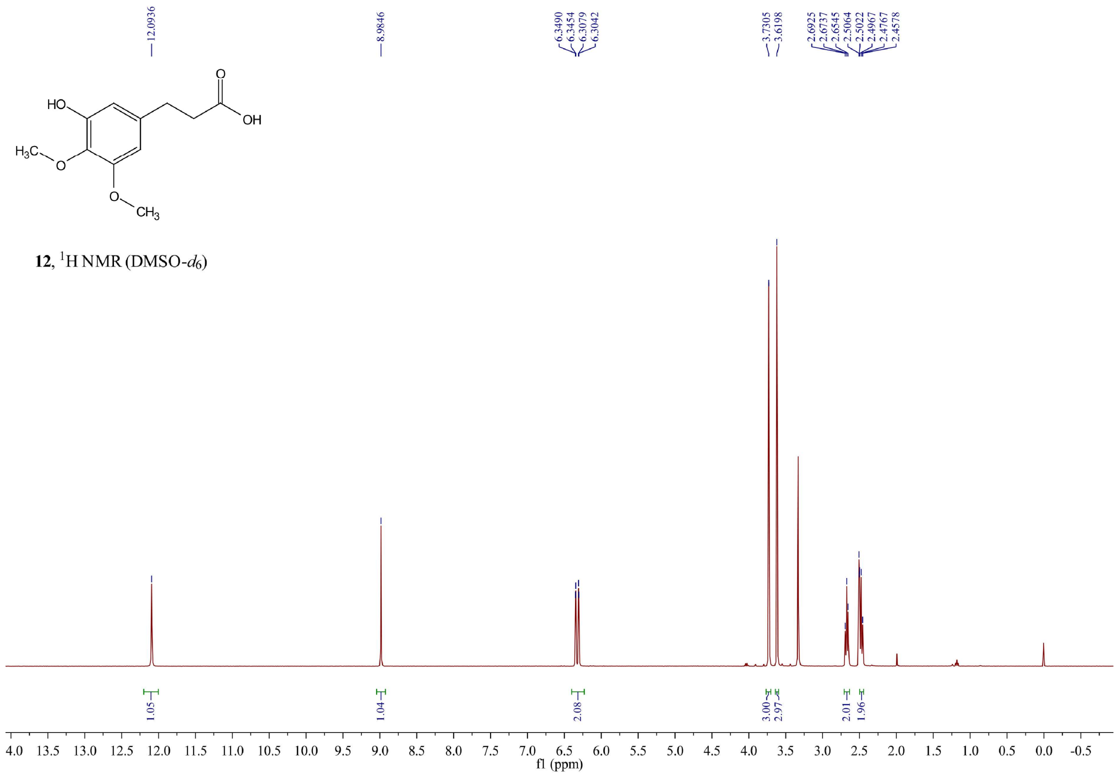

Supplementary Figure 31. ^{1}H NMR spectra of **12**.

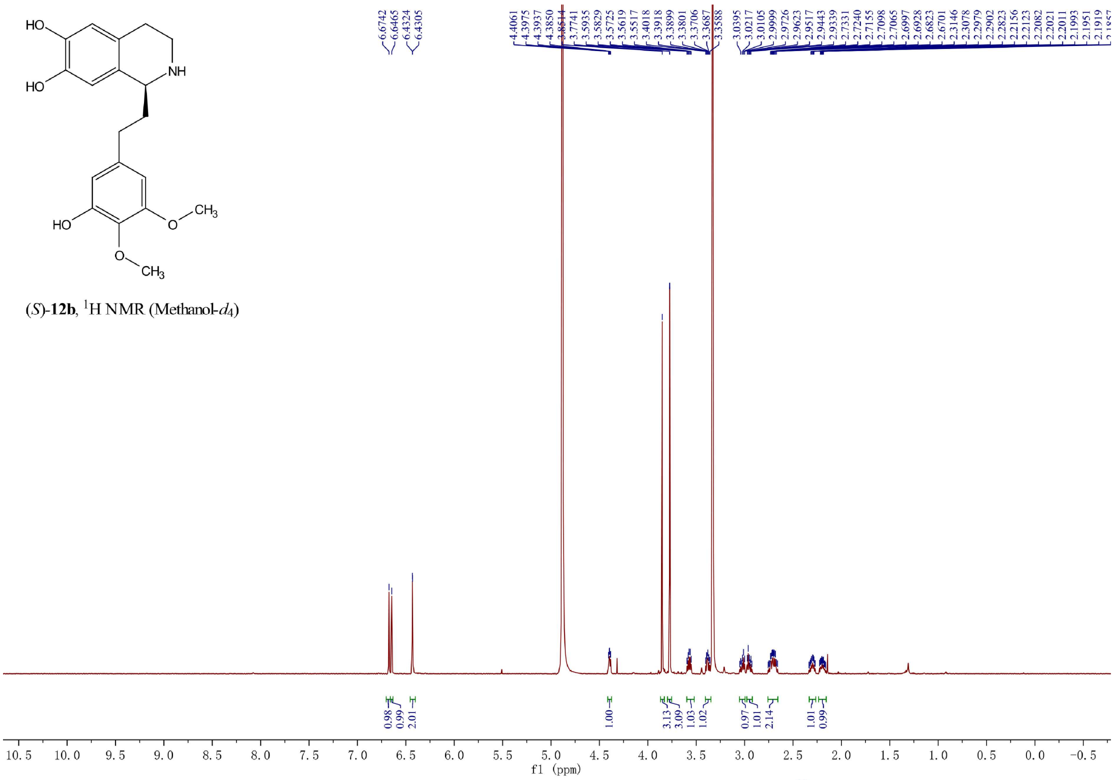

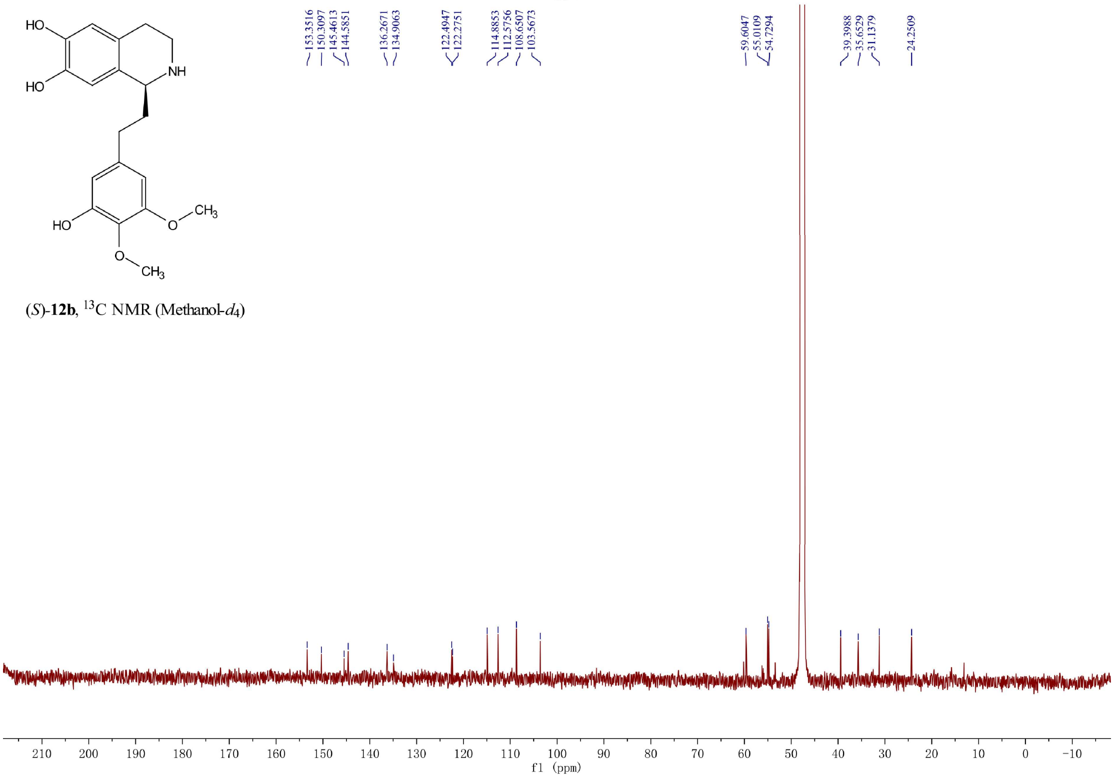

Supplementary Figure 32. ^{1}H NMR and ^{13}C NMR spectra of (*S*)-**12b**.

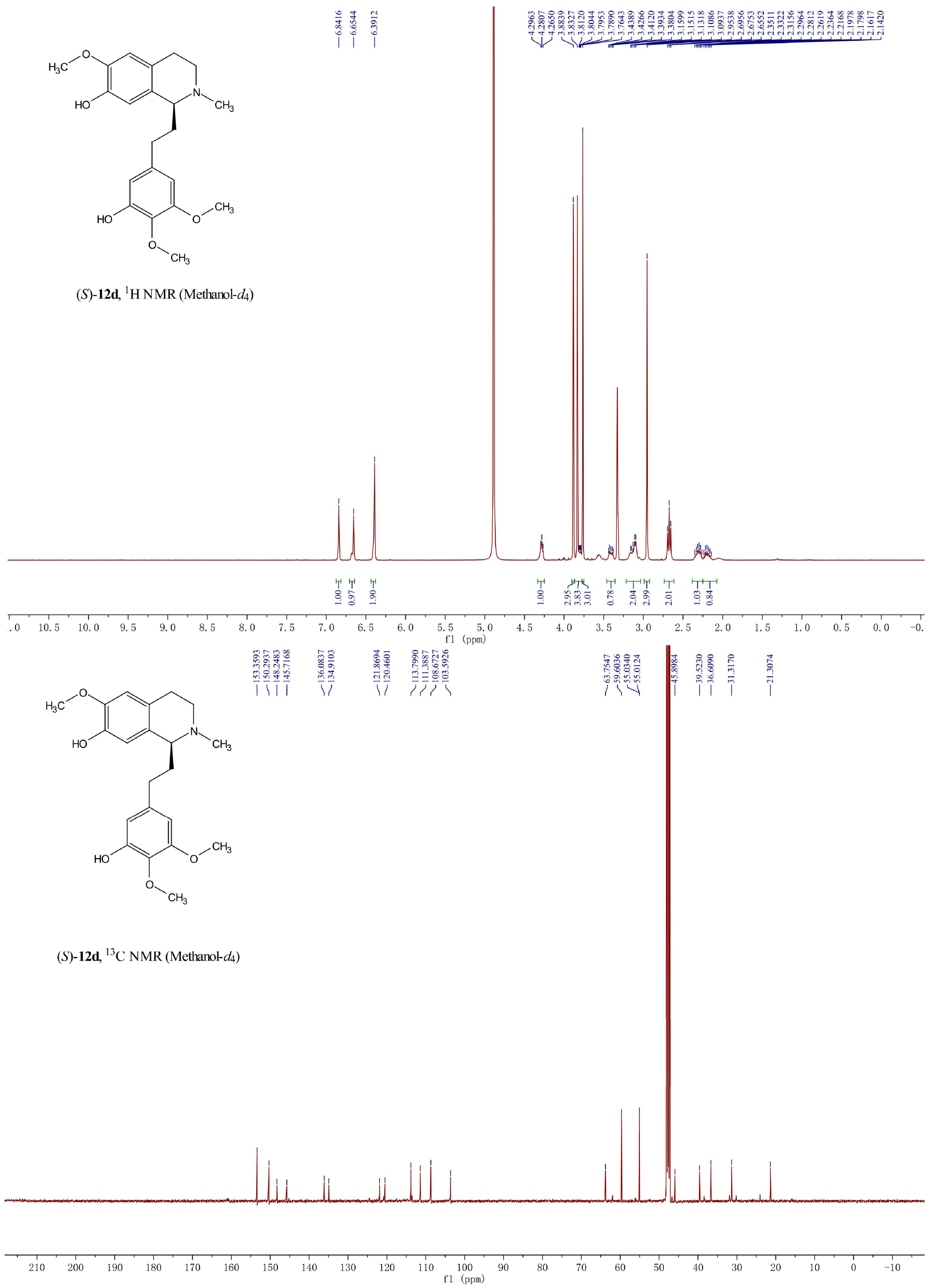

Supplementary Figure 33. ^{1}H NMR and ^{13}C NMR spectra of (*S*)-**12d**.

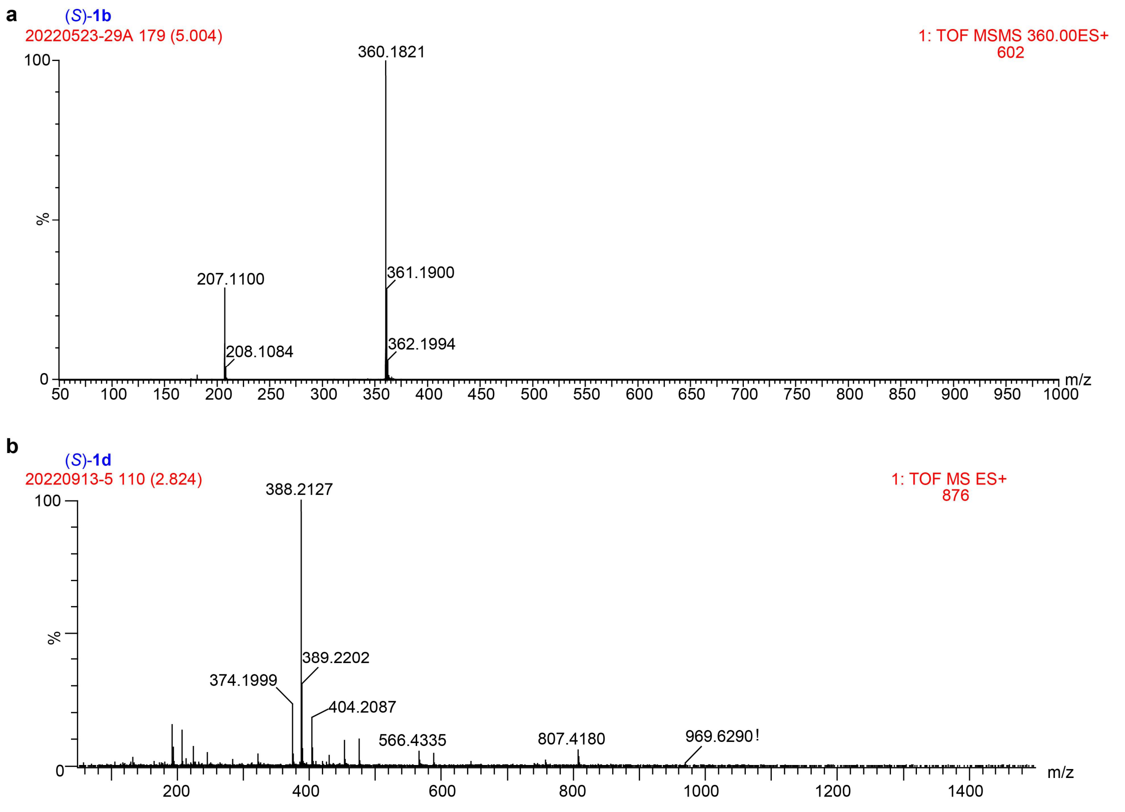

Supplementary Figure 34. HRMS spectra of (*S*)-**1b** and (*S*)-**1d**. **a** MS spectrum (positive) of (*S*)-**1b** showed *m/z* 360.1821, $[M+H]^+$ calcd. for $C_{20}H_{26}NO_5$, 360.1805. **b** MS spectrum (positive) of (*S*)-**1d** showed *m/z* 388.2127, $[M+H]^+$ calcd. for $C_{22}H_{30}NO_5$, 388.2118.

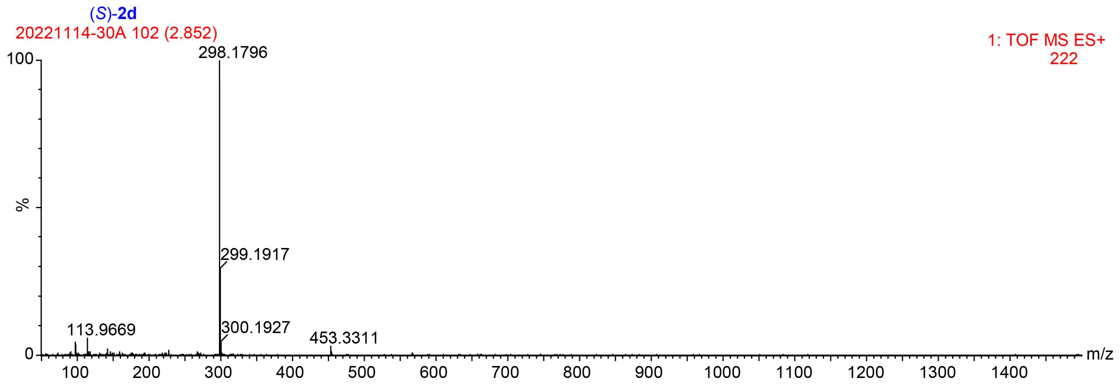

Supplementary Figure 35. HRMS spectra of (*S*)-**2d**. MS spectrum (positive) of (*S*)-**2d** showed *m/z* 298.1796, $[M+H]^+$ calcd. for $C_{19}H_{24}NO_2$, 298.1802.

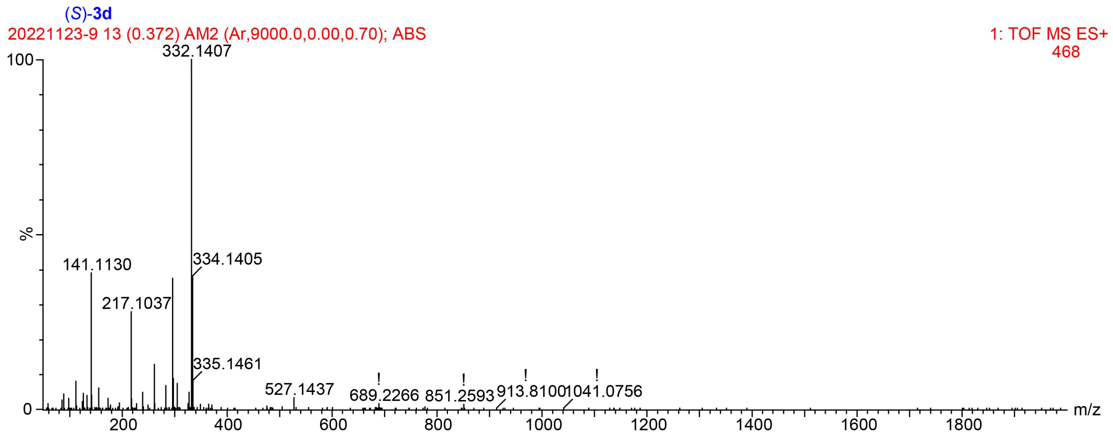

Supplementary Figure 36. HRMS spectra of (*S*)-**3d**. MS spectrum (positive) of (*S*)-**3d** showed *m/z* 332.1407, $[M+H]^+$ calcd. for $C_{19}H_{23}ClNO_2$, 332.1412.

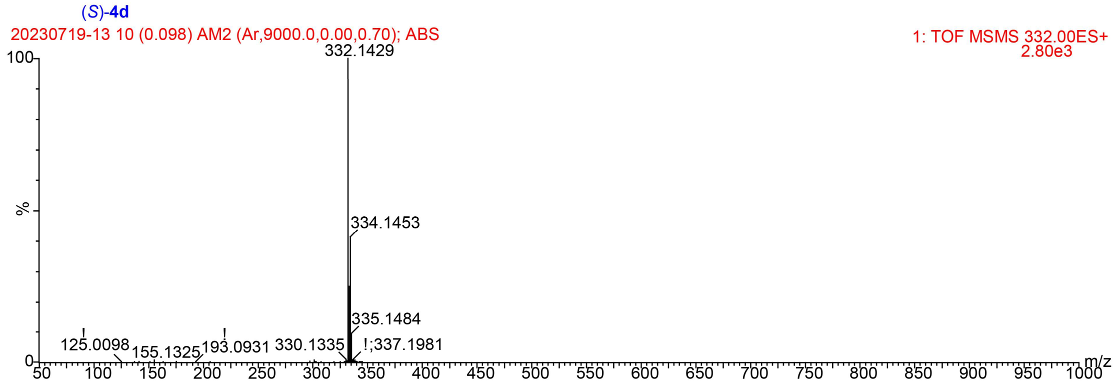

Supplementary Figure 37. HRMS spectra of (*S*)-**4d**. MS spectrum (positive) of (*S*)-**4d** showed *m/z* 332.1429, $[M+H]^+$ calcd. for $C_{19}H_{23}ClNO_2$, 332.1412.

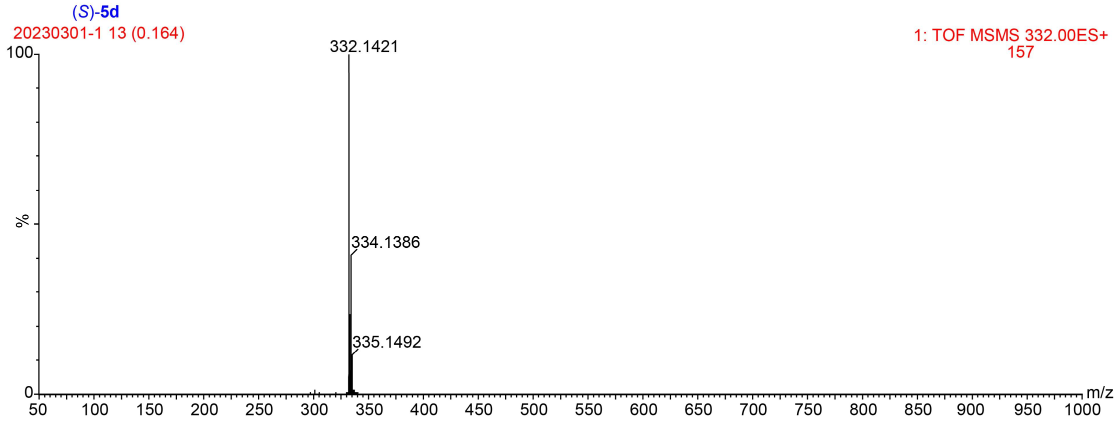

Supplementary Figure 38. HRMS spectra of (*S*)-**5d**. MS spectrum (positive) of (*S*)-**5d** showed *m/z* 332.1421, $[M+H]^+$ calcd. for $C_{19}H_{23}ClNO_2$, 332.1412.

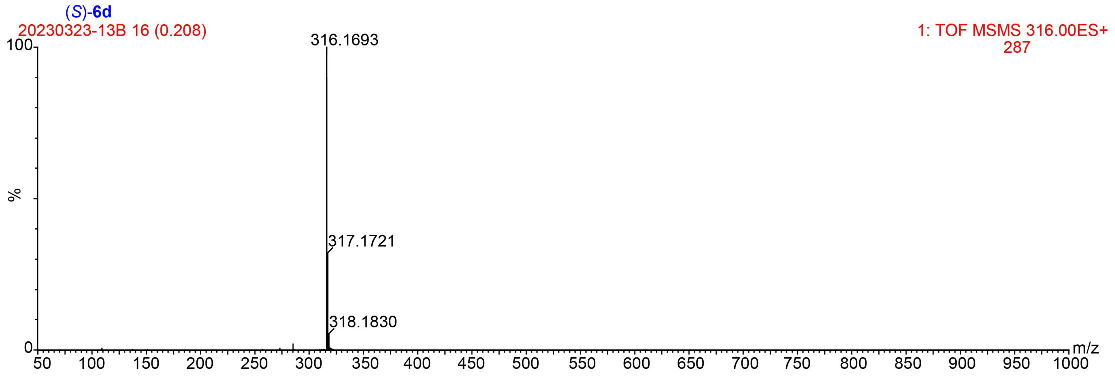

Supplementary Figure 39. HRMS spectra of (*S*)-**6d**. MS spectrum (positive) of (*S*)-**6d** showed *m/z* 316.1693, $[M+H]^+$ cacld. for $C_{19}H_{23}FNO_2$, 316.1707.

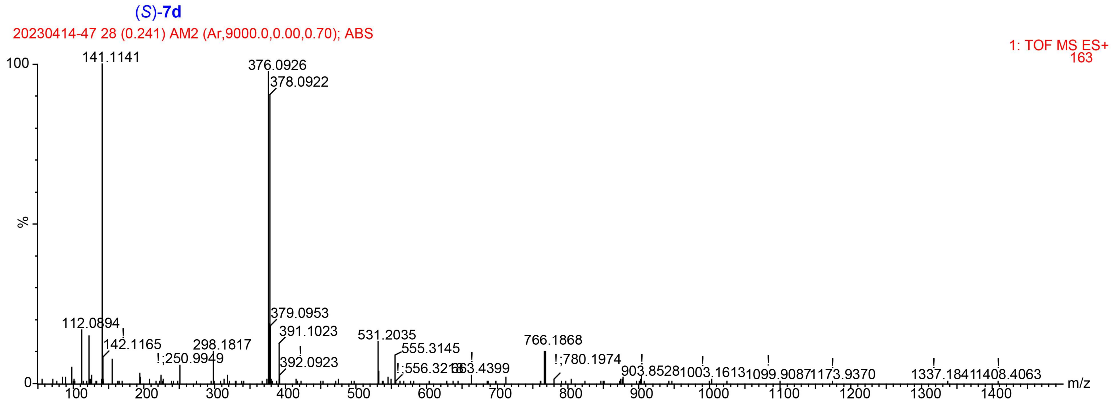

Supplementary Figure 40. HRMS spectra of (*S*)-**7d**. MS spectrum (positive) of (*S*)-**7d** showed *m/z* 376.0926, $[M+H]^+$ calcd. for $C_{19}H_{23}BrNO_2$, 376.0907.

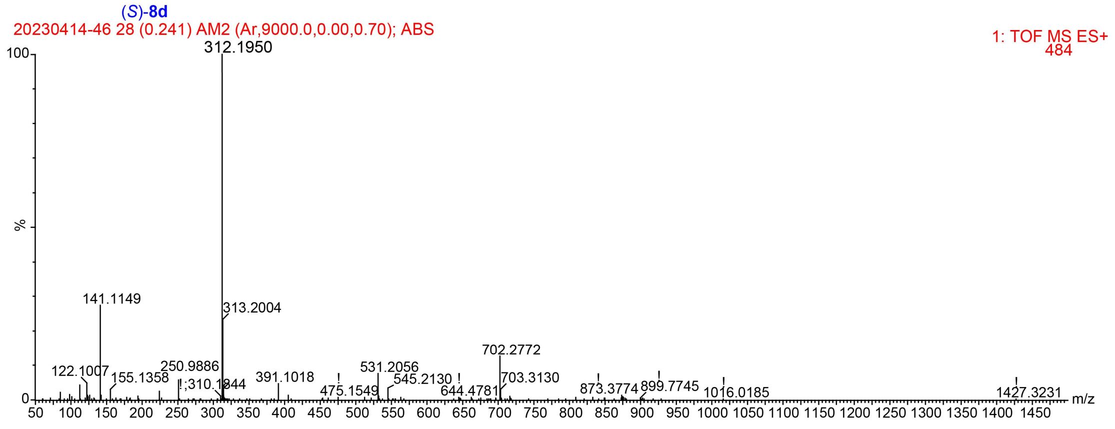

Supplementary Figure 41. HRMS spectra of (*S*)-**8d**. MS spectrum (positive) of (*S*)-**8d** showed *m/z* 312.1950, $[M+H]^+$ calcd. for $C_{20}H_{26}NO_2$, 312.1958.

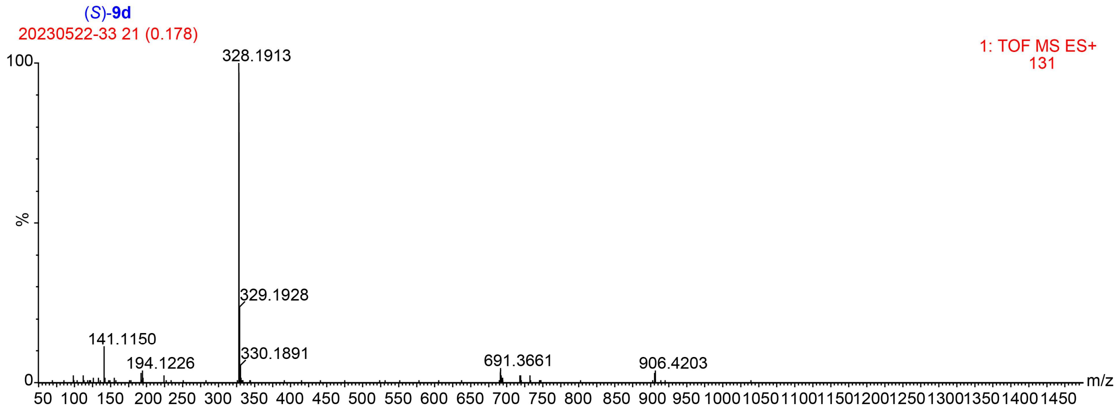

Supplementary Figure 42. HRMS spectra of (*S*)-**9d**. MS spectrum (positive) of (*S*)-**9d** showed *m/z* 328.1913, $[M+H]^+$ calcd. for $C_{20}H_{26}NO_3$, 328.1907.

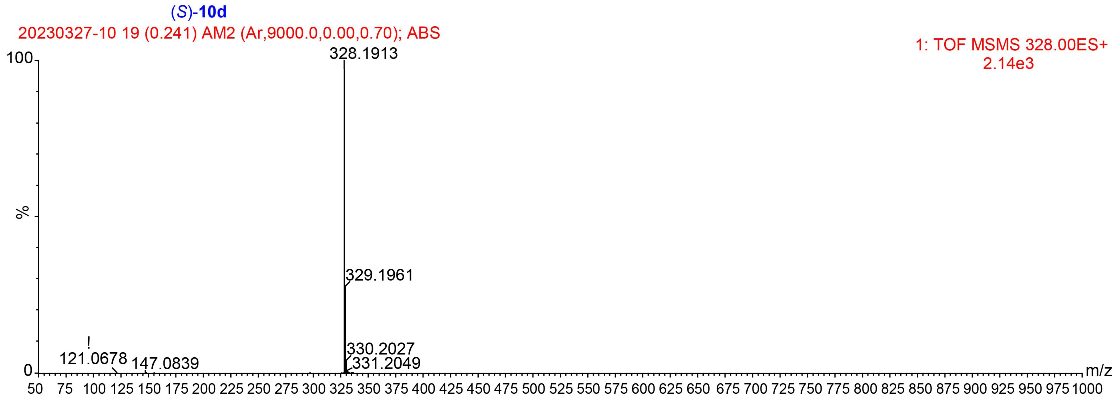

Supplementary Figure 43. HRMS spectra of (*S*)-**10d**. MS spectrum (positive) of (*S*)-**10d** showed *m/z* 328.1913, $[M+H]^+$ calcd. for $C_{20}H_{26}NO_3$, 328.1907.

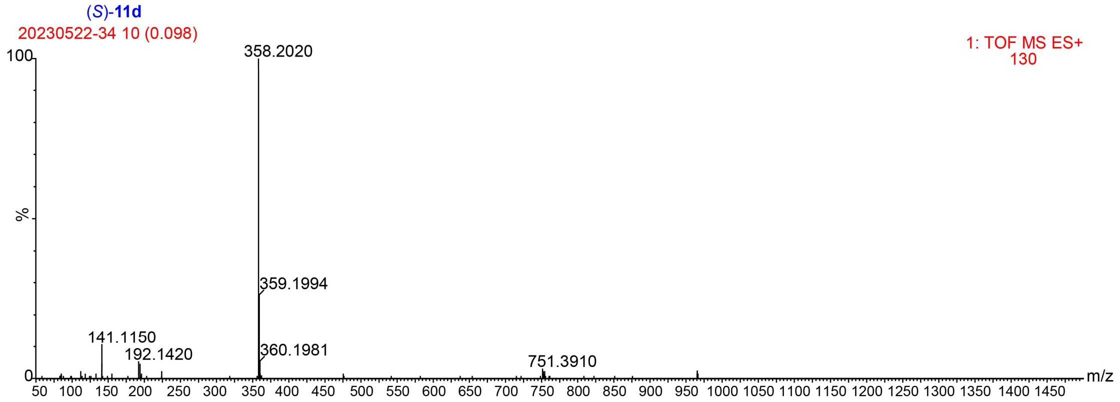

Supplementary Figure 44. HRMS spectra of (*S*)-**11d**. MS spectrum (positive) of (*S*)-**11d** showed *m/z* 358.2020, $[M+H]^+$ calcd. for $C_{21}H_{28}NO_4$, 358.2013.

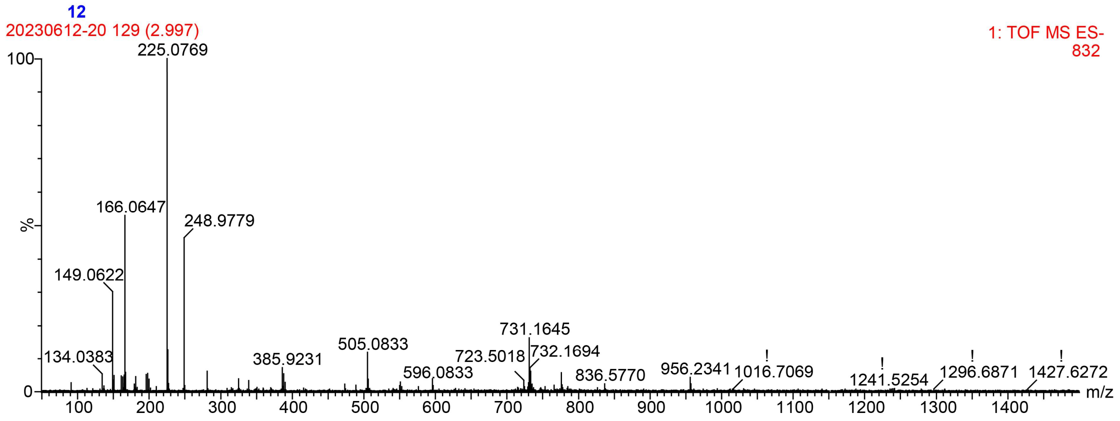

Supplementary Figure 45. HRMS spectra of **12**. MS spectrum (negative) of **12** showed *m/z* 225.0769, [M-H]$^-$ calcd. for $C_{11}H_{13}O_5$, 225.0768.

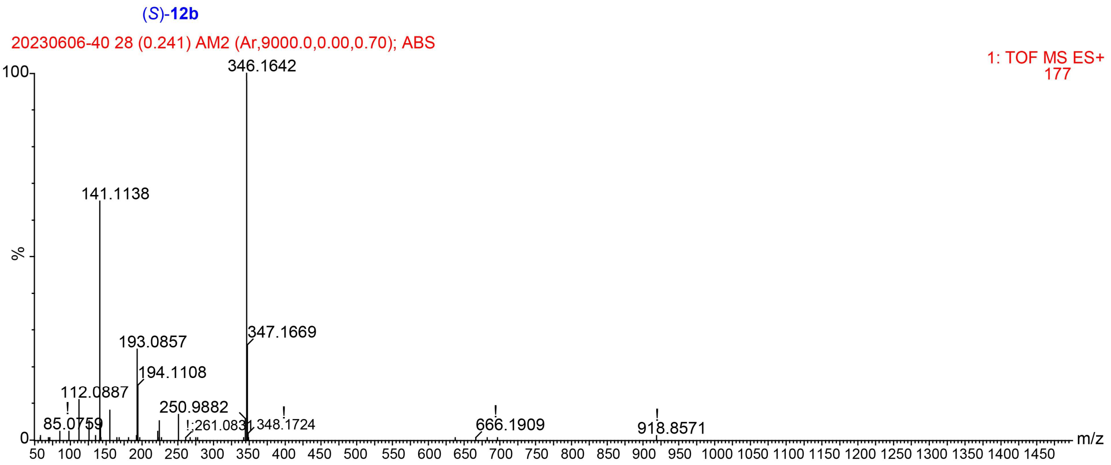

Supplementary Figure 46. HRMS spectra of (*S*)**-12b**. MS spectrum (positive) of (*S*)**-12b** showed *m/z* 346.1642, [M+H]$^+$ calcd. for $C_{19}H_{24}NO_5$, 346.1649.

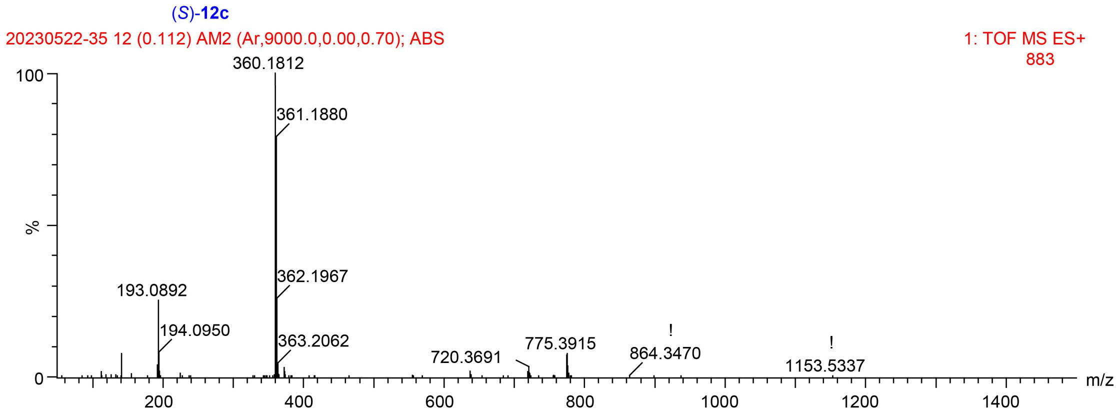

Supplementary Figure 47. HRMS spectra of (*S*)**-12c**. MS spectrum (positive) of (*S*)**-12c** showed *m/z* 360.1812, [M+H]$^+$ calcd. for $C_{20}H_{26}NO_5$, 360.1805.

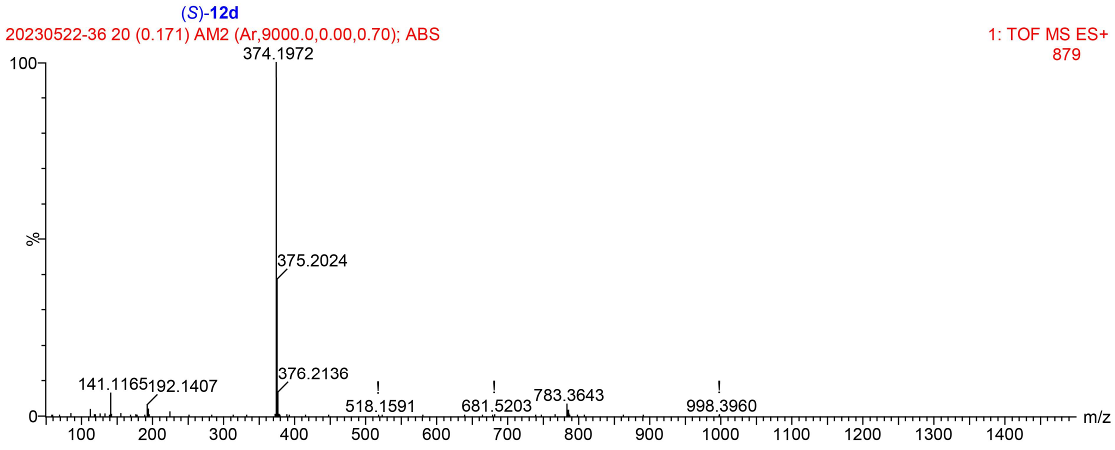

Supplementary Figure 48. HRMS spectra of (*S*)**-12d**. MS spectrum (positive) of (*S*)**-12d** showed *m/z* 374.1972, $[M+H]^+$ calcd. for $C_{21}H_{28}NO_5$, 374.1962.

Supplementary Reference:

1. Jakobsson, J.E., Gourni, E., Khanapur, S., Brito, B. & Riss, P.J. Synthesis and characterization in rodent brain of the subtype-selective NR2B NMDA receptor ligand [^{11}C]Ro04-5595 as a potential radiotracer for positron emission tomography. *Acs Omega* **4**, 9925-9931 (2019).
2. Nimgirawath, S. Synthesis of (±)-isoautumnaline and (±)-dysoxyline. *Aust. J. Chem.* **47**, 957-962 (1994).
